# Somatostatin 1.1 contributes to the innate exploration of zebrafish larva

**DOI:** 10.1038/s41598-020-72039-x

**Published:** 2020-09-17

**Authors:** Feng B. Quan, Laura Desban, Olivier Mirat, Maxime Kermarquer, Julian Roussel, Fanny Koëth, Hugo Marnas, Lydia Djenoune, François-Xavier Lejeune, Hervé Tostivint, Claire Wyart

**Affiliations:** 1 Sorbonne Université, Institut du Cerveau (ICM), Campus Hospitalier Universitaire Pitié-Salpêtrière, 47 bld de l’Hôpital, 75013 Paris, France; 2 Muséum National d’Histoire Naturelle (MNHN), CNRS UMR 7221, Paris, France; 3 Present Address: Institute of Neuroscience, University of Oregon, Eugene, OR USA; 4 Present Address: Cardiovascular Research Center, Massachusetts General Hospital, Charlestown, MA 02129 USA; 5 Present Address: Harvard Medical School, Boston, MA 02115 USA

**Keywords:** Neuroscience, Motor control, Spinal cord

## Abstract

Pharmacological experiments indicate that neuropeptides can effectively tune neuronal activity and modulate locomotor output patterns. However, their functions in shaping innate locomotion often remain elusive. For example, somatostatin has been previously shown to induce locomotion when injected in the brain ventricles but to inhibit fictive locomotion when bath-applied in the spinal cord in vitro. Here, we investigated the role of somatostatin in innate locomotion through a genetic approach by knocking out *somatostatin 1.1* (*sst1.1*) in zebrafish. We automated and carefully analyzed the kinematics of locomotion over a hundred of thousand bouts from hundreds of mutant and control sibling larvae. We found that the deletion of *sst1.1* did not impact acousto-vestibular escape responses but led to abnormal exploration. *sst1.1* mutant larvae swam over larger distance, at higher speed and performed larger tail bends, indicating that Somatostatin 1.1 inhibits spontaneous locomotion. Altogether our study demonstrates that Somatostatin 1.1 innately contributes to slowing down spontaneous locomotion.

## Introduction

In vertebrates, locomotion relies on neuronal networks called central pattern generators (CPGs) that coordinate the organized patterns of activity in motor neurons^1–3^. Although most fast motor actions computed by neural circuits are mediated by synaptic neurotransmitters, the modulation of locomotor patterns relies on neuromodulators such as serotonin, dopamine and neuropeptides, which allow an important versatility in network functions without requiring structural changes in circuit organization^4–6^. Neuropeptides can play an important role in modulating locomotor patterns by tuning activity of spinal interneurons and motor neurons. Neuropeptides are widely expressed in the central nervous system (CNS) with a high cell type specificity. However, their roles in modulating intrinsic locomotor patterns are still largely uncharacterized.

Somatostatin is a cyclic tetradecapeptide that was originally isolated from ovine hypothalamic extracts as a result of its ability to inhibit the release of growth hormone by pituitary cells^7^. Subsequent studies in mammals have revealed that somatostatin is produced in several tissues and play important roles in coordinating growth, development and metabolism^8–10^. Somatostatin is widely expressed in the CNS where it is mainly found in the hypothalamus^11–13^, amygdala^14,15^, preoptic area^16^, hippocampus^17–19^, striatum^20^, deeper layers of the cerebral cortex^21–23^, brainstem^24,25^ and the spinal cord^26–29^. Somatostatin has been shown to act as a neuromodulator involved in sleep^30^, learning^31,32^, food intake^33^, reproduction^34^, neuropathology^35^ and locomotor activity^36,37^. Somatostatin exerts its actions through specific receptors, which belong to the G protein-coupled receptor (GPCR) superfamily. In mammals, there are five somatostatin receptor subtypes (SSTR1-5)^38,39^.

The contribution of somatostatin to motor functions is suggested by several studies in mammals. Early reports have shown that intracerebroventricular administration of the peptide increased locomotor activity^36,37^, while somatostatin depletion induced by cysteamine produced opposite effects^36^, suggesting an excitatory action in motor control. However, in lamprey, bath application of somatostatin resulted in a reduction of locomotor network rhythm in a dose-dependent manner in the spinal cord during fictive locomotion^40^, indicating conversely a depressing effect on locomotion. The exact contribution of somatostatin on innate locomotion remains thereby unclear.

Recent studies have revealed in the spinal cord an interoceptive pathway relying on cerebrospinal fluid-contacting neurons (CSF-cNs) that can detect spinal curvature and in turn, modulate locomotion via GABAergic projections on CPG neurons^41–44^. In mouse and zebrafish, two populations of spinal CSF-cNs, namely dorsal and ventral CSF-cNs, have been characterized on the basis of both their origin, morphology^42^ and location^42,45,46^. Interestingly, in zebrafish, dorsal CSF-cNs, that are selectively recruited upon lateral tail bending during swimming^41^, express *somatostatin 1.1*, the fish counterpart of the somatostatin gene in mammals^47^, and project onto V0-v interneurons known to control slow locomotion^42,43^.

The zebrafish model organism offers the opportunity to combine genetic manipulations with high-throughput behavioral analysis on hundreds of thousands of stereotyped locomotor events from hundreds of animals at the larval stage^48,49^. Five days post fertilization (dpf), zebrafish larvae exhibit a ‘beat-and-glide’ swimming with discrete bouts of swim separated by long resting periods^48–50^. Their exploration consists of slow bouts propelling them either forward or with an angle, defined as slow swims and routine turns respectively. In response to a threatening stimulus, larval zebrafish perform escape responses characterized by a C-start and an initial high tail beat frequency, with powerful tail bends, followed by gradual decrease in both amplitude and frequency^51–53^.

Here we investigated the role of Somatostatin 1.1 on innate locomotion in zebrafish larvae. We generated a *sst1.1* mutant using CRISPR/Cas9-mediated genome editing. To achieve the analysis depth necessary for the detection of modifications in both spontaneous locomotion and escape responses, we developed high-throughput behavior assays enabling us to record and analyze up to ~ 120,000 locomotor events at either high frequency following acousto-vestibular (AV) stimulus, or during long-scale time. We compared the kinematics of *sst1.1* mutant larvae to their wild-type (WT) siblings in three basic maneuvers: AV escape responses in fast locomotor regime, or forward swim and routine turn bouts occurring during slow swimming exploration. Our results indicate that Somatostatin 1.1 inhibits locomotion in the slow regime, in particular during forward swims by reducing amplitude of tail bends, bout duration and distance travelled. We discuss the possible circuits involved in this modulation.

## Results

### Generation of the *sst1.1* mutant

**Figure 1:**
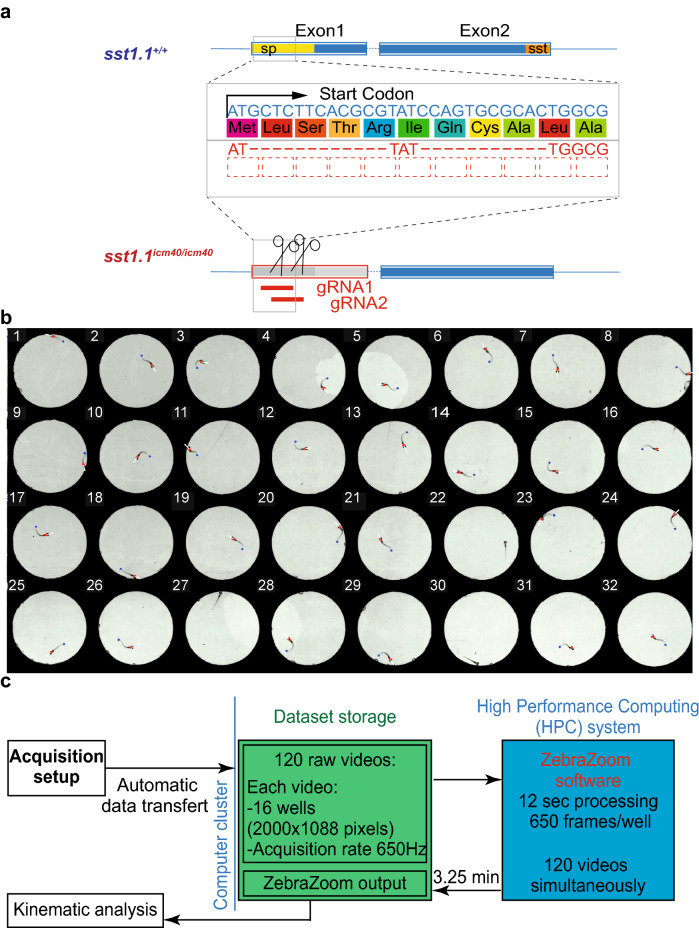
Generation of the *sst1.1* mutant and a high-throughput assay for kinematic analysis. (**a**) Generation of the mutated allele *icm40* of the *somatostatin 1.1* gene (*sst1.1*^*icm40*^) using CRISPR/Cas9-mediated genome editing. Top, genomic organization of the *sst1.1* wild type gene with the coding sequence (yellow) of the signal peptide (sp, 24 amino acids) and (orange) mature somatostatin peptide (sst, 26 amino acids). Bottom, the *icm40* mutated allele results from 23-bp deletion that impairs the start codon, and induces a frameshift in the sst1.1 coding sequence. (**b**) We developed a high throughput behavior assay to simultaneously record and at high frequency (350 Hz) 32–88 freely-swimming zebrafish larvae in individual circular swim arenas of 15-mm diameter. Behavioral recordings were tracked using the ZebraZoom algorithm^49^, which detects the head direction (white line), the body position (red dots) and the tail end position (blue dot). See Supplementary Video S1. (**c**) Illustration of the pipeline for image processing in order to achieve rapid high-throughput analysis of kinematics between mutants and their control siblings. See “Methods”.

To investigate whether Somatostatin 1.1 release affects the kinematics of locomotion, we generated a *sst1.1* mutant using CRISPR/Cas9-mediated genome editing (Fig. 1a, “Methods”). We designed guide RNAs targeting specifically the beginning of the coding sequence that led to a 23 base pair deletion removing the start codon and leading to an early frameshift. We isolated this allele that is referred to as *sst1.1*^*icm40*^.

### High-throughput quantification of locomotor defects in mutant zebrafish larvae

Inter-clutches differences often account for most of the variability in behavior. To detect locomotor defects associated with loss of *somatostatin 1.1*, we therefore compared the locomotion kinematics in *sst1.1*^*icm40/icm40*^ mutants to the ones in WT siblings obtained from incrossing adult zebrafish heterozygous for the mutation. We systematically analyzed kinematic parameters within the same clutches prior to genotyping individuals in order to blind experiments (see “Methods”). Inter-clutches variations are very large in behavioral datasets and the detection of minor locomotor defects due to the loss of function of a single peptide such as Somatostatin 1.1 required to compare the behavior of hundreds of larvae from the same clutches.

First, we developed a high throughput behavior assay to simultaneously record with two cameras in parallel up to 88 fish isolated in individual wells and performing thousands of swimming events acquired at 100–200 Hz and over tens of minutes (see “Methods”). Second, we improved the source code of our tracking software ZebraZoom^41,49,53^ to reduce the tracking error rate (under 0.5%) by adjusting the configuration parameters (Fig. 1b, “Methods”). Finally, we adapted the source code of ZebraZoom to be executed on a High-Performance Computing (HPC) system equipped with numerous processors. We automated the process of the big dataset management and storage to be accessible by the HPC system, thereby allowing to promptly process large datasets (e.g. ~ 120,000 swimming bouts) at high speed (typically processing 3,250 frames per min per well and running 120 wells in parallel) (Fig. 1c, “Methods”). By reorganizing the pipeline of the ZebraZoom algorithm, we managed to simultaneously track hundreds of videos. Altogether, we implemented a powerful high throughput pipeline for acquiring and tracking behavior in order to efficiently quantify locomotor defects of mutant zebrafish larvae. Compared to online tracking, our pipeline authorizes the later inspection of videos in order to verify tracking and detect additional defects in 3D posture that may have been missed from the sole analysis of the head direction and tail angle^44,54^.

### *sst1.1* mutant larvae perform proper escape responses

**Figure 2:**
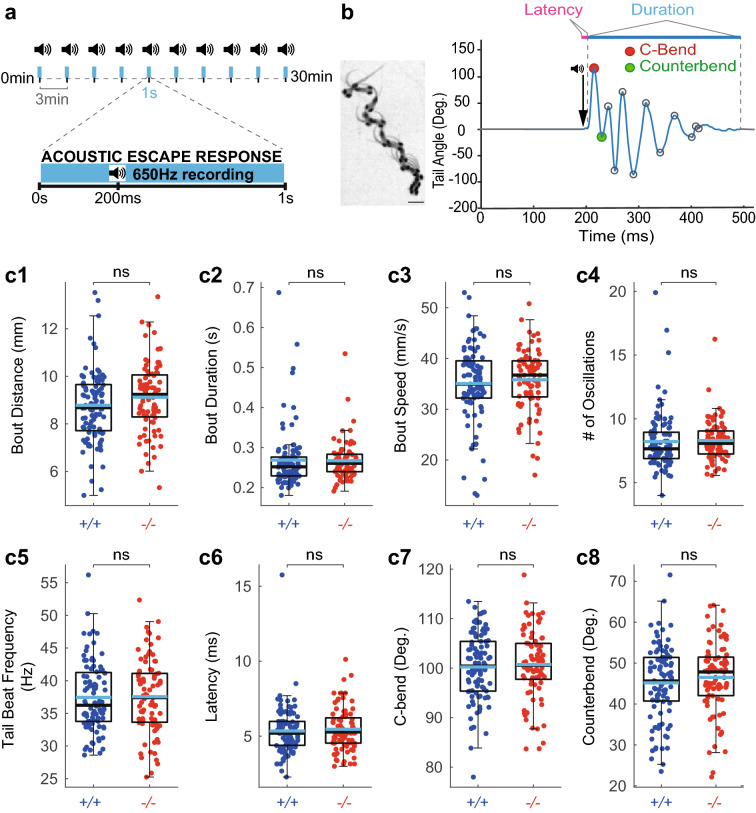
Somatostatin 1.1 does not contribute to the kinematic of acousto-vestibular escape responses. (**a**) Experimental paradigm used to record the escape response of 5 dpf zebrafish larvae to AV stimuli delivered for 5 ms at 500 Hz. Larvae were subjected to 10 trials interspaced by 3 min. Behavioral responses were recorded for 1 s at 650 Hz. (**b**) Left, Superimposed images illustrating the sequence of movements during a typical AV escape response (scale bar: 1 mm). Right, tail angle trace over time of escape response showing the large C-bend (red circle) followed by the counter bend (green circle) characteristic of the escape response. Latency is defined as the delay between the time of the acoustic stimulus and the onset of the behavioral response; Tail beat frequency (TBF) calculated as the number of oscillations divided by the bout duration. (**c1**–**c8**) No difference in AV escape responses between *sst1.1i*^*cm40/icm40*^ mutants (‘−/−’, 84 larvae from 4 clutches and 761 escapes) and WT siblings (‘+/+’, 96 larvae from 4 clutches and 861 escapes): distance (**c1**, mean ± SEM 9.12 ± 0.16 mm versus 8.73 ± 0.15 mm in WTs; Wald χ^2^(1) = 1.047, *p* = 1), bout duration (**c2**, mean ±  SEM 0.27 ± 0.01 s versus 0.27 ± 0.01 s in WTs; Wald χ^2^(1) = 0.996, *p* = 1), speed (**c3**, mean ± SEM 35.73 ± 0.67 mm/s versus 34.97 ± 0.78 mm/s in WTs; Wald χ^2^(1) = 0.457, *p* = 1), number of oscillations (**c4**, mean ± SEM 8.27 ± 0.17 versus 8.22 ± 0.23 in WTs; Wald χ^2^(1) = 0.534, *p* = 1), tail beat frequency (**c5**, mean ± SEM 37.56 ± 0.62 Hz versus 37.45 ± 0.54 Hz in WTs; Wald χ^2^(1) = 0.001, *p* = 1), latency (**c6**, mean ± SEM 5.45 ± 0.16 ms versus 5.34 ± 0.17 ms in WTs; Wald χ^2^(1) = 7.297, *p* = 0.050), C-bend amplitude (**c7**, mean ± SEM 100.60 ± 0.76 degrees versus 100.20 ± 0.71 degrees in WTs; Wald χ^2^(1) = 0.023, *p* = 1) and counter bend amplitude (**c8**, mean ± SEM 46.51 ± 0.95 degrees versus 45.15 ± 0.93 degrees in WTs; Wald χ^2^(1) = 4.167, *p* = 0.298). Each dot represents the value for one larva averaged over ten trials. Median values are indicated in black, and means in blue; Statistical tests used: Wald chi-square test followed by M_eff_ correction for multiple comparisons. M_eff_ = 7.230.

We next investigated the role of Somatostatin 1.1 in the fast locomotor regime. We investigated escape responses induced by AV stimuli (5 ms-long sine wave at 500 Hz, see “Methods” and Fig. 2a). All zebrafish larvae were subjected to 10 trials interspaced by 3 min (Fig. 2a) and we analyzed escape kinematic parameters averaged across trials. Escape response behavior is highly stereotyped starting with a fast short latency C-start followed by a large counter bend in the opposite direction, followed by a series of oscillations at high tail beat frequency^50^ (Fig. 2b). We measured eight kinematics parameters for each response: distance travelled (Fig. 2c1), duration (Fig. 2c2), speed (Fig. 2c3), number of oscillations (Fig. 2c4), average tail beat frequency (Fig. 2c5), latency (Fig. 2c6), C-bend amplitude (Fig. 2c7) and counter bend amplitude (Fig. 2c8). We did not observe any difference between *sst1.1*^*icm40/icm40*^ mutant larvae compared to WT control siblings (Fig. 2c1–c8). Overall, we conclude that Somatostatin 1.1 does not contribute to the kinematics of the fast escape response.

### Somatostatin 1.1 does not contribute to the rate of exploration

**Figure 3:**
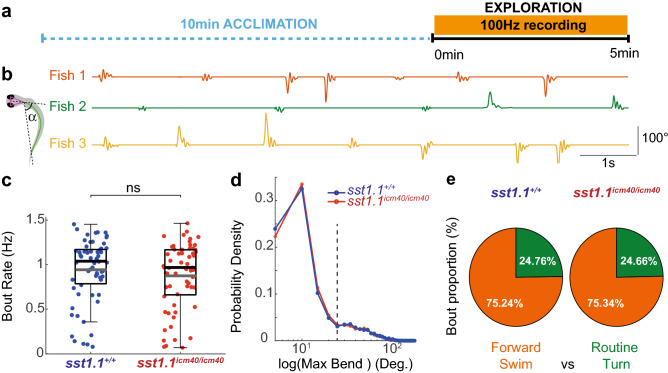
Somatostatin 1.1 does not contribute to the bout rate nor to the ratio of forward bouts and turns during exploration. (**a**) Experimental paradigm used to record spontaneous slow swim in 5-dpf zebrafish larvae over 5 min at 100 Hz after 10 min of acclimation. (**b**) The kinematic analysis relies on the measure of the tail angle α over time as shown for 3 different larvae. (**c**) Bout rate is similar for *sst1.1*^*icm40/icm40*^ mutant larvae and control WT siblings (Grey line: mean ± SEM. 0.91 ± 0.04 Hz, N = 58 larvae; 0.95 ± 0.04 Hz, N = 62 larvae; Wald χ^2^(1) = 0.084, *p* = 0.771). Black lines: medians. (**d**) Probability density function of the absolute maximal bend amplitude for all bouts of *sst1.1*^*icm40/icm40*^ mutant larvae (red) versus WT siblings (blue). Forward swims and routine turns were sorted with a 25-degree threshold (dashed line). (**e**) The ratio of forward swims (orange) and routine turns (green) is similar for *sst1.1*^*icm40/icm40*^ mutant larvae and WT siblings.

Five-dpf zebrafish larvae explore their environment by generating a series of locomotor bouts typically comprising forward swim bouts and routine turns, all occurring in the slow regime at 1–2 Hz (Fig. 3). We first investigated the consequences of *sst1.1* loss of function on the exploration rate of zebrafish larvae. We recorded the spontaneous slow swimming of 5-dpf larvae from the incrosses of *sst1.1*^*icm40/*+^ mutants (F3) at 100 Hz over 5 min (Fig. 3a,b). We compared the exploratory swim kinematics of *sst1.1*^*icm40/icm40*^ mutant larvae (n = 58 larvae, 16,043 bouts) to control siblings (n = 62 larvae, 17,834 bouts). We used the tail angle over time to identify individual bouts and quantify kinematic parameters (Fig. 3b). First, we tested whether *sst1.1* loss affects bout rate during exploration. We found no difference in swim bout rate for *sst1.1*^*icm40/icm40*^ mutant larvae compared to their WT siblings (mean ± SEM: 0.91 ± 0.04 Hz, N = 58 mutant larvae; mean ± standard error of the mean (SEM): 0.95 ± 0.04 Hz, N = 62 control WT siblings, see Fig. 3c).

We evaluated whether *sst1.1* loss affects the proportion of bout types occurring spontaneously, i.e. forward swims and routine turns. As a first approximation, we chose to distinguish forward swim and routine turn by using a 25-degree cut-off on the maximal bend amplitude (Fig. 3d). Using this categorization (Fig. 3e), we found that in our arenas, swim bouts occurred as forward swims in three quarter of cases, and routine turn for the remaining quarter in *sst1.1*^*icm40/icm40*^ mutants as in control WT siblings (mutant: 75.34% of forward swims versus 24.66% of routine turns; WT: 75.24% versus 24.76%). Altogether, these results indicate that *somatostatin 1.1* loss does not affect the bout rate nor the distribution of bout types during exploration.

### Somatostatin 1.1 innately inhibits locomotion during forward swims

**Figure 4:**
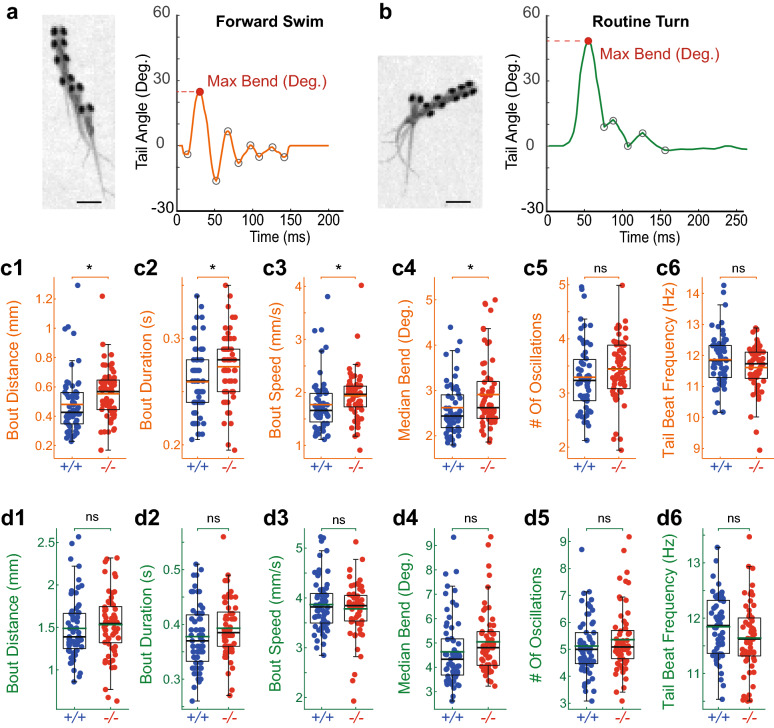
*Somatostatin 1.1* null mutant larvae explore more by deploying longer and faster forward swims. (**a**) Left, Superimposed images illustrating the sequence of positions observed during a forward swim (scale bar, 1 mm); right, typical forward swim showing the tail angle trace over time with a maximal bend amplitude below 25 degrees. Subsequent peaks (grey circles) correspond to each bend amplitude. (**b**) Left, superposed images illustrating the sequence of movements during routine turn (scale bar, 1 mm); right, tail angle trace over time of typical routine turn showing the maximal bend amplitude above 25 degrees. (**c1**–**c6**) Forward swims in *sst1.1*^*icm40/icm40*^ mutant (red, ‘−/−’) had larger distance traveled (**c1**, mean ± SEM 0.56 ± 0.02 mm versus 0.48 ± 0.03 mm in WTs; Wald χ^2^(1) = 7.340, *p* = 0.027), bout duration (**c2**, mean ± SEM 0.27 ± 0.004 s versus 0.26 ± 0.004 s in WTs; Wald χ^2^(1) = 7.171, *p* = 0.030), speed (**c3**, mean ± SEM 1.96 ± 0.06 mm/s versus 1.78 ± 0.07 mm/s in WTs; Wald χ^2^(1) = 6.955, *p* = 0.033) and median bend amplitude (**c4**, mean ± SEM 2.88 ± 0.10 degrees versus 2.57 ± 0.07 degrees in WTs; Wald χ^2^(1) = 6.529, *p* = 0.042) but no difference in number of oscillations (**c5**, mean ± SEM 3.47 ± 0.07 versus 3.29 ± 0.08 in WTs; Wald χ^2^(1) = 2.789, *p* = 0.380), tail beat frequency (**c6**, mean ± SEM 11.66 ± 0.09 Hz versus 11.84 ± 0.10 Hz in WTs; Wald χ^2^(1) = 1.785, *p* = 0.726). Black lines, median; Orange lines, mean. M_eff_ = 4.003. (**d1**–**d6**) Routine turns were undistinguishable in *sst1.1*^*icm40/icm40*^ mutant larvae compared to WTs siblings: Bout distance (**d1**, mean ± SEM 1.57 ± 0.04 mm versus 1.49 ± 0.05 mm in WTs; Wald χ^2^(1) = 1.910, *p* = 0.755); bout duration (**d2**, mean ± SEM 0.38 ± 0.01 s versus 0.37 ± 0.01 s in WTs; Wald χ^2^(1) = 4.025, *p* = 0.203); speed (**d3**, mean ± SEM 3.86 ± 0.05 mm/s versus 3.86 ± 0.07 mm/s in WTs; Wald χ^2^(1) = 0.021, *p* = 1); median bend amplitude (**d4**, 4.87 ± 0.14 degrees versus 4.60 ± 0.17 degrees in WTs; Wald χ^2^(1) = 1.853, *p* = 0.784); number of oscillations (**d5**, 5.42 ± 0.16 versus 5.12 ± 0.13 in WTs; Wald χ^2^(1) = 2.464, *p* = 0.526); tail beat frequency (**d6**, 11.68 ± 0.08 Hz versus 11.84 ± 0.07 Hz in WTs; Wald χ^2^(1) = 2.054, *p* = 0.686). Black lines, median; Green lines, mean. Each point represents the median for one fish over the 5 min-long recording. M_eff_ = 4.518. Comparison between 62 WT larvae from 4 clutches (17 834 bouts) and 58 *sst1.1*^*icm40/icm40*^ mutant larvae from 4 clutches (16 043 bouts) were tested with Wald χ^2^.

To probe whether Somatostatin 1.1 specifically contributes to the different exploratory bout types, we analyzed detailed kinematic parameters of forward swims and routine turns in *sst1.1*^*icm40/icm40*^ mutant and WT siblings. Slow forward swims consist of small and symmetrical side-to-side bends of the tail to propel the fish forward (maximal amplitude typically below 25°, see Fig. 4a). Conversely, routine turns correspond to low speed turns, characterized by a large initial bend (amplitude typically above 25°, see Fig. 4b), followed by swimming in the new direction imposed by the first bend^50^. We analyzed the kinematics based on the head position and tail angle^49^. For each bout, we extracted bout distance, duration, number of oscillations, median bend amplitude, speed and tail beat frequency (Fig. 4c,d). The forward swims of *sst1.1*^*icm40/icm40*^ mutants exhibited an increase in bout distance (16.5%, Fig. 4c1) and, to a smaller extent, in bout duration (6.5%, Fig. 4c2), resulting in an overall 10.5% increase in speed (Fig. 4c3) compared to WT siblings. This increase in speed was associated with larger bend amplitudes (12.2% increase, Fig. 4c4, Supplementary Fig. 1) that was consistent across clutches (Supplementary Fig. 2). Conversely, we observed no difference in any kinematic parameter between the two groups during routine turns that constitute one quarter of the spontaneous bouts in our assay (Fig. 4d1–d6). Altogether, these results reveal that Somatostatin 1.1 innately inhibits locomotion by reducing the amplitude of tail bends and bout duration, resulting in reduced travelled distance and speed of forward swims during spontaneous locomotion.

## Discussion

This study investigated the role of Somatostatin 1.1 on innate locomotion. To quantify the innate locomotion on over a hundred of thousand bouts acquired from hundreds of zebrafish larvae, we developed high throughput behavior assays to simultaneously track hundreds of animals, and analyze the kinematics of hundreds of thousands of bouts with high accuracy and speed. We investigated the kinematics of *sst1.1* knockout mutant larvae performing slow forward swims, routine turns and fast acousto-vestibular escape responses. Our results provide new insights for the neuromodulatory function of Somatostatin 1.1 on innate locomotion in zebrafish larvae. We found that Somatostatin 1.1 inhibits slow locomotion by innately reducing distance travelled, locomotor speed and amplitude of tail bends.

Previous studies had revealed complex modulations operated by somatostatin. *Somatostatin* knockouts did not show impaired spontaneous locomotion^32^, while behavioral studies on SSTR2-invalidated mice indicated an impairment of motor coordination^55^. In mammals, intracerebroventrical injection^36^ as well as injection in the amygdaloid complex^56^ of low doses of somatostatin increased motor activity, while higher doses reduced it^56^. Furthermore, the subcutaneous administration of cysteamine, a somatostatin-depleting agent, markedly decreased motor activity^36,37^. These observations suggest that somatostatin release could induce locomotion in a concentration-dependent manner. However, bath application of somatostatin on the lamprey spinal cord was recently shown in vitro to inhibit fictive locomotion by reducing burst rate^40^. Our results here confirm the latter findings. We find that Somatostatin 1.1 inhibits innate locomotion by reducing speed and distance travelled. Interestingly, the reduction in speed was associated with a reduction in bend amplitude but not in tail beat frequency, suggesting that the effect of Somatostatin 1.1 on speed might differ between these experimental models. Our study revealed a moderate but consistent inhibitory effect of Somatostatin 1.1 during innate locomotion. The subtlety of the phenotype in *sst1.1*^*icm40*^ mutants may be due to the compensatory action of the other paralogs of the somatostatin family^47^ (*sst1.2, sst2, sst5, sst6 and cort*) as observed for other deleterious mutations^57,58^. Further investigations involving the comparison with gene knockdowns shall be performed to determine whether such mechanisms occur for *sst1.1*.

We analyzed and compared fine swimming kinematics to detect the locomotor defects in mutant fish based on over eight parameters in three basic classes of bout types: slow forward swims, routine turn and fast AV escapes. By distinguishing forward swims from routine turns, we found specific effects of Somatostatin 1.1 on forward swims. A recent analysis of bout types refined their classification into thirteen different categories in zebrafish larvae^48^, including two different subclasses of forward swims. Future studies will tell whether Somatostatin 1.1 is involved in the refinement of other bout types.

In mammals, the locomotor effects of somatostatin reported in the studies mentioned above are generally attributed to the action of somatostatinergic neurons located in the striatum^37,55,59^. However, the action of Somatostatin 1.1 revealed in the present study likely has a different origin since it was not found in the zebrafish homologous region of the striatum at early larval stages^60^. To date, the main expression sites of *somatostatin 1.1* in the 5-dpf zebrafish brain were reported in the vagal motor nucleus^60^, the hypothalamus^61^, the preoptic area^62,63^ and the habenula^64^. In view of the literature available, these last two regions have particularly drawn our attention because they both have a potential link with locomotion.

The *sst1.1*-expressing neurons in the habenula are located in its dorsal part and project to the interpeduncular nucleus^64^. The habenulo-interpeduncular pathway has recently been shown to play an important role in CO_2_ avoidance behavior exhibited by zebrafish larvae^65^. However, whether this pathway is recruited in innate locomotion and whether Somatostatin 1.1 participates in this process remain to be established. Furthermore, *somatostatin 1.1*-expressing neurons of the preoptic area, a subpopulation of orthopedia-expressing neurons, have been recently shown to be involved in the light-search behavior of zebrafish larvae^62,63,66^. In such context, a plausible action of Somatostatin 1.1 may be to modulate the activity of neurons of the dopaminergic diencephalospinal tract, another subpopulation of the orthopedia-specified domain, because fish larvae lacking *somatostatin 1.1* and *orthopedia* mutants exhibit very similar defects. The dopaminergic diencephalospinal tract represents an evolutionarily conserved neuromodulatory system that is necessary for normal vertebrate locomotor development. As a matter of fact, its ablation perturbates locomotor behavior by reducing the time spent swimming but without changing the bout duration^67,68^. However, such phenotype is different to the one reported in the present study, suggesting that the effect of Somatostatin 1.1 on innate locomotion likely does not involve the dopaminergic diencephalospinal tract.

Another important source of the *somatostatin 1.1* transcript in the zebrafish larva is the spinal cord. Previous studies both in lamprey and zebrafish have shown that somatostatin is expressed in the interoceptive neurons contacting the cerebrospinal fluid (‘CSF-cNs’^42,69,70^). These neurons around the central canal^71–73^ can detect chemical^40,70^ and mechanical cues^41,44,74–76^ from the cerebrospinal fluid and, in turn, modulate locomotion^40,43,44,70,77,78^. In zebrafish, dorsal CSF-cNs that express *sst1.1* have been involved in the modulation of slow locomotion by synapsing onto V0-v interneurons^43^. In the lamprey spinal cord, CSF-cNs have been shown to respond to acidic pH and the application of somatostatin via the SSTR2 receptor reduces burst frequency, suggesting that these cells reduce the rhythm of central pattern generators. Further studies will test whether V0-v interneurons express somatostatin receptors.

Altogether, our study demonstrated the inhibitory role of Somatostatin 1.1 in innate locomotion. As somatostatin contributes to growth and development^79^, we cannot exclude that the effects observed here could be due to developmental defects. Note however that the phenotypical analysis of *sst1.1*^*icm40/icm40*^ larval zebrafish at the embryonic, larval and juvenile/adult stages did not show any morphological defects. Further investigations will be necessary to dissect the mechanisms underlying the depressing effects of this neuropeptide as well as the modulatory circuits involved.

## Methods

### Animal care

All animal experimental procedures were approved by the Institutional Ethics Committee at the Institut du Cerveau (ICM) and the European Communities Council Directive (2010/63/EU) under the animal protocol *APAFIS #16469-2018071217081175* for the housing and experimentation on *Danio rerio*. We used the AB and Tüpfel long fin (TL) strains. Fish were maintained and raised on a 14/10 h light cycle. Water temperature was maintained at 28.5 °C, pH = 7.4 and conductivity at 500 μS. We generated the zebrafish mutant line is *sst1.1*^*icm40*^. All embryos and larvae used for experimentation were younger than 6 dpf and were euthanized in 0.2% tricaine (MS 222, Sandoz, Levallois-Perret, France) after experiment. Embryos were raised at low density (30 embryos per petri dish) in blue water (3 g of Instant Ocean salts and 2 mL of methylene blue at 1% in 10 L of osmosed water) until 2 dpf to minimize the development of parasites, then were transferred in the filtered facility system water whose temperature, pH and osmolarity are systematically controlled as described above.

### Generation of the *sst1.1* mutant line and genotyping

To generate loss of function alleles of the *somatostatin 1.1* gene, we used CRISPR/Cas9-mediated genome editing. Two guide RNAs (CAGTGCGCACTGGATACGCGtgg) and (ACAGGAGCGCCAGTGCGCACtgg) (Integrated DNA Technologie, Coralville, USA) were designed by using online CRISPOR algorithm (https://crispor.tefor.net/). We co-injected the two guide RNAs with the Cas9 protein provided by Dr. Jean-Paul Concordet (MNHN, Paris, France) into 1 cell-stage WT eggs. The editing efficiency was tested after injection at 2-dpf by performing genotyping on 3 pools of 20 embryos. Genomic DNA was extracted in lysis buffer (10 mM Tris pH 8.2 mM EDTA, 0.2% Triton X-100, 200 μg/mL Proteinase K) at 55 °C for 2 h followed by 10 min of deactivation at 99 °C. We amplified a region of genomic DNA surrounding the target site using forward (3′-TCTTTTACTCTGAGACCAAATAAACAC-5′) and reverse (3′-TCTATCACATTACATTAGTCAGACGTG-5′) primers resulting in a 227-bp PCR product. Obtained PCR products were then digested using the Mlu1 restriction enzyme. Typically, WT amplified fragments were cleaved into 52- and 179-bp fragments while mutated ones were left intact due to the loss of the restriction site. The amplifications from heterozygous mutants contain both cleaved and intact fragments. The *sst1.1*^*icm40*^ allele was identified and isolated from F1 adults by subcloning and sequencing the target site. The F1 carrier fish were selected and crossed to AB WT fish in order to generate the next generation.

### Behavior recording and analysis

Behavior recordings were blindly conducted on 5-dpf larvae obtained from the incrosses of *sst1.1*^*icm40/*+^ mutants prior to genotyping after euthanasia of all larvae. All behavioral experiments were performed with the third generation of mutant larvae. For each experiment, larvae from one clutch obtained by mating only one male and one female were collected, and the variations between clutches were accounted for in our statistical analysis. Data from heterozygous mutant larvae are available upon request.

### High-throughput behavior assays

To increase the number of fish recording simultaneously, we designed and combined multi-well plates containing 56 wells (127 mm × 140 mm) and 32 wells (80 mm × 140 mm) made from 3 mm acrylic sheets (plexiglass) plate (BFP CINDAR, Champigny-sur-Marne, France) cut by a laser cutter into 1.5 cm diameter wells holding a volume of 500 μL. The well depth was 3 mm to reduce shadow artifacts and the dark blue color was chosen for the plate to increase contrast between larvae and background. The bottom was sealed with a transparent glass coverslip glued to the pre-cut plate using a mix solution of sylgard 184 silicone elastomer base and curing agent (10:2) (Dowcorning, Midland Michigan, USA) to prevent the release of any toxic product. Larvae were illuminated from below with LED plate (R&D vision, Nogent-sur-Marne, France) with a polarized optical filter (ref. 7290110152161, Amazon, France) to provide attenuated and homogeneous light intensity. Prior to each experiment, larvae were transferred individually to the multiwall plates containing facility system water pre-warmed to 28 °C. Abnormal swimming behavior and injured animals were discarded. All larvae were acclimated for 10 min prior to recording. Images were recorded from above with two high performance Cameras: one has focus area up to 2,000 × 1,088 pixels (ref. acA2000-180 km, maximal acquisition rate 734 Hz, Basler, Ahrensburg, Germany) and another one has focus area up to 2,000 × 2,024 pixels (ref. acA2000-340 km, maximal acquisition rate 734 Hz, Basler, Ahrensburg, Germany). Each camera was connected to a computer with the video recording software Hiris ensuring maximal streaming speed (R&D vision, Nogent-sur-Marne, France) (Fig. 1c, see Supplementary Video S1).

We developed high-throughput behavior assays dedicated to the fine kinematic analysis of mutant zebrafish larvae. In our setup, we can simultaneously record 32 and up to 88 zebrafish larvae over minutes-hours at high frequency. We recorded typically at 100 Hz for monitoring spontaneous (slow) locomotion when tail beat frequency occurs below 30 Hz and at 650 Hz for acousto-vestibular escape responses for which tail beat frequency typically reaches 100 Hz at most. Each larva is isolated for the behavioral recording in order to be genotyped afterwards. We slightly reduced the spatial resolution compared to previous conditions^41,49,53^. Due to the larger number of image files to process in parallel, we improved the speed and accuracy of our open source software ZebraZoom by reducing the occurrence of tracking errors in this low resolution low contrast setup from 30% to 0.2% (software available as open source, check https://zebrazoom.org/). To speed up processing by 40 folds, we ran our tracking software on a High Performance Computing (HPC) system that allows tracking hundreds of videos simultaneously. By doing so, ZebraZoom typically took 7 h instead of 258 h to process six 10 min-long videos recorded at 100 Hz. We automated the process for managing big dataset and the file storage to be immediately accessible by the HPC system.

### Escape behavior setup

Larvae were placed individually in a 32-well plate on a transparent plexiglass plate where 2 speakers (Monacor, 10 W, Bremen, Germany) were held on. A custom-made Arduino Due software and circuit was used to synchronize the acoustic stimulus and the recording (Arduino script available on Github (https://github.com/FengBQuan/Quan_et_al_2020.git). To induce an acousto-vestibular (AV) stimulus, we delivered a 5 ms-long sine wave at 500 Hz through a 20 W amplifier (Adafruit, MAX9744, New York, USA) over two speakers at the volume of 1,305 dB. The stimulus occurred 200 ms after the beginning of the 1 s-long trial recorded at 650 Hz. Each larva was subjected to ten stimuli, with 3 min inter-trial interval to minimize habituation (Fig. 1c, see Supplementary Video S1).

### Tracking system

The kinematics of zebrafish larvae were analyzed by performing tracking of the head position, head direction and 10 points along the tail using an improved version of the ZebraZoom software^49,53,77^. First, we improved the source code of ZebraZoom to allow flexibility in the recording conditions depending on the well configuration, the frequency of recording, among other parameters. Visual inspection of the tracking added to the raw video was performed to check that the position and movement was correctly estimated. If a tracking error occurred, if a micro-movement occurred, some of the detection points along the larval body were inaccurate, or the heading direction was wrong, tracking failure was detected and systematically corrected by changing the configuration parameters. Second, ZebraZoom was initially available only on Windows operating system (OS), we adapted the source code to be executable on High Performance Computing (HPC) systems, which use Linux OS. Many videos were larger than several hundred gigabytes. We automated the management of large dataset from acquisition to the shared storage (Lustre) on ICM network, allowing the dataset to be immediately accessible by the ICM HPC system. The pipeline of ZebraZoom has been adapted to be used by multiple processors on a HPC system in order to simultaneously track numerous videos. The ICM HPC system is composed of 33 servers for a total of 924 cores and 5,623 gigabytes of memory. It is connected to the Lustre file system (https://lustre.org) of 2.9 petabytes (Fig. 1c, see Supplementary Video S1).

### Analysis of kinematic parameters

From the heading and the tail position tracked by ZebraZoom (https://zebrazoom.org/), we extracted the timing of bouts and kinematic parameters per bout derived from the tail angle: bout duration, distance travelled, number of oscillations, amplitudes of each tail bend. Custom-made script packages (Matlab R2018b, The MathWorks Inc., Natick, MA, USA) were used for analysis: SlowSwim Analysis and Escape Analysis (https://github.com/FengBQuan/Quan_et_al_2020.git). For the analysis of spontaneous slow locomotion, larval zebrafish that swam less than 30 bouts over the 5 min-long recording were discarded. For all other cases, we calculated the median value of each kinematic parameter across all bouts. For the analysis of AV escape responses, we selected the first bout occurring following the stimuli with a latency below 30 ms and the first tail bend above 60°. Kinematic parameters were averaged per fish across the ten trials.

### Statistical analysis

All statistical analyses for the escape and the slow swim data were conducted by using the R software^80^ (version 3.6.1, https://cran.rproject.org/). Values were presented as mean ± standard error of the mean (SEM). The comparisons between the two genotype groups were performed by using linear mixed models (LMMs) with a fixed effect for genotype and a random effect for the clutch. All LMMs were fitted to the kinematic parameters using the function lmer in the lme4 R package. When necessary, the data were either log or square root transformed prior to the modeling to improve the model assumptions of linearity, normality and constant variance of residuals. Therefore, a significant difference between the two genotype groups was evaluated with the Anova function in the car package using a Type II Wald chi-square test. One fish (N°204) exhibited an abnormal morphology revealing an injury possibly occurring during pipetting and the subsequent data was removed from analysis. In order to perform multiple comparisons, the correlation between all kinematic parameters was evaluated for each analysis. Following a method previously described^81^, the value of Meff was calculated for each analysis as 1 + (k − 1) × (1 − vcorr/k), with k denoting the total number of non-independent tests and vcorr denoting the variance of eigenvalues from the correlation matrix of the kinematic parameters based on Spearman’s rank correlation. All p-values from the Type II Wald chi-square tests were multiplied by a number of effective independent tests (Meff) for adjustment. The level of statistical significance was set at p < 0.05 for all tests.

## Supplementary information

Supplementary Information 1.

Supplementary Information 2.

Supplementary Information 3.

Supplementary Video 1.
