# Supplementary material for: Somatostatin 1.1 contributes to the innate exploration of zebrafish larva
Source: Sci Rep. 2020 Sep 17;10:15235. doi: 10.1038/s41598-020-72039-x (PMC7499426; doi:10.1038/s41598-020-72039-x)
Supplement: Supplementary file 1 — Supplementary Information 1. [file 41598_2020_72039_MOESM1_ESM.pdf]

Escape analysis

FX Lejeune - iCONICS

2020-04-18

Table of Contents

Import data from files 4
Method 5
Analysis using one average value per fish 6
Data visualization 6
Correlation matrix 7
Cbend [log, ns] 8
Fitting LMM 8
Checking model assumptions 9
Conclusion 9
Counterbend [sqrt, ns] 10
Fitting LMM 10
Checking model assumptions 11
Conclusion 11
Distance [log, ns] 12
Fitting LMM 12
Checking model assumptions 13
Conclusion 13
Duration [ns] 14
Fitting LMM 14
Checking model assumptions 15
Conclusion 15
Latency [sqrt, ns] 16
Fitting LMM 16
Checking model assumptions 17
Conclusion 17
mTBF [ns] 18
Fitting LMM 18

Checking model assumptions....19
Conclusion....19
Number of oscillations [sqrt, ns]....20
Fitting LMM....20
Checking model assumptions....21
Conclusion....21
Speed [ns]....22
Fitting LMM....22
Checking model assumptions....23
Conclusion....23
Analysis across the 10 trials....24
Data visualization....24
Data visualization with clutch info....25
Correlation matrix....26
Cbend [ns]....27
Fitting LMM....27
Group comparison at each trial....28
Checking model assumptions....30
Conclusion....30
Counterbend [ns]....31
Fitting LMM....31
Group comparison at each trial....32
Checking model assumptions....34
Conclusion....34
Distance [ns]....35
Fitting LMM....35
Group comparison at each trial....36
Checking model assumptions....38
Conclusion....38
Duration [sqrt, ns]....39
Fitting LMM....39
Group comparison at each trial....40
Checking model assumptions....42
Conclusion....42

Latency [ns] 43
Fitting LMM 43
Group comparison at each trial 44
Checking model assumptions 46
Conclusion 46
mTBF [ns] 47
Fitting LMM 47
Group comparison at each trial 48
Checking model assumptions 50
Conclusion 50
Number of oscillations [ns] 51
Fitting LMM 51
Group comparison at each trial 52
Checking model assumptions 54
Conclusion 54
Speed [ns] 55
Fitting LMM 55
Group comparison at each trial 56
Checking model validity 58
Conclusion 58
R session information 59

Statistical analysis for Feng Quan and Claire Wyart

Import data from files

8 kinematic parameters were measured at 10 trials in the slow and fast swim gait: Cbend, Counterbend, Distance, Duration, Latency, mTBF, NumOfOsc and Speed.

Number of values used by Genotype and Clutch

	Cbend	Counterbend	Distance	Duration	Latency	mTBF	NumOfOsc	Speed
WT_2	18	18	18	18	18	18	18	18
WT_3	20	20	20	20	20	20	20	20
WT_4	28	28	28	28	28	28	28	28
WT_5	25	25	25	25	25	25	25	25
Hom_2	17	17	17	17	17	17	17	17
Hom_3	23	23	23	23	23	23	23	23
Hom_4	14	14	14	14	14	14	14	14
Hom_5	26	26	26	26	26	26	26	26

Mean values by Genotype and Clutch

	WT_2	WT_3	WT_4	WT_5	Hom_2	Hom_3	Hom_4	Hom_5
Cbend	103.4	102.5	101.3	92.3	104.9	101.6	102.7	91.5
Counterbend	45.6	46.6	46.2	36.5	42.9	49.2	51.8	40.9
Distance	8.6	9.6	9.1	8.3	9.1	10.3	9.2	8.0
Duration	0.3	0.3	0.2	0.3	0.3	0.3	0.3	0.3
Latency	6.2	5.0	5.2	3.6	6.6	5.2	5.8	4.0
mTBF	34.8	36.9	37.2	43.6	31.9	37.9	37.2	44.7
NumOfOsc	8.0	9.7	7.5	8.8	7.7	8.5	7.9	8.8
Speed	33.5	34.5	38.1	34.9	33.4	39.3	37.4	34.0

Method

All statistical analyses were conducted using R version 3.6.1 (https://www.R-project.org/), and plots were generated with the ggplot2 package.

Between-group comparison based on trial-averaged values was conducted through a linear mixed model using the lmer function in the lme4 package (for multiple-day data collection). Mean difference between the two groups (Hom and WT) was then tested using the emmeans package with the Tukey's method for multiplicity adjustment. To better match the model assumptions (normality and homoscedasticity of residuals), data were transformed by the natural logarithm or the square root function prior to modeling.

Between-group comparison across the ten trials was performed using a linear mixed model (function lmer in the lme4 package) with the fixed effects of Genotype and Trial, and the random effect of Fish_ID nested within Clutch for repeated measures. Significance for the main effects of genotype (WT vs. Homozygotous mutants), Trial and their interaction was then evaluated using the Anova function in the car package with Type II Wald chi-square tests. To improve normality and homoscedasticity of residuals, data were transformed by the natural logarithm or the square root function prior to modeling.

For each kinematic parameter, the estimated marginal means and model-based standard errors on the original scale (non-transformed data) were extracted for each genotype using the emmeans package.

The level of statistical significance was set at $p < 0.05$ for all tests.

Analysis using one average value per fish

Data visualization

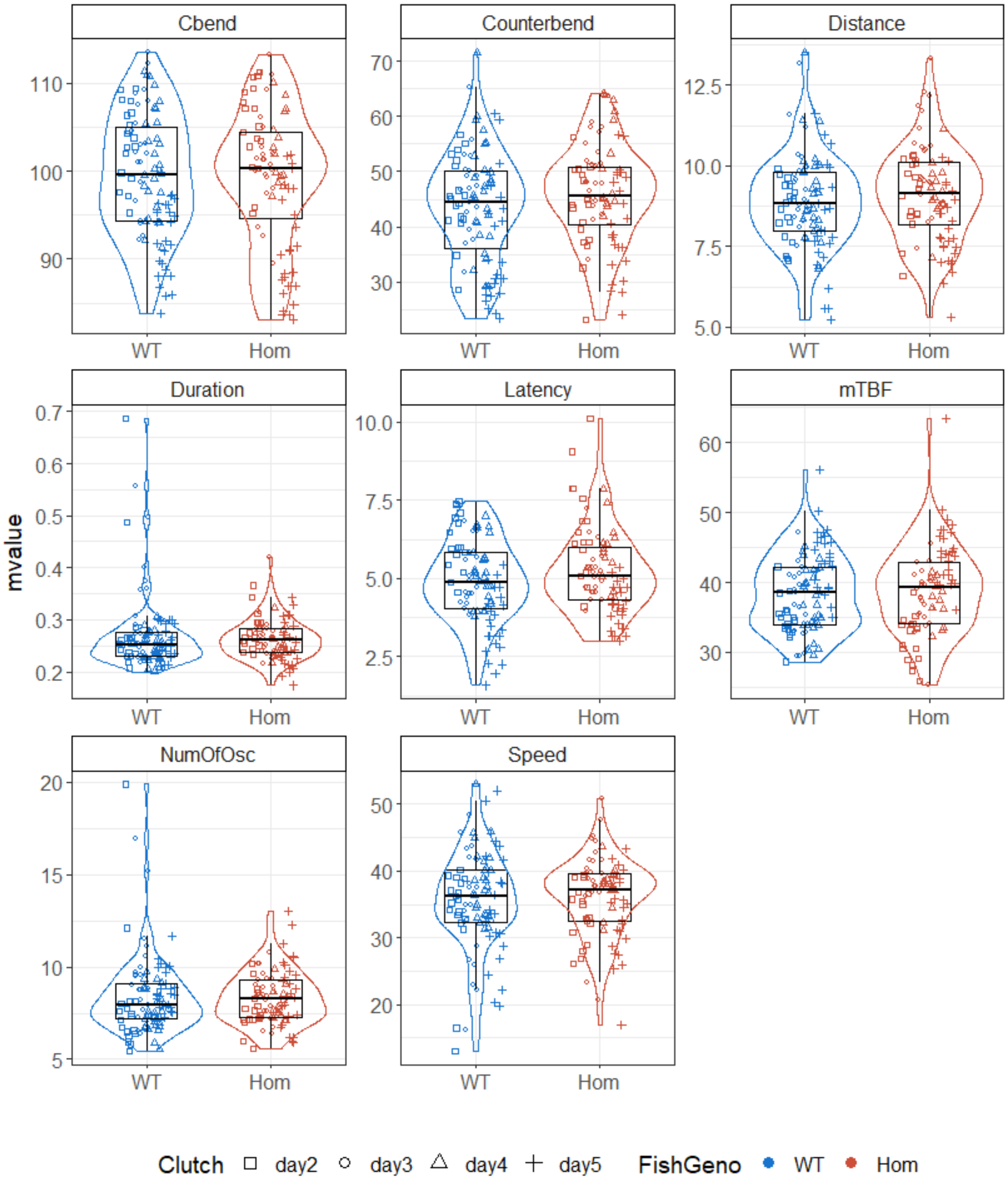

Correlation matrix

Using Spearman's rank correlations on average values:

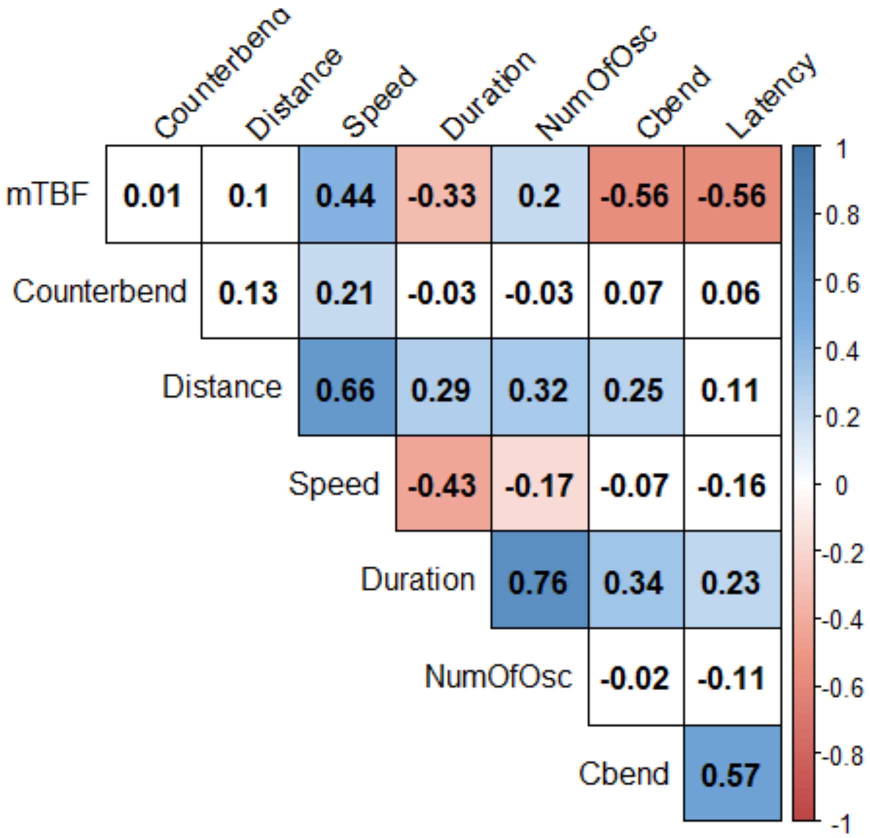

Based on 8 kinematic parameters, variance of eigenvalues of the correlation matrix is 0.879 leading to an effective number of independent tests (Meff) of 7.230 to be used for multiple testing correction. Method described in Nyholt DR, A simple correction for multiple testing for single-nucleotide polymorphisms in linkage disequilibrium with each other, Am J Hum Genet, 2004 [PMID: 14997420].

Cbend [log, ns]

Fitting LMM

```
## Linear mixed model fit by REML ['lmerMod']
## Formula: log(mvalue) ~ FishGeno + (1 | Clutch)
##    Data: tmp
## REML criterion at convergence: -481.027
## Random effects:
##  Groups   Name        Std.Dev.
##  Clutch   (Intercept) 0.05604
##  Residual             0.05491
## Number of obs: 171, groups:  Clutch, 4
## Fixed Effects:
## (Intercept)  FishGenoHom
##    4.601577     0.001289
```

```
## Analysis of Deviance Table (Type II Wald chisquare tests)
##
## Response: log(mvalue)
##           Chisq Df Pr(>Chisq)
## FishGeno 0.0229  1     0.8797
```

Estimated marginal means with model-based standard errors and 95% confidence limits on the original scale (non-transformed data):

```
## 'emmGrid' object with variables:
##     FishGeno = WT, Hom
## Transformation: "log"
```

```
##  FishGeno response   SE   df lower.CL upper.CL
##  WT           99.6 2.85 3.12     91.1      109
##  Hom          99.8 2.86 3.16     91.3      109
##
## Degrees-of-freedom method: kenward-roger
## Confidence level used: 0.95
## Intervals are back-transformed from the log scale
```

Checking model assumptions

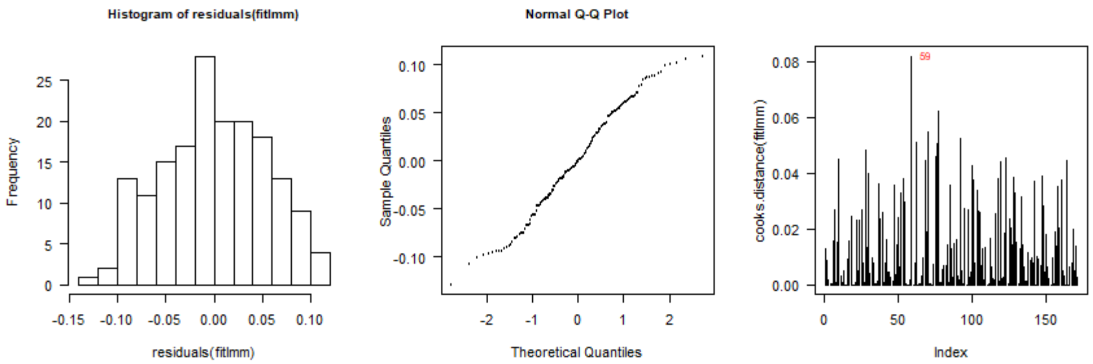

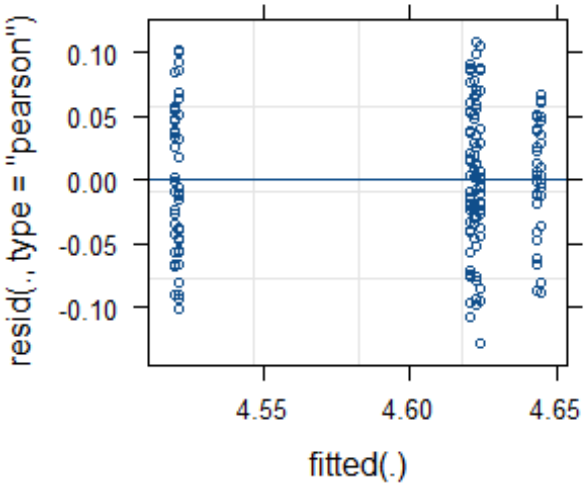

Conclusion

No significant difference was detected between the two groups (Type II Wald chi-square test; ^{ns}p = 0.880). Adj. Pvalue: ^{ns}p = 1 (using Meff = 7.230 tests).

Counterbend [sqrt, ns]

Fitting LMM

```
## Linear mixed model fit by REML ['lmerMod']
## Formula: sqrt(mvalue) ~ FishGeno + (1 | Clutch)
##    Data: tmp
## REML criterion at convergence: 360.0286
## Random effects:
##  Groups   Name        Std.Dev.
##  Clutch   (Intercept) 0.3431
##  Residual             0.6689
## Number of obs: 171, groups:  Clutch, 4
## Fixed Effects:
## (Intercept)  FishGenoHom
##      6.5521       0.2117
```

```
## Analysis of Deviance Table (Type II Wald chisquare tests)
##
## Response: sqrt(mvalue)
##           Chisq Df Pr(>Chisq)
## FishGeno 4.1674  1    0.04121 *
## ---
## Signif. codes:  0 '***' 0.001 '**' 0.01 '*' 0.05 '.' 0.1 ' ' 1
```

Estimated marginal means with model-based standard errors and 95% confidence limits on the original scale (non-transformed data):

```
## 'emmGrid' object with variables:
##     FishGeno = WT, Hom
## Transformation: "sqrt"
```

```
##  FishGeno response   SE   df lower.CL upper.CL
##  WT           42.9 2.43 3.45     36.0     50.4
##  Hom          45.7 2.54 3.59     38.7     53.4
##
## Degrees-of-freedom method: kenward-roger
## Confidence level used: 0.95
## Intervals are back-transformed from the sqrt scale
```

Checking model assumptions

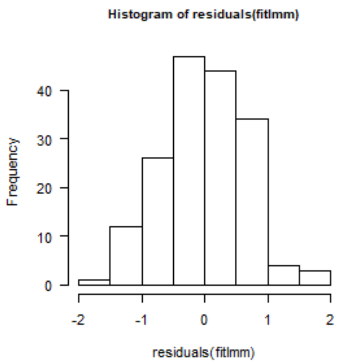

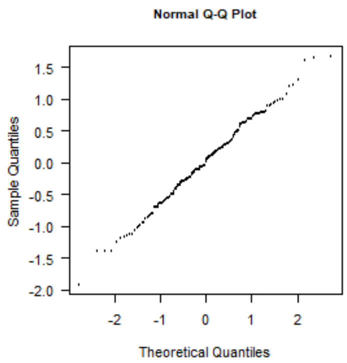

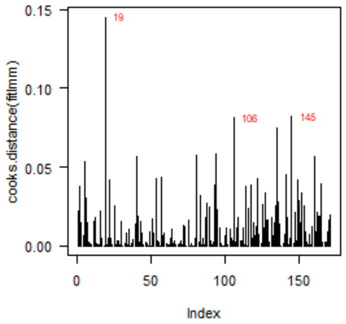

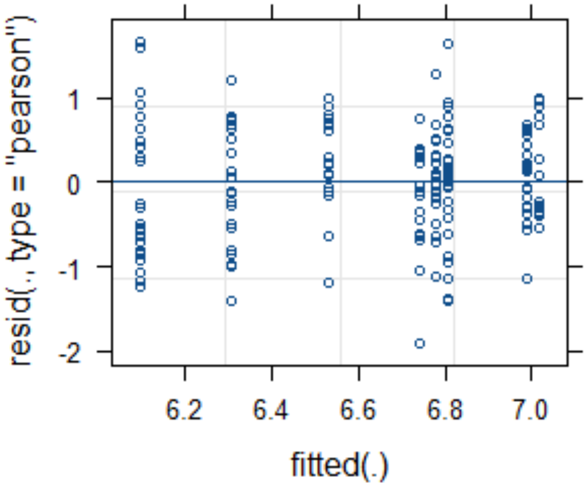

Conclusion

Linear mixed model indicated a significant difference between the two groups (Type II Wald chi-square test; *p = 0.041). Adj. Pvalue: ^{ns}p = 0.298 (using Meff = 7.230 tests). Unadjusted p-value remains significant after removing the most influential data point 19 (see Cook's distance).

Distance [log, ns]

The residuals distribution is skewed without the log transformations.

Fitting LMM

```
## Linear mixed model fit by REML ['lmerMod']
## Formula: log(mvalue) ~ FishGeno + (1 | Clutch)
##    Data: tmp
## REML criterion at convergence: -146.0768
## Random effects:
##  Groups   Name        Std.Dev.
##  Clutch   (Intercept) 0.0846
##  Residual             0.1494
## Number of obs: 171, groups:  Clutch, 4
## Fixed Effects:
## (Intercept)  FishGenoHom
##     2.17487      0.02371
```

```
## Analysis of Deviance Table (Type II Wald chisquare tests)
##
## Response: log(mvalue)
##           Chisq Df Pr(>Chisq)
## FishGeno 1.0477  1      0.306
```

Estimated marginal means with model-based standard errors and 95% confidence limits on the original scale (non-transformed data):

```
## 'emmGrid' object with variables:
##     FishGeno = WT, Hom
## Transformation: "log"
```

```
##  FishGeno response    SE   df lower.CL upper.CL
##  WT           8.80 0.397 3.37     7.69     10.1
##  Hom          9.01 0.411 3.49     7.88     10.3
##
## Degrees-of-freedom method: kenward-roger
## Confidence level used: 0.95
## Intervals are back-transformed from the log scale
```

Checking model assumptions

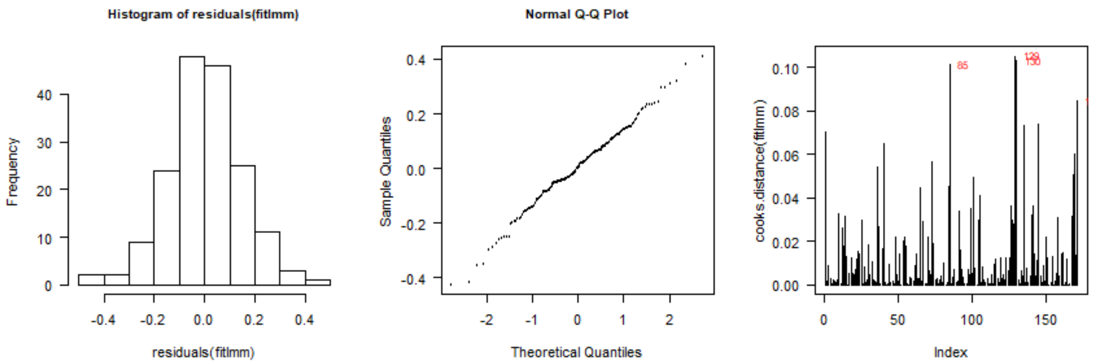

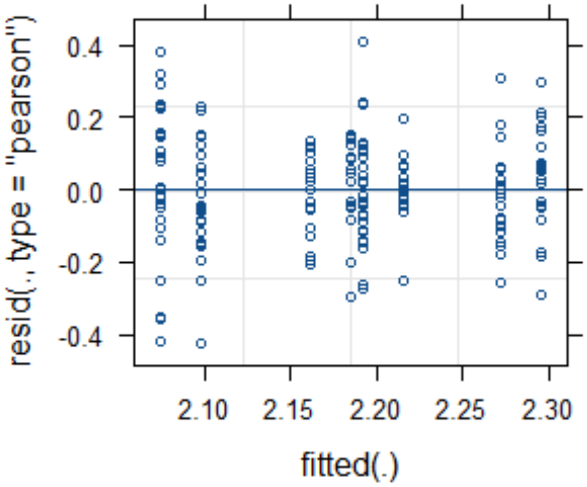

Conclusion

No significant difference was detected between the two groups (Type II Wald chi-square test; $^{ns}p = 0.306$). Adj. Pvalue: $^{ns}p = 1$ (for Meff = 7.230 tests).

Duration [ns]

Fitting LMM

```
## Linear mixed model fit by REML ['lmerMod']
## Formula: mvalue ~ FishGeno + (1 | Clutch)
##    Data: tmp
## REML criterion at convergence: -468.2959
## Random effects:
##  Groups   Name        Std.Dev.
##  Clutch   (Intercept) 0.01968
##  Residual             0.05805
## Number of obs: 171, groups:  Clutch, 4
## Fixed Effects:
## (Intercept)  FishGenoHom
##    0.271820    -0.008976
```

```
## Analysis of Deviance Table (Type II Wald chisquare tests)
##
## Response: mvalue
##           Chisq Df Pr(>Chisq)
## FishGeno 0.9967  1     0.3181
```

Estimated marginal means with model-based standard errors and 95% confidence limits on the original scale (non-transformed data):

```
## 'emmGrid' object with variables:
##     FishGeno = WT, Hom
```

```
##  FishGeno emmean     SE   df lower.CL upper.CL
##  WT        0.272 0.0116 3.95    0.239    0.304
##  Hom       0.263 0.0118 4.27    0.231    0.295
##
## Degrees-of-freedom method: kenward-roger
## Confidence level used: 0.95
```

Checking model assumptions

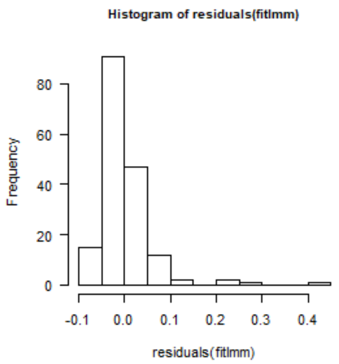

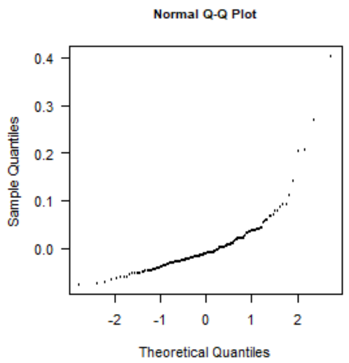

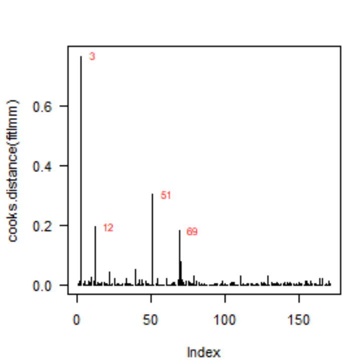

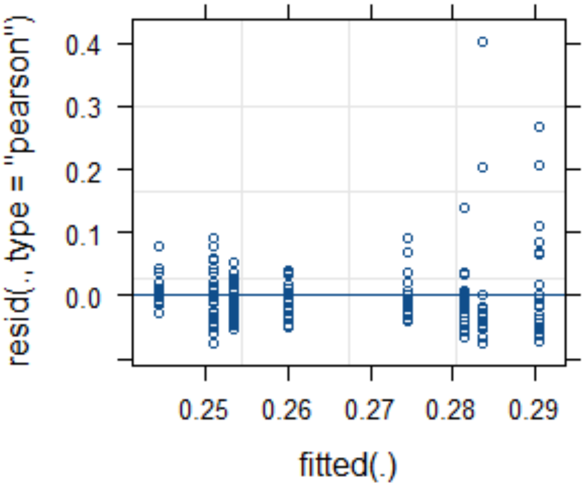

Conclusion

Bad model fit. There does not seem to be any significant effect of the group (Type II Wald chi-square test; $^{ns}p = 0.318$). Adj. Pvalue: $^{ns}p = 1$ (for Meff = 7.230 tests).

Latency [sqrt, ns]

Skewed distribution of residuals without square root transformation.

Fitting LMM

```
## Linear mixed model fit by REML ['lmerMod']
## Formula: sqrt(mvalue) ~ FishGeno + (1 | Clutch)
##    Data: tmp
## REML criterion at convergence: -15.6549
## Random effects:
##  Groups   Name        Std.Dev.
##  Clutch   (Intercept) 0.2353
##  Residual             0.2173
## Number of obs: 171, groups:  Clutch, 4
## Fixed Effects:
## (Intercept)  FishGenoHom
##     2.21005      0.09109
```

```
## Analysis of Deviance Table (Type II Wald chisquare tests)
##
## Response: sqrt(mvalue)
##          Chisq Df Pr(>Chisq)
## FishGeno 7.297  1   0.006907 **
## ---
## Signif. codes:  0 '***' 0.001 '**' 0.01 '*' 0.05 '.' 0.1 ' ' 1
```

Estimated marginal means with model-based standard errors and 95% confidence limits on the original scale (non-transformed data):

```
## 'emmGrid' object with variables:
##     FishGeno = WT, Hom
## Transformation: "sqrt"
```

```
##  FishGeno response    SE   df lower.CL upper.CL
##  WT           4.88 0.530 3.10     3.37     6.68
##  Hom          5.30 0.553 3.14     3.72     7.15
##
## Degrees-of-freedom method: kenward-roger
## Confidence level used: 0.95
## Intervals are back-transformed from the sqrt scale
```

Checking model assumptions

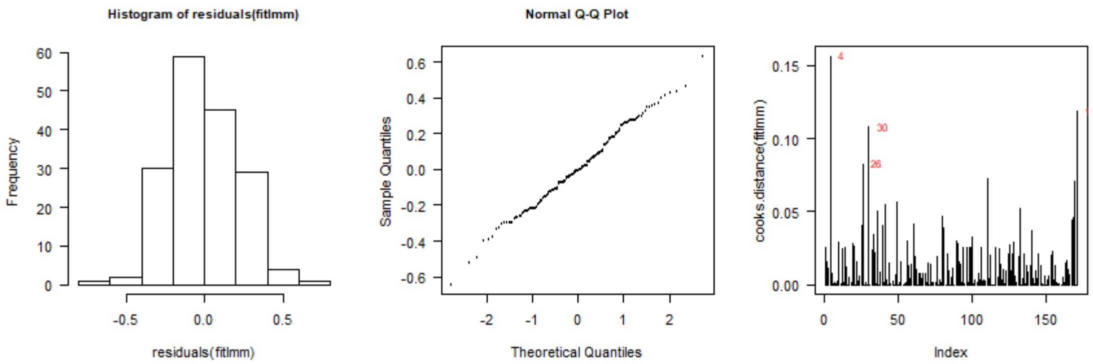

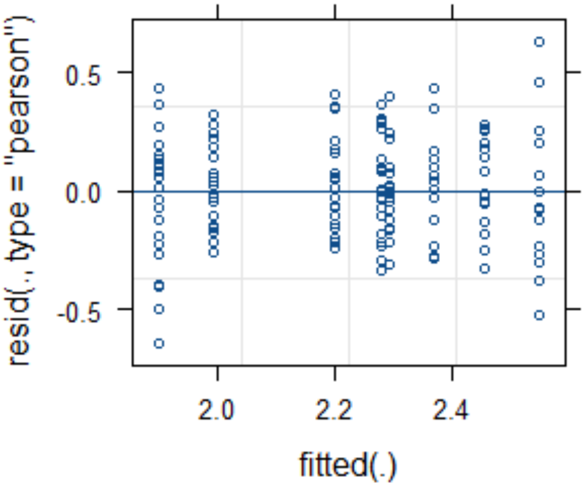

Conclusion

Linear mixed model indicated a significant difference between the two groups (ANOVA Type II test; $^{**}p = 6.9e\text{-}3$). Adj. Pvalue: $^{ns}p = 0.050$ (using Meff = 7.230 tests). However, checking for model assumptions, we did not retain the effect as it was no more present after exclusion of one influential observation (point 4) indicated by the Cook's distance.

mTBF [ns]

Fitting LMM

```
## Linear mixed model fit by REML ['lmerMod']
## Formula: mvalue ~ FishGeno + (1 | Clutch)
##    Data: tmp
## REML criterion at convergence: 998.9557
## Random effects:
##  Groups   Name        Std.Dev.
##  Clutch   (Intercept) 4.439
##  Residual             4.378
## Number of obs: 171, groups:  Clutch, 4
## Fixed Effects:
## (Intercept)  FishGenoHom
##    38.07237     -0.02279
```

```
## Analysis of Deviance Table (Type II Wald chisquare tests)
##
## Response: mvalue
##           Chisq Df Pr(>Chisq)
## FishGeno 0.0011  1     0.9732
```

Estimated marginal means with model-based standard errors and 95% confidence limits on the original scale (non-transformed data):

```
## 'emmGrid' object with variables:
##     FishGeno = WT, Hom
```

```
##  FishGeno emmean   SE   df lower.CL upper.CL
##  WT         38.1 2.27 3.12       31     45.1
##  Hom        38.0 2.27 3.16       31     45.1
##
## Degrees-of-freedom method: kenward-roger
## Confidence level used: 0.95
```

Checking model assumptions

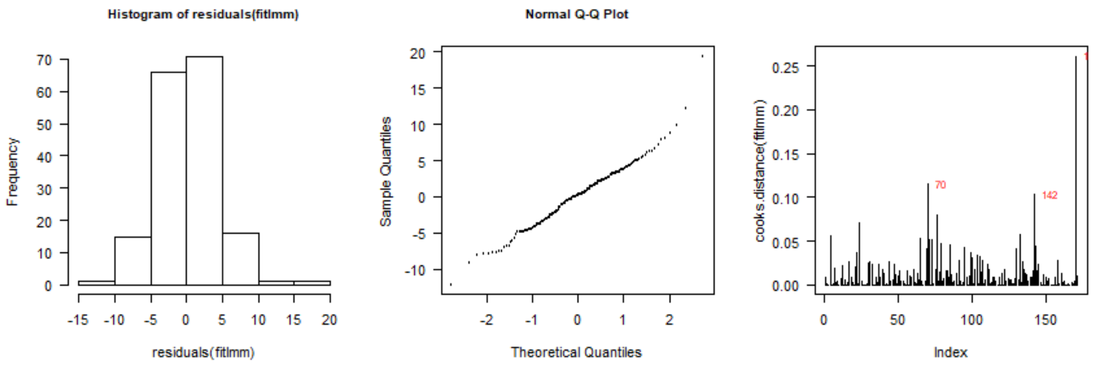

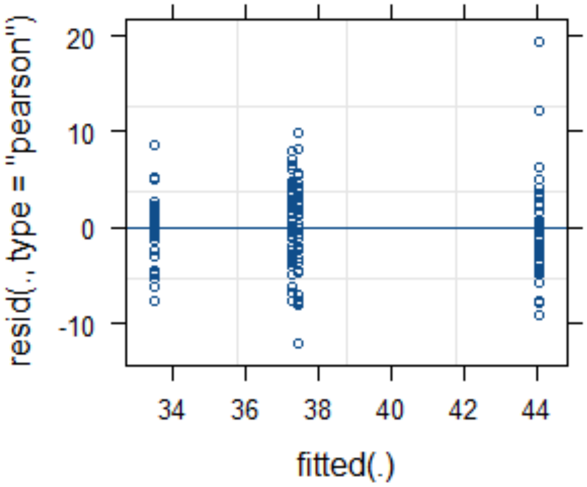

Conclusion

No significant difference was detected between the two groups (Type II Wald chi-square test; ^{ns}p = 0.973). Adj. Pvalue: ^{ns}p = 1 (for Meff = 7.230 tests).

Number of oscillations [sqrt, ns]

Fitting LMM

```
## Linear mixed model fit by REML ['lmerMod']
## Formula: sqrt(mvalue) ~ FishGeno + (1 | Clutch)
##    Data: tmp
## REML criterion at convergence: 71.1761
## Random effects:
##  Groups   Name        Std.Dev.
##  Clutch   (Intercept) 0.1132
##  Residual             0.2857
## Number of obs: 171, groups:  Clutch, 4
## Fixed Effects:
## (Intercept)  FishGenoHom
##     2.88692     -0.03236

## Analysis of Deviance Table (Type II Wald chisquare tests)
##
## Response: sqrt(mvalue)
##           Chisq Df Pr(>Chisq)
## FishGeno 0.5342  1     0.4648
```

Estimated marginal means with model-based standard errors and 95% confidence limits on the original scale (non-transformed data):

```
## 'emmGrid' object with variables:
##     FishGeno = WT, Hom
## Transformation: "sqrt"

##  FishGeno response    SE   df lower.CL upper.CL
##  WT           8.33 0.370 3.72     7.31     9.43
##  Hom          8.15 0.372 3.96     7.14     9.22
##
## Degrees-of-freedom method: kenward-roger
## Confidence level used: 0.95
## Intervals are back-transformed from the sqrt scale
```

Checking model assumptions

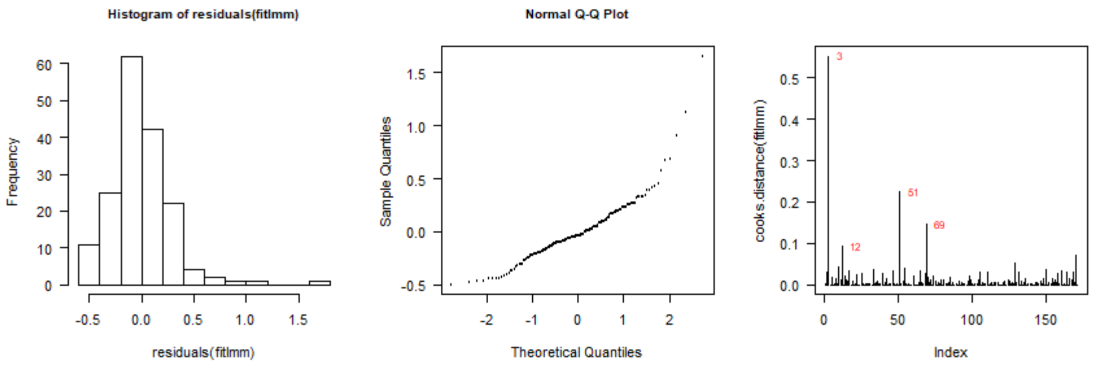

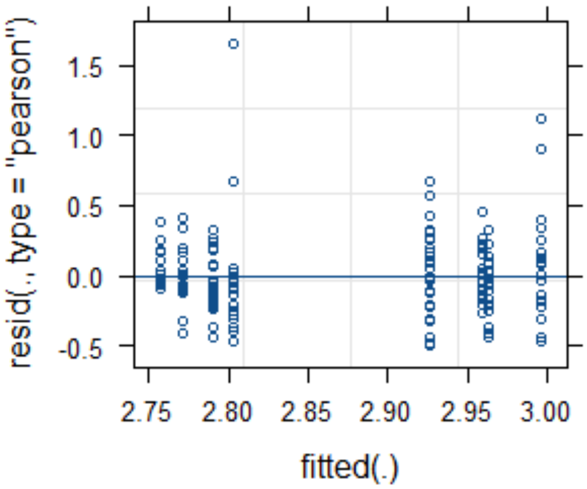

Conclusion

No significant difference was detected between the two groups (Type II Wald chi-square test; ^{ns}p = 0.465). Adj. Pvalue: ^{ns}p = 1 (for Meff = 7.230 tests).

Speed [ns]

Fitting LMM

```
## Linear mixed model fit by REML ['lmerMod']
## Formula: mvalue ~ FishGeno + (1 | Clutch)
##    Data: tmp
## REML criterion at convergence: 1136.087
## Random effects:
##  Groups   Name        Std.Dev.
##  Clutch   (Intercept) 1.822
##  Residual             6.709
## Number of obs: 171, groups:  Clutch, 4
## Fixed Effects:
## (Intercept)  FishGenoHom
##     35.3987       0.7016
```

```
## Analysis of Deviance Table (Type II Wald chisquare tests)
##
## Response: mvalue
##           Chisq Df Pr(>Chisq)
## FishGeno 0.4569  1     0.4991
```

Estimated marginal means with model-based standard errors and 95% confidence limits on the original scale (non-transformed data):

```
## 'emmGrid' object with variables:
##     FishGeno = WT, Hom
```

```
##  FishGeno emmean   SE   df lower.CL upper.CL
##  WT         35.4 1.15 4.38     32.3     38.5
##  Hom        36.1 1.19 4.86     33.0     39.2
##
## Degrees-of-freedom method: kenward-roger
## Confidence level used: 0.95
```

Checking model assumptions

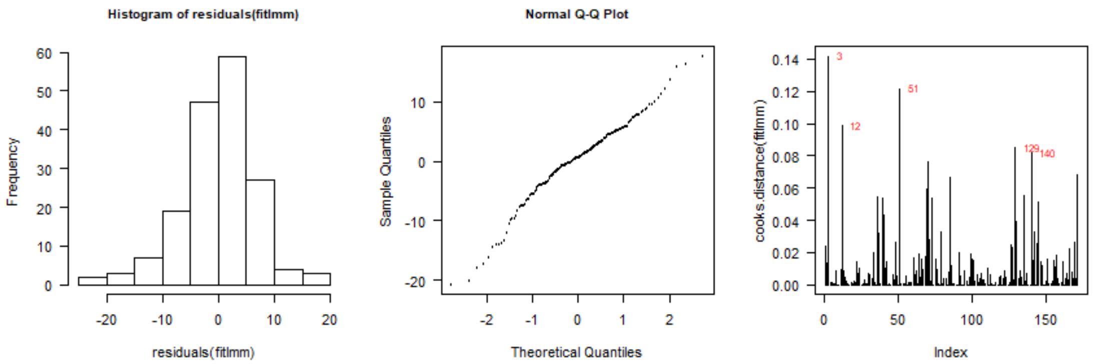

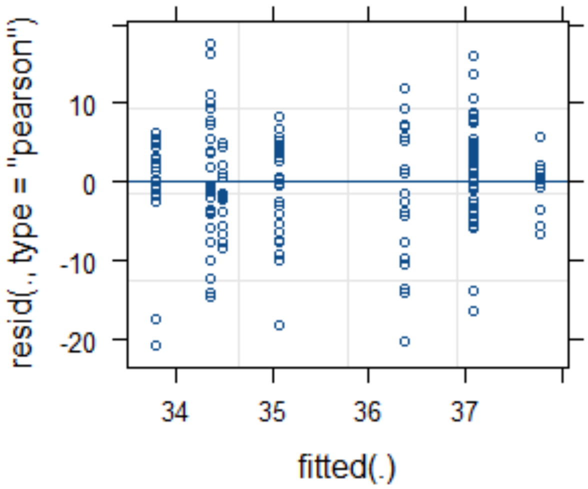

Conclusion

No significant difference was detected between the two groups (Type II Wald chi-square test; ^{ns}p = 0.499). Adj. Pvalue: ^{ns}p = 1 (for Meff = 7.230 tests).

Analysis across the 10 trials

Data visualization

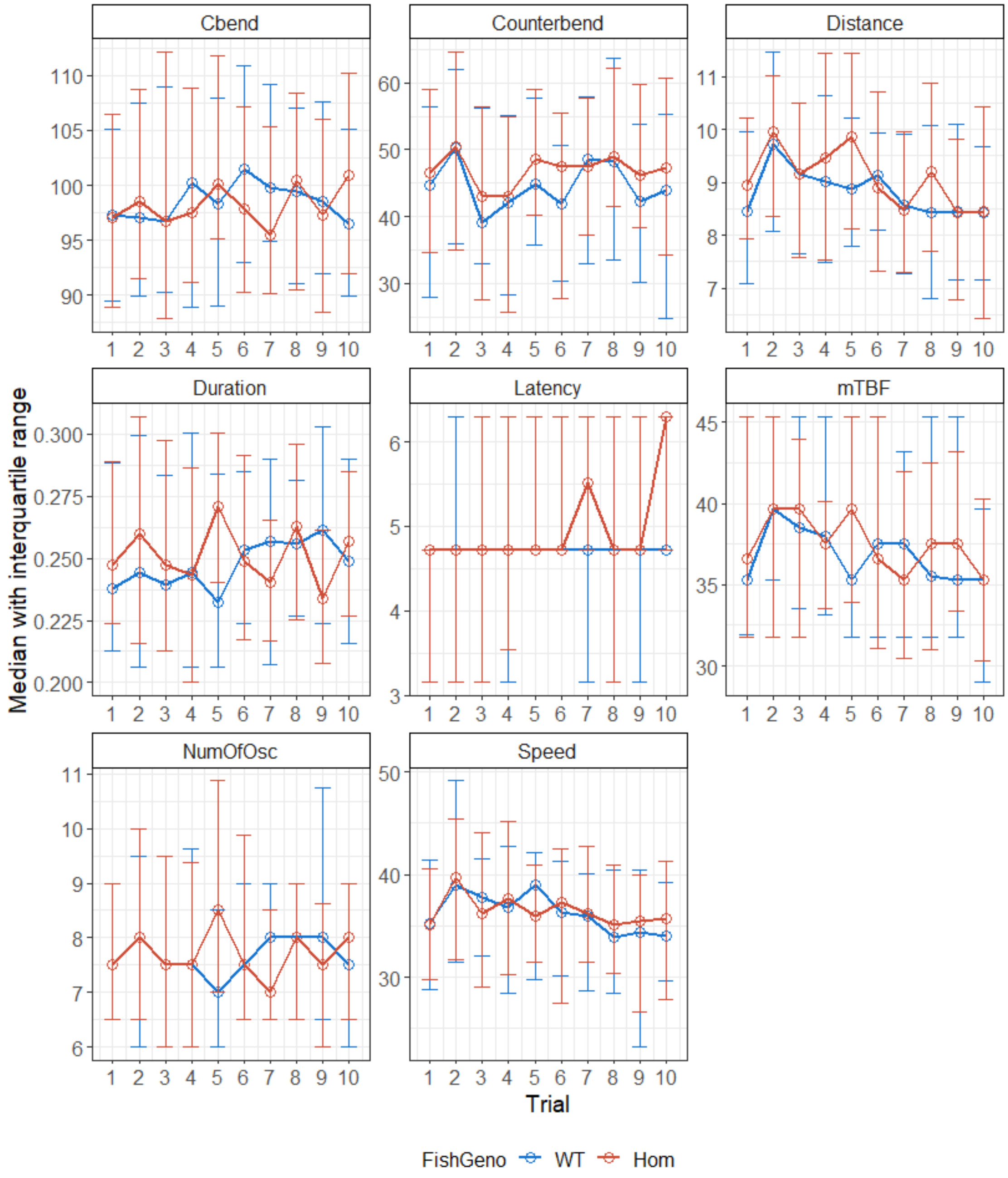

Data visualization with clutch info

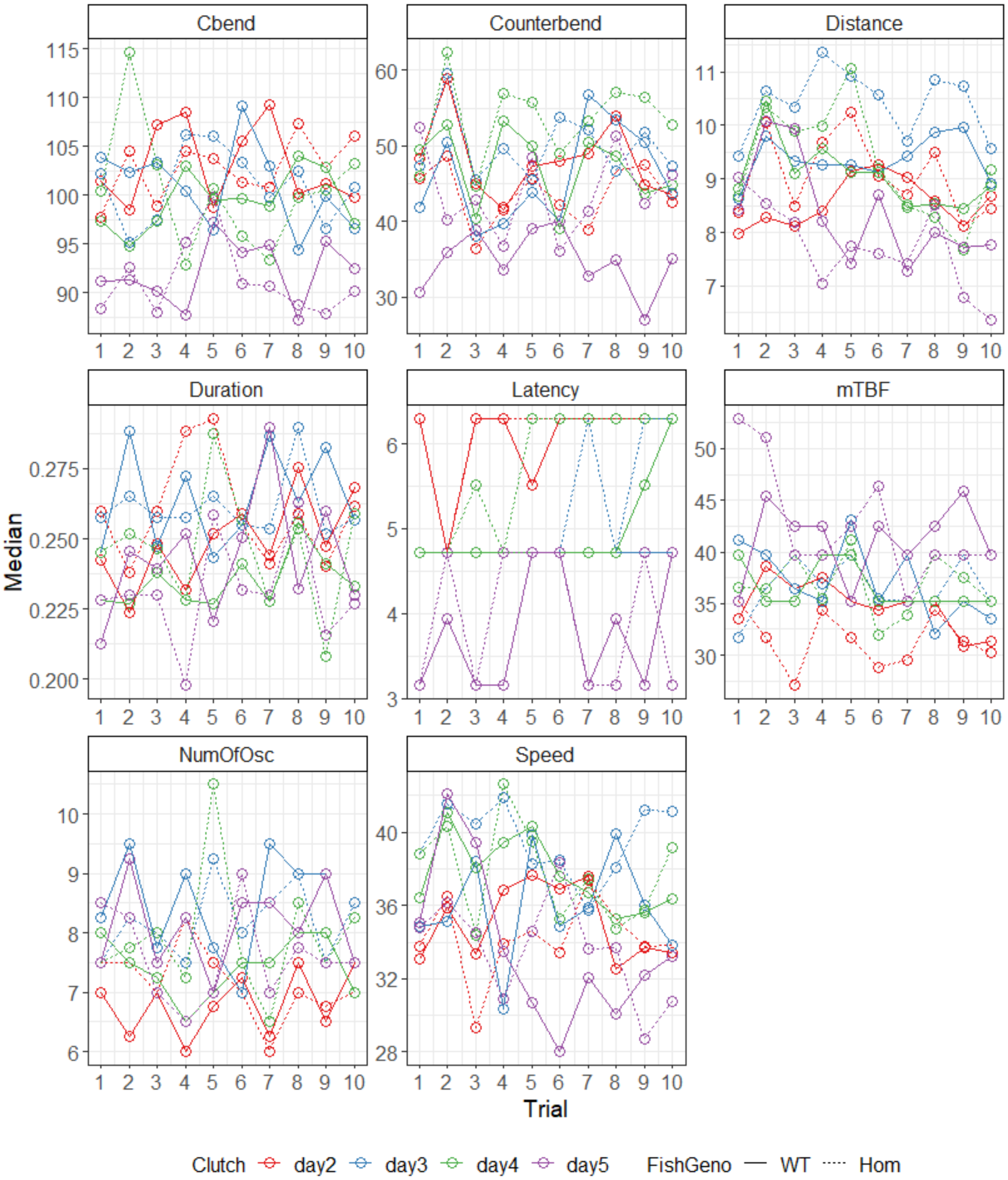

Correlation matrix

Using Spearman's rank correlations and all the trials:

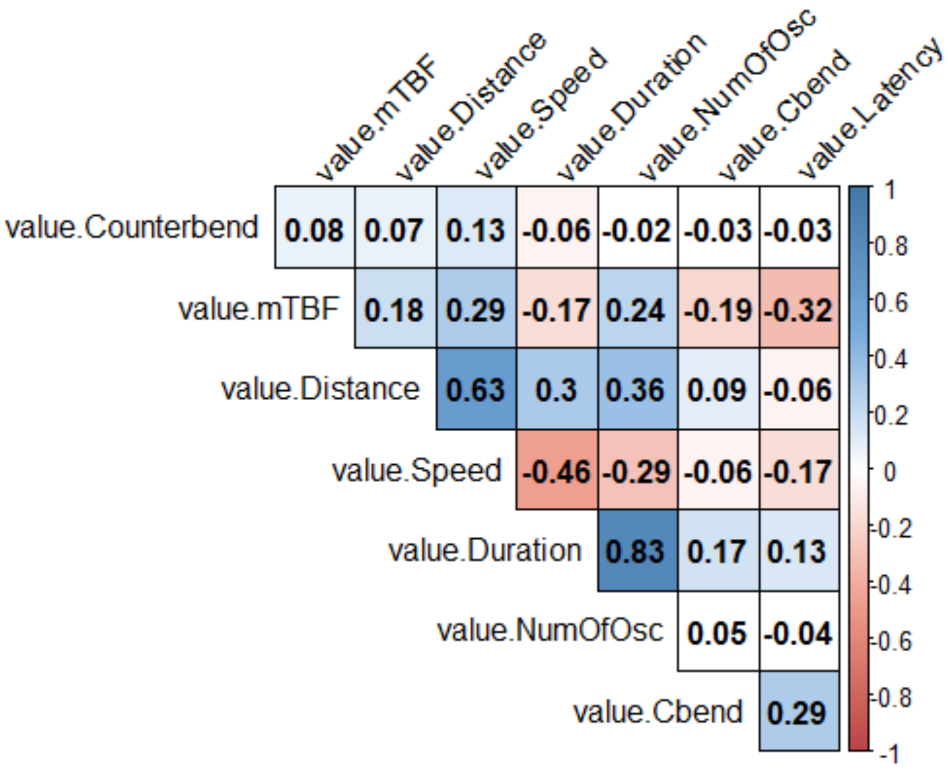

Based on 8 kinetic parameters, variance of eigenvalues of the correlation matrix is 0.622 leading to a Meff value of 7.456 to be used for multiple testing correction. Method described in Nyholt DR, A simple correction for multiple testing for single-nucleotide polymorphisms in linkage disequilibrium with each other, Am J Hum Genet, 2004 [PMID: 14997420].

Cbend [ns]

Fitting LMM

```
## Linear mixed model fit by REML ['lmerMod']
## Formula: value ~ FishGeno * time + (1 | Clutch/Fish)
##    Data: tmp
## REML criterion at convergence: 11973.44
## Random effects:
##  Groups      Name        Std.Dev.
##  Fish:Clutch (Intercept)  3.48
##  Clutch      (Intercept)  5.08
##  Residual                12.88
## Number of obs: 1495, groups:  Fish:Clutch, 147; Clutch, 4
## Fixed Effects:
##     (Intercept)       FishGenoHom              time  FishGenoHom:time
##        99.53922           1.28642           0.08319          -0.16176
```

```
## Analysis of Deviance Table (Type II Wald chisquare tests)
##
## Response: value
##                Chisq Df Pr(>Chisq)
## FishGeno      0.1889  1     0.6638
## time          0.0052  1     0.9428
## FishGeno:time 0.4683  1     0.4938
```

Group comparison at each trial

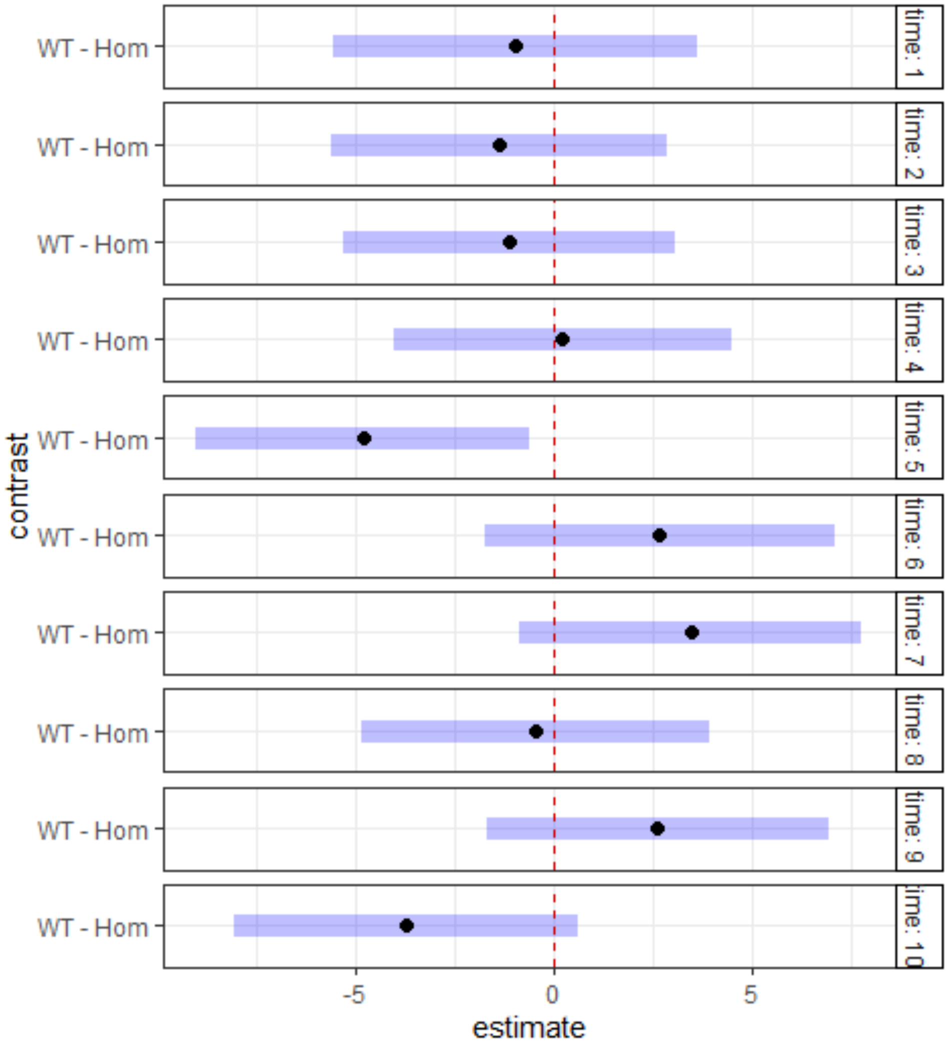


```
##
##
## Table: Post hoc Tukey's: Mean differences between groups at each trial
##
##  contrast    time     estimate        SE          df         t.ratio
p.value
## ----------  ------  ------------  ----------  ----------  ------------  --
---------
##  WT - Hom     1      -0.9461566    2.350722    1384.246    -0.4024961
0.6873811
##  WT - Hom     2      -1.3603148    2.164448    1349.969    -0.6284811
0.5297951
##  WT - Hom     3      -1.0948662    2.133491    1339.268    -0.5131806
0.6079096
##  WT - Hom     4      0.2279092     2.175144    1356.868    0.1047789
0.9165667
```

```
##  WT - Hom      5       -4.7921306     2.143612    1348.704    -2.2355398
0.0255450
##  WT - Hom      6       2.6865312      2.253672    1373.360    1.1920681
0.2334405
##  WT - Hom      7       3.4692846      2.199193    1361.850    1.5775265
0.1149067
##  WT - Hom      8       -0.4427511     2.229724    1373.390    -0.1985676
0.8426304
##  WT - Hom      9       2.6471243      2.198675    1360.646    1.2039636
0.2288130
##  WT - Hom      10      -3.7267161     2.216802    1392.952    -1.6811228
0.0929632
```

Checking model assumptions

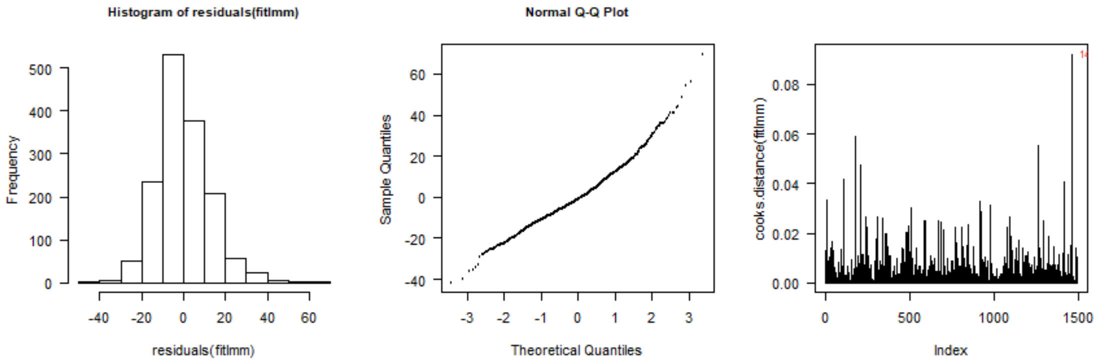

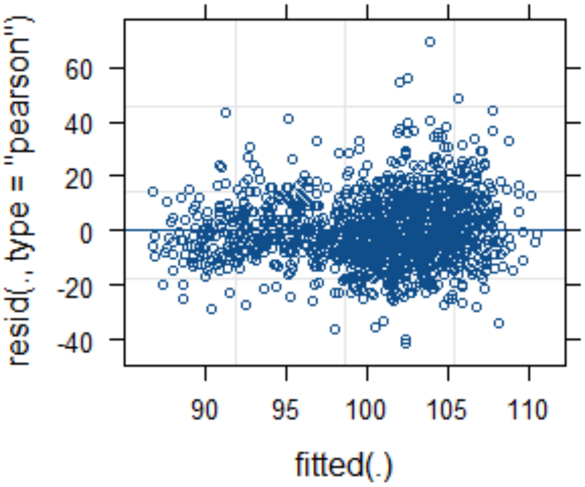

Conclusion

No significant difference was detected between the two groups (Type II Wald chi-square test; ^{ns}p = 0.664). Adj. Pvalue: ^{ns}p = 1 (for Meff = 7.456 tests).

Counterbend [ns]

Fitting LMM

```
## Warning in checkConv(attr(opt, "derivs"), opt$par, ctrl =
control$checkConv, :
## Model failed to converge with max|grad| = 0.00202687 (tol = 0.002,
component 1)

## Linear mixed model fit by REML ['lmerMod']
## Formula: value ~ FishGeno * time + (1 | Clutch/Fish)
##    Data: tmp
## REML criterion at convergence: 13016.92
## Random effects:
##  Groups      Name        Std.Dev.
##  Fish:Clutch (Intercept)  6.541
##  Clutch      (Intercept)  3.641
##  Residual                18.056
## Number of obs: 1495, groups:  Fish:Clutch, 147; Clutch, 4
## Fixed Effects:
##      (Intercept)       FishGenoHom              time  FishGenoHom:time
##          45.6827           -0.7760           -0.3027            0.5542
## convergence code 0; 1 optimizer warnings; 0 lme4 warnings

## Analysis of Deviance Table (Type II Wald chisquare tests)
##
## Response: value
##                 Chisq Df Pr(>Chisq)
## FishGeno       2.4433  1    0.11803
## time           0.0793  1    0.77822
## FishGeno:time 2.7922  1    0.09472 .
## ---
## Signif. codes:  0 '***' 0.001 '**' 0.01 '*' 0.05 '.' 0.1 ' ' 1
```

Group comparison at each trial

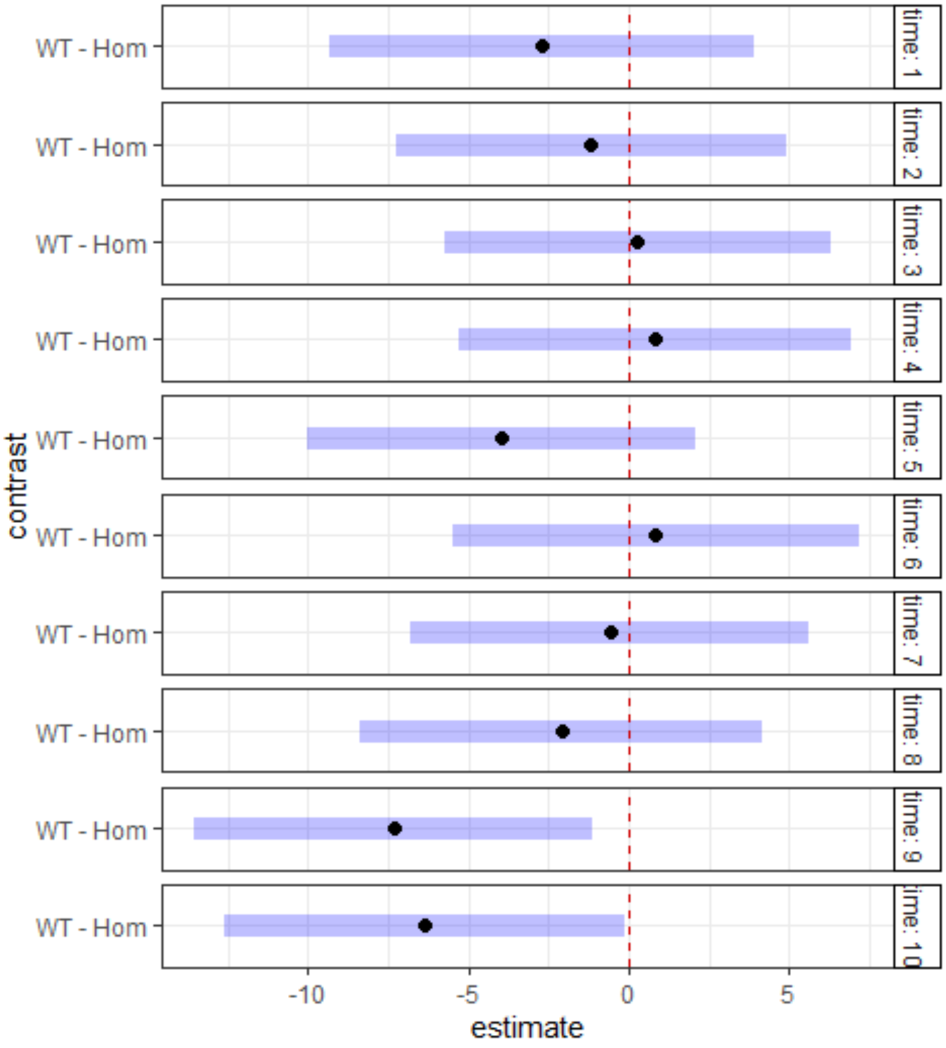


```
##
##
## Table: Post hoc Tukey's: Mean differences between groups at each trial
##
##  contrast    time     estimate        SE          df         t.ratio
p.value
## ----------  ------  ------------  ----------  ----------  ------------  --
---------
##  WT - Hom     1      -2.6926632    3.368051    1297.083    -0.7994723
0.4241630
##  WT - Hom     2      -1.1692153    3.110575    1224.261    -0.3758840
0.7070683
##  WT - Hom     3      0.2756393     3.068539    1207.292    0.0898275
0.9284392
##  WT - Hom     4      0.8036688     3.124670    1232.753    0.2572012
0.7970664
```

```
##   WT - Hom      5       -3.9833615    3.081405    1217.001    -1.2927097
0.1963568
##   WT - Hom      6       0.8491193     3.233092    1266.458    0.2626338
0.7928755
##   WT - Hom      7       -0.5952959    3.157987    1242.276    -0.1885049
0.8505117
##   WT - Hom      8       -2.1087115    3.199241    1260.283    -0.6591286
0.5099336
##   WT - Hom      9       -7.3305554    3.157560    1241.083    -2.3215889
0.0204163
##   WT - Hom      10      -6.3594786    3.177737    1274.006    -2.0012604
0.0455761
```

Checking model assumptions

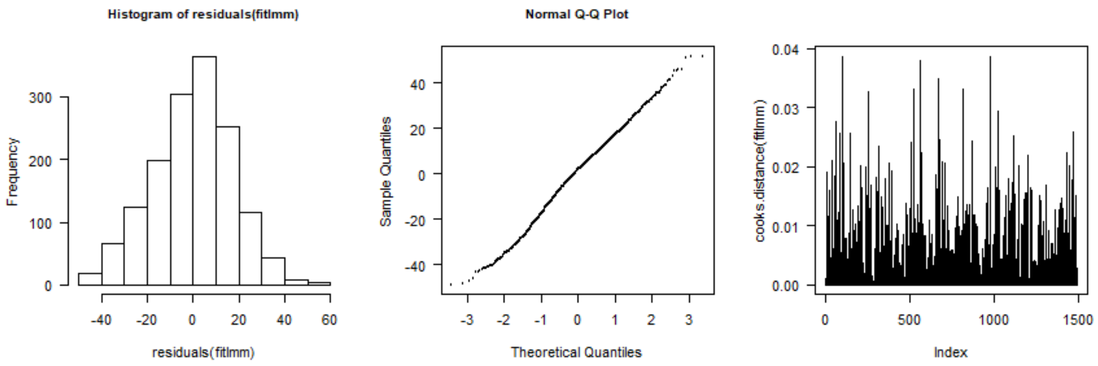

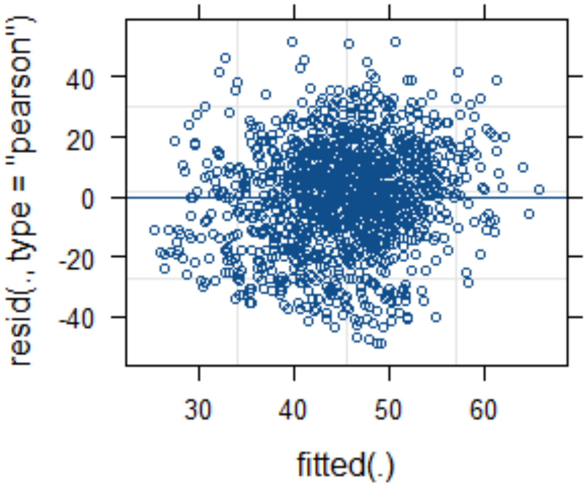

Conclusion

No significant difference was detected between the two groups (Type II Wald chi-square test; $^{ns}p = 0.118$). Adj. Pvalue: $^{ns}p = 0.880$ (for Meff = 7.456 tests).

Distance [ns]

Fitting LMM

```
## Linear mixed model fit by REML ['lmerMod']
## Formula: value ~ FishGeno * time + (1 | Clutch/Fish)
##    Data: tmp
## REML criterion at convergence: 6592.135
## Random effects:
##  Groups      Name        Std.Dev.
##  Fish:Clutch (Intercept) 1.0864
##  Clutch      (Intercept) 0.8443
##  Residual                2.0418
## Number of obs: 1495, groups:  Fish:Clutch, 147; Clutch, 4
## Fixed Effects:
##     (Intercept)       FishGenoHom              time  FishGenoHom:time
##         9.27267           0.55450          -0.07998          -0.04471
```

```
## Analysis of Deviance Table (Type II Wald chisquare tests)
##
## Response: value
##                  Chisq Df Pr(>Chisq)
## FishGeno       2.0704  1     0.1502
## time          28.8912  1  7.656e-08 ***
## FishGeno:time  1.4173  1     0.2338
## ---
## Signif. codes:  0 '***' 0.001 '**' 0.01 '*' 0.05 '.' 0.1 ' ' 1
```

Group comparison at each trial

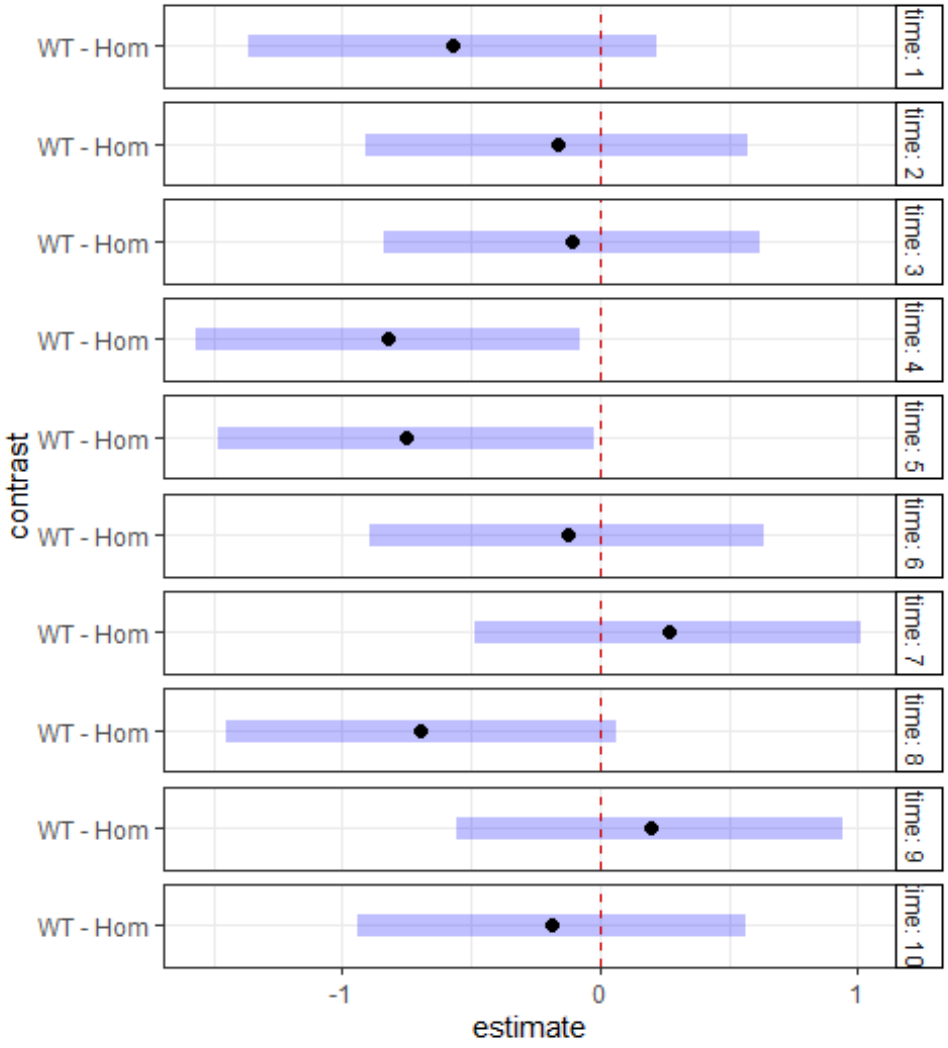


```
##
##
## Table: Post hoc Tukey's: Mean differences between groups at each trial
##
##  contrast    time     estimate         SE           df           t.ratio
p.value
## ----------  ------  ------------  -----------  -----------  ------------
-----------
##  WT - Hom     1      -0.5744247    0.4046534    1039.8291    -1.4195473
0.1560392
##  WT - Hom     2      -0.1666882    0.3766813    920.3871     -0.4425177
0.6582186
##  WT - Hom     3      -0.1119769    0.3722008    897.9990     -0.3008508
0.7635979
##  WT - Hom     4      -0.8228512    0.3781269    929.0854     -2.1761242
0.0297973
```

```
##  WT - Hom     5      -0.7528559    0.3734817    906.6431     -2.0157772
0.0441179
##  WT - Hom     6      -0.1289702    0.3898825    981.0622     -0.3307924
0.7408719
##  WT - Hom     7      0.2632344     0.3817751    944.1813     0.6895013
0.4906773
##  WT - Hom     8      -0.6947643    0.3861286    966.7891     -1.7993079
0.0722817
##  WT - Hom     9      0.1939219     0.3817563    943.3995     0.5079731
0.6115910
##  WT - Hom     10     -0.1853175    0.3833664    965.7667     -0.4833952
0.6289247
```

Checking model assumptions

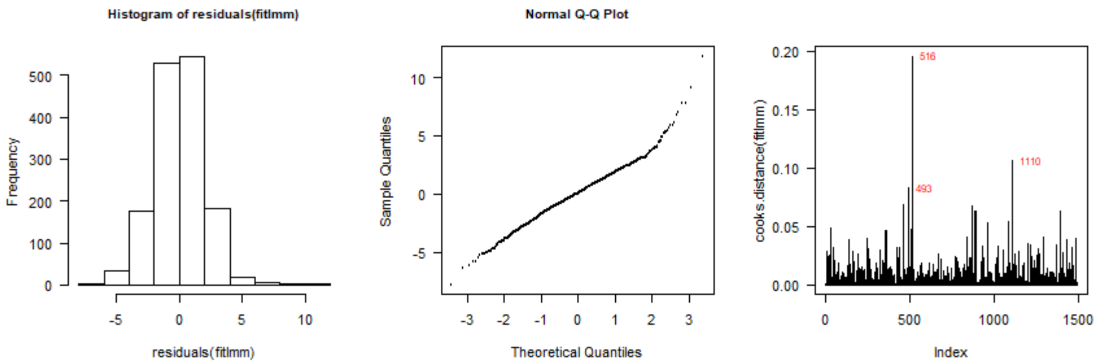

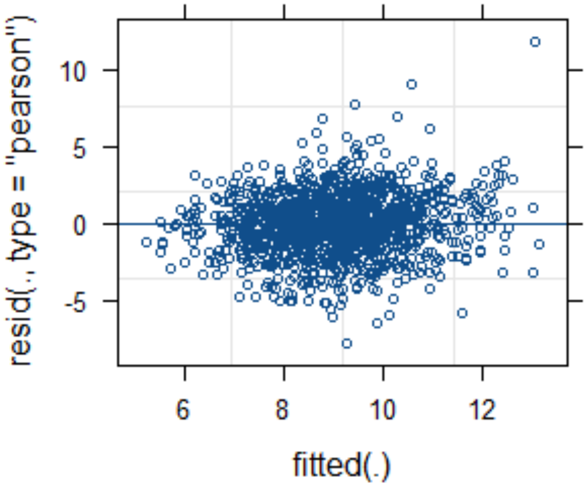

Conclusion

No significant difference was detected between the two groups (Type II Wald chi-square test; ^{ns}p = 0.150). Adj. Pvalue: ^{ns}p = 1 (for Meff = 7.456 tests). Significant effect of trial (Type II Wald chi-square test; ^{ns}p = 7.7e-8). Adj. Pvalue: ***p = 5.7e-7 (for Meff = 7.456 tests).

Duration [sqrt, ns]

Fitting LMM

```
## Linear mixed model fit by REML ['lmerMod']
## Formula: sqrt(value) ~ FishGeno * time + (1 | Clutch/Fish)
##    Data: tmp
## REML criterion at convergence: -3749.684
## Random effects:
##  Groups      Name        Std.Dev.
##  Fish:Clutch (Intercept) 0.04500 
##  Clutch      (Intercept) 0.01756 
##  Residual                0.06226 
## Number of obs: 1495, groups:  Fish:Clutch, 147; Clutch, 4
## Fixed Effects:
##      (Intercept)       FishGenoHom              time  FishGenoHom:time  
##         0.505666          0.004041          0.001381         -0.001818  

## Analysis of Deviance Table (Type II Wald chisquare tests)
## 
## Response: sqrt(value)
##                Chisq Df Pr(>Chisq)
## FishGeno      0.5334  1     0.4652
## time          0.8942  1     0.3443
## FishGeno:time 2.5174  1     0.1126
```

Group comparison at each trial

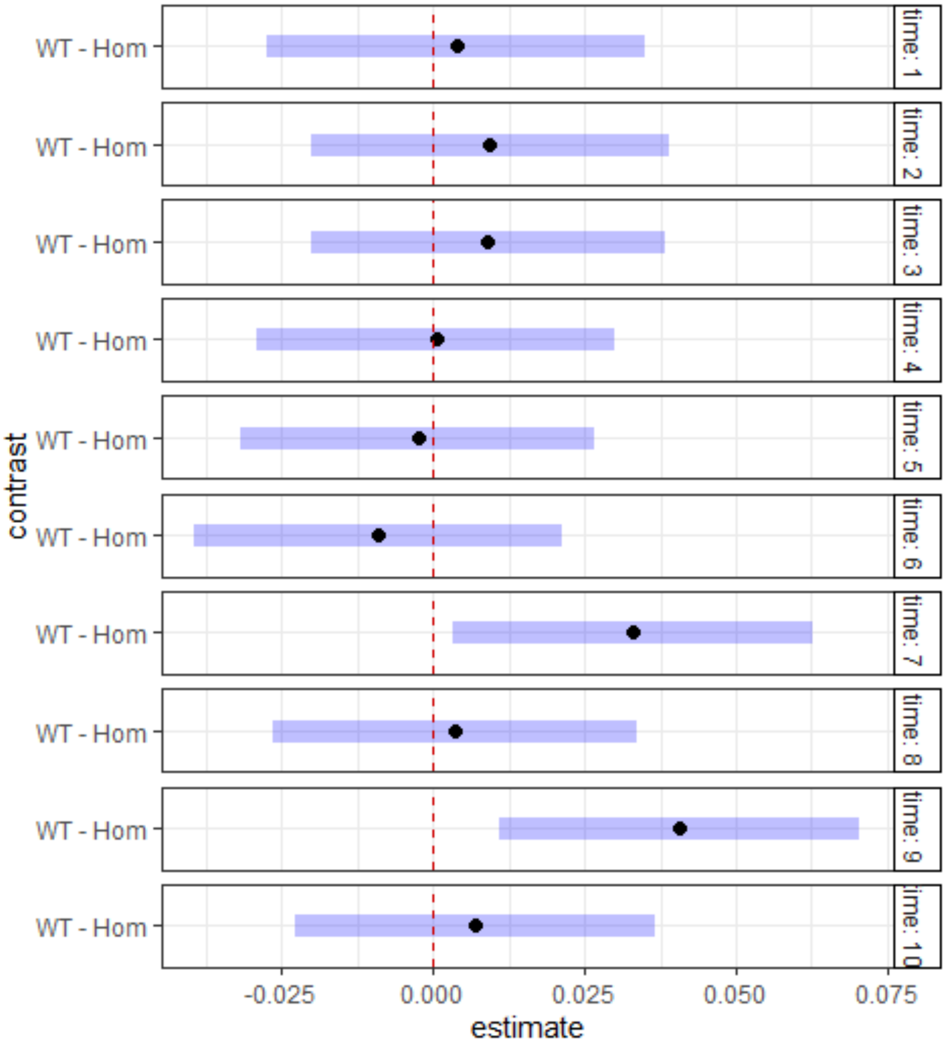


```
##
##
## Table: Post hoc Tukey's: Mean differences between groups at each trial
##
##  contrast    time     estimate        SE          df        t.ratio
p.value
## ----------  ------  ------------  -----------  ----------  ------------  -
----------
##  WT - Hom     1      0.0039350     0.0158774    680.7208    0.2478385
0.8043341
##  WT - Hom     2      0.0094118     0.0149821    578.1665    0.6282011
0.5301203
##  WT - Hom     3      0.0090667     0.0148419    561.8553    0.6108859
0.5415222
##  WT - Hom     4      0.0005141     0.0150262    583.7883    0.0342115
0.9727202
```

```
##  WT - Hom     5      -0.0024212    0.0148797    566.7633    -0.1627180
0.8707985
##  WT - Hom     6      -0.0089754    0.0154020    626.6782    -0.5827408
0.5602774
##  WT - Hom     7      0.0329990     0.0151439    596.5782    2.1790312
0.0297199
##  WT - Hom     8      0.0036830     0.0152800    613.2545    0.2410359
0.8096078
##  WT - Hom     9      0.0406323     0.0151442    596.4503    2.6830283
0.0074983
##  WT - Hom     10     0.0069605     0.0151824    605.2845    0.4584559
0.6467895
```

Checking model assumptions

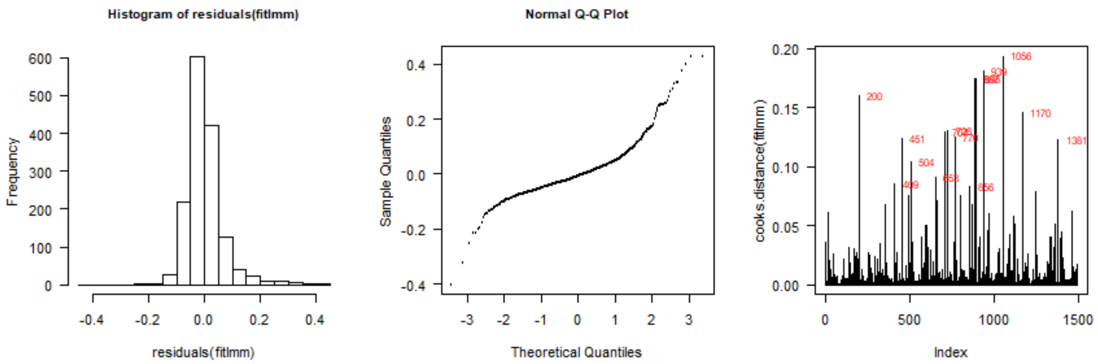

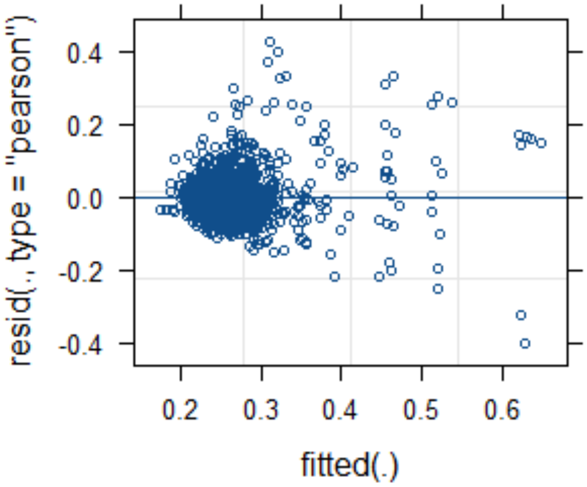

Conclusion

No significant difference was detected between the two groups (Type II Wald chi-square test; $^{ns}p = 0.465$). Adj. Pvalue: $^{ns}p = 1$ (for Meff = 7.456 tests).

Latency [ns]

Fitting LMM

```
## Linear mixed model fit by REML ['lmerMod']
## Formula: value ~ FishGeno * time + (1 | Clutch/Fish)
##    Data: tmp
## REML criterion at convergence: 5584.152
## Random effects:
##  Groups      Name        Std.Dev.
##  Fish:Clutch (Intercept) 0.8489
##  Clutch      (Intercept) 1.0080
##  Residual                1.4444
## Number of obs: 1495, groups:  Fish:Clutch, 147; Clutch, 4
## Fixed Effects:
##     (Intercept)       FishGenoHom              time  FishGenoHom:time
##         4.74014           0.20958           0.05271           0.02903
```

```
## Analysis of Deviance Table (Type II Wald chisquare tests)
##
## Response: value
##                 Chisq Df Pr(>Chisq)
## FishGeno       5.1803  1    0.02284 *
## time          24.9038  1  6.026e-07 ***
## FishGeno:time  1.1931  1    0.27471
## ---
## Signif. codes:  0 '***' 0.001 '**' 0.01 '*' 0.05 '.' 0.1 ' ' 1
```

Group comparison at each trial

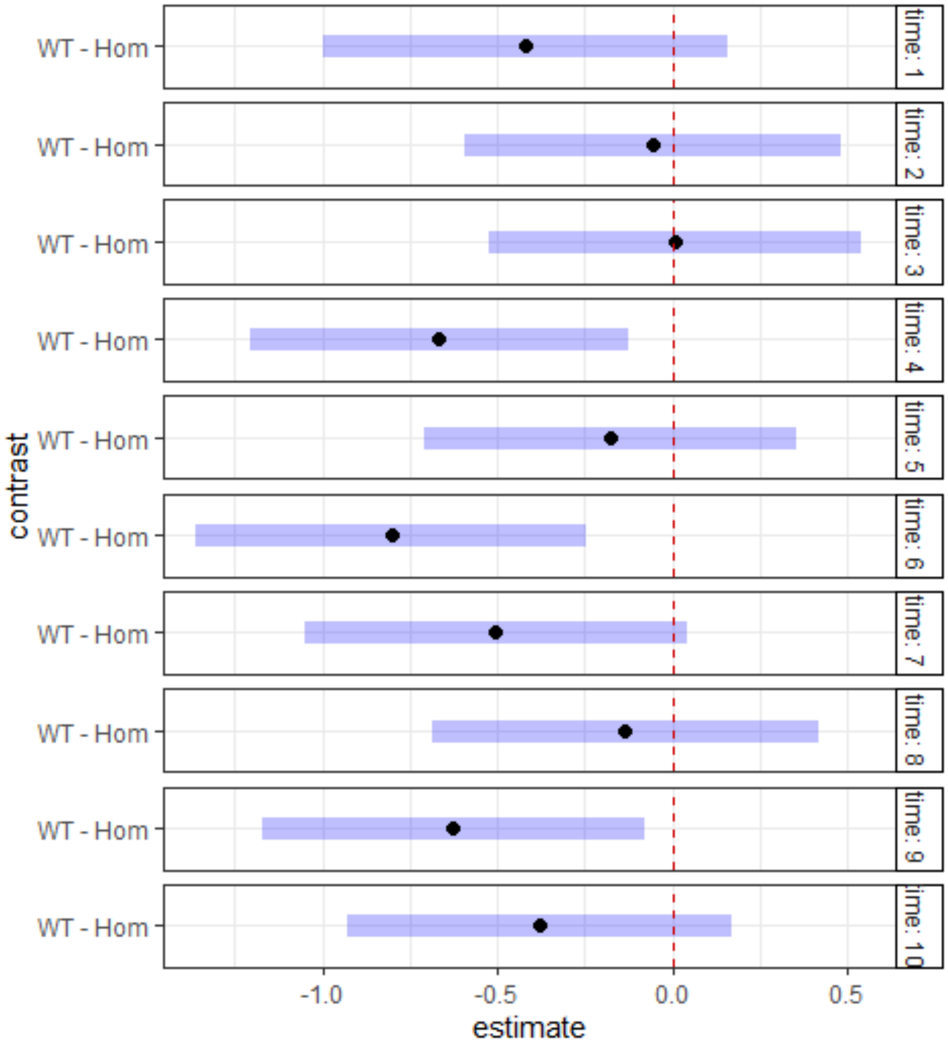


```
##
##
## Table: Post hoc Tukey's: Mean differences between groups at each trial
##
##  contrast    time     estimate        SE          df         t.ratio
p.value
## ----------  ------  ------------  -----------  ----------  ------------  -
----------
##  WT - Hom     1      -0.4191059    0.2942862    954.7892    -1.4241440
0.1547315
##  WT - Hom     2      -0.0551944    0.2746788    832.8438    -0.2009416
0.8407933
##  WT - Hom     3      0.0078746     0.2715509    810.9336    0.0289985
0.9768729
##  WT - Hom     4      -0.6679505    0.2756796    841.0108    -2.4229232
0.0156064
```

```
##  WT - Hom      5       -0.1764765    0.2724339    818.8694    -0.6477774
0.5173105
##  WT - Hom      6       -0.8024799    0.2839149    893.3670    -2.8264800
0.0048113
##  WT - Hom      7       -0.5042061    0.2782424    856.3602    -1.8121114
0.0703191
##  WT - Hom      8       -0.1355836    0.2812741    878.3543    -0.4820338
0.6299020
##  WT - Hom      9       -0.6257956    0.2782323    855.7177    -2.2491840
0.0247544
##  WT - Hom      10      -0.3806198    0.2792801    874.4088    -1.3628605
0.1732773
```

Checking model assumptions

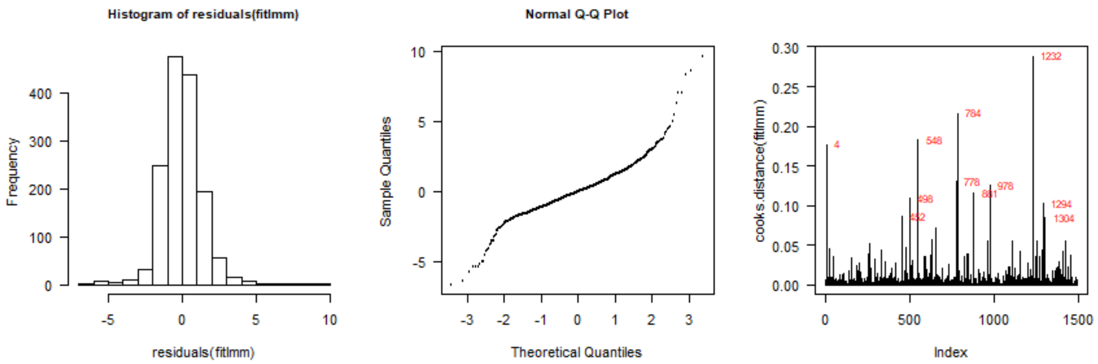

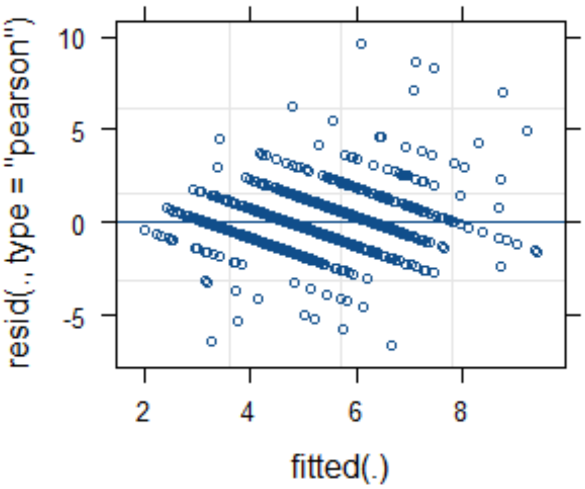

Conclusion

Linear mixed model indicated a significant difference between the two groups (Type II test chi-square test; $^{*}p = 0.023$). Adj. Pvalue: $^{ns}p = 0.170$ (using Meff = 7.456 tests). Significant effect of trial (Type II test chi-square test; $^{***}p = 6.0e\text{-}7$). Adj. Pvalue: $^{***}p = 4.5e\text{-}6$. But no interaction between trial and genotype (Type II test chi-square test; $^{ns}p = 0.275$). Adj. Pvalue: $^{ns}p = 1$.

mTBF [ns]

Fitting LMM

```
## Linear mixed model fit by REML ['lmerMod']
## Formula: value ~ FishGeno * time + (1 | Clutch/Fish)
##    Data: tmp
## REML criterion at convergence: 10356.23
## Random effects:
##  Groups      Name        Std.Dev.
##  Fish:Clutch (Intercept) 3.376
##  Clutch      (Intercept) 4.199
##  Residual                7.277
## Number of obs: 1495, groups:  Fish:Clutch, 147; Clutch, 4
## Fixed Effects:
##     (Intercept)       FishGenoHom              time  FishGenoHom:time
##        39.02143           0.03436          -0.15878          -0.08045
```

```
## Analysis of Deviance Table (Type II Wald chisquare tests)
##
## Response: value
##                 Chisq Df Pr(>Chisq)
## FishGeno      0.3545  1   0.551570
## time          8.6308  1   0.003305 **
## FishGeno:time 0.3616  1   0.547630
## ---
## Signif. codes:  0 '***' 0.001 '**' 0.01 '*' 0.05 '.' 0.1 ' ' 1
```

Group comparison at each trial

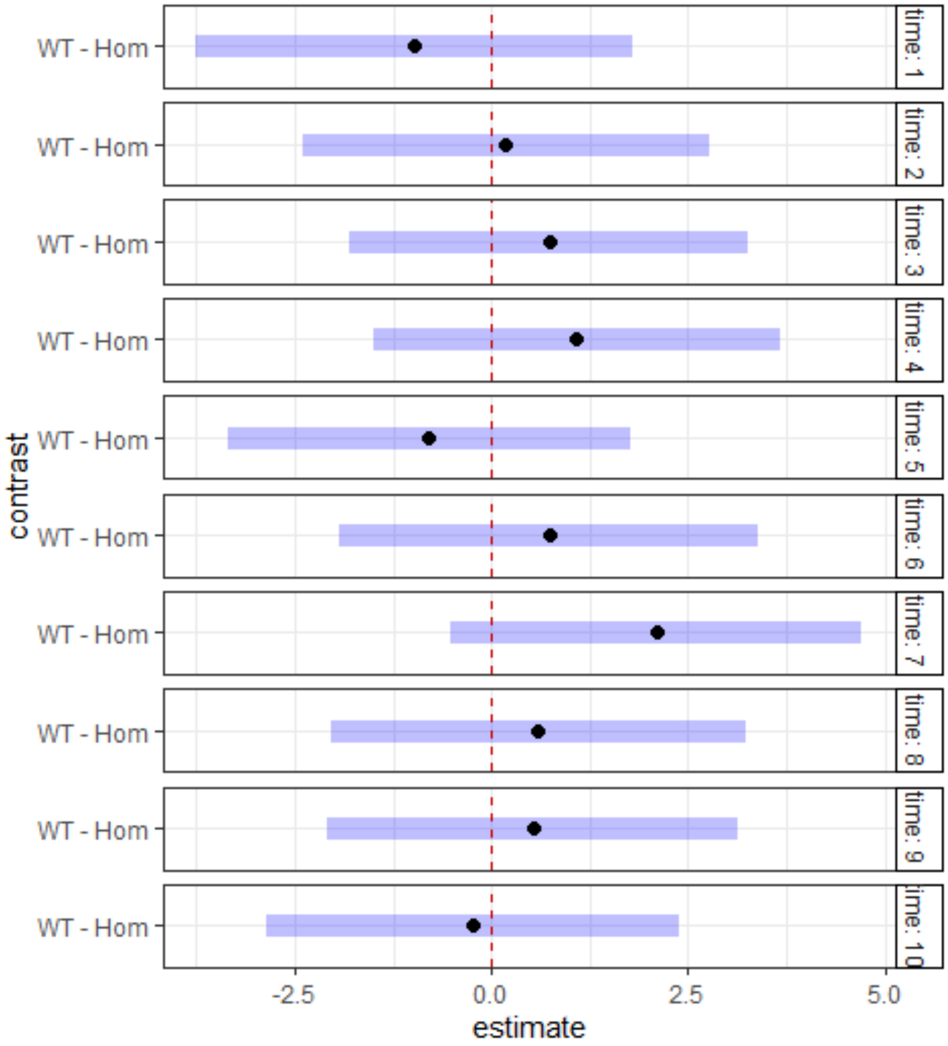


```
##
##
## Table: Post hoc Tukey's: Mean differences between groups at each trial
##
##  contrast    time     estimate        SE          df         t.ratio
p.value
## ----------  ------  ------------  ----------  ----------  ------------  --
---------
##  WT - Hom     1      -0.9734106    1.413502    1159.971    -0.6886517
0.4911802
##  WT - Hom     2      0.1873990     1.310815    1054.264    0.1429637
0.8863462
##  WT - Hom     3      0.7325100     1.294222    1032.464    0.5659846
0.5715272
##  WT - Hom     4      1.0800108     1.316239    1063.311    0.8205277
0.4120992
```

```
##  WT - Hom     5      -0.7898452    1.299096    1042.242    -0.6079959
0.5433227
##  WT - Hom     6      0.7280732     1.359414    1110.099    0.5355787
0.5923570
##  WT - Hom     7      2.0921642     1.329584    1076.796    1.5735478
0.1158858
##  WT - Hom     8      0.5925692     1.345742    1098.578    0.4403290
0.6597854
##  WT - Hom     9      0.5256992     1.329468    1075.630    0.3954207
0.6926109
##  WT - Hom     10     -0.2473383    1.336163    1103.474    -0.1851109
0.8531761
```

Checking model assumptions

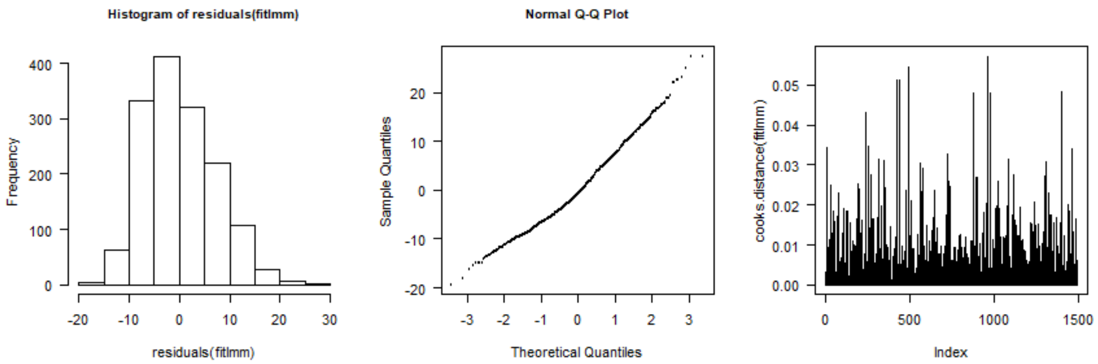

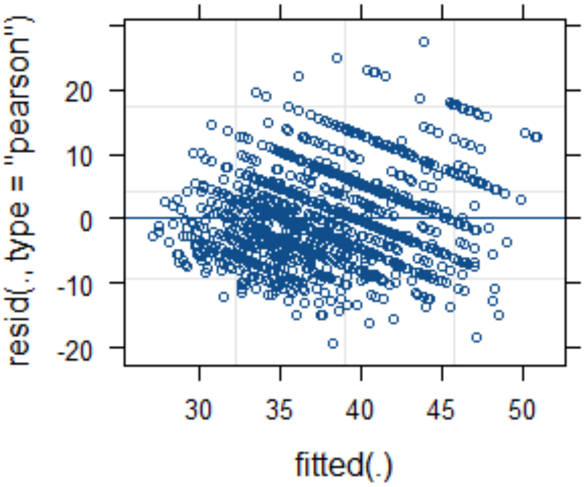

Conclusion

No significant difference was detected between the two groups (Type II Wald chi-square test; ^{ns}p = 0.552). Adj. Pvalue: ^{ns}p = 1 (for Meff = 7.456 tests). Significant effect of trial (Type II Wald chi-square test; **p = 3.3e-3). Adj. Pvalue: *p = 0.025 (for Meff = 7.456 tests).

Number of oscillations [ns]

Fitting LMM

```
## Linear mixed model fit by REML ['lmerMod']
## Formula: value ~ FishGeno * time + (1 | Clutch/Fish)
##    Data: tmp
## REML criterion at convergence: 7487.87
## Random effects:
##  Groups      Name        Std.Dev.
##  Fish:Clutch (Intercept) 1.6056
##  Clutch      (Intercept) 0.6045
##  Residual                2.7415
## Number of obs: 1495, groups:  Fish:Clutch, 147; Clutch, 4
## Fixed Effects:
##     (Intercept)       FishGenoHom              time  FishGenoHom:time
##         8.29158           0.09926           0.02233          -0.06845
```

```
## Analysis of Deviance Table (Type II Wald chisquare tests)
##
## Response: value
##                Chisq Df Pr(>Chisq)
## FishGeno      0.8302  1     0.3622
## time          0.1371  1     0.7111
## FishGeno:time 1.8414  1     0.1748
```

Group comparison at each trial

```
## Warning in checkConv(attr(opt, "derivs"), opt$par, ctrl =
control$checkConv, :
## Model failed to converge with max|grad| = 0.00422999 (tol = 0.002,
component 1)
```

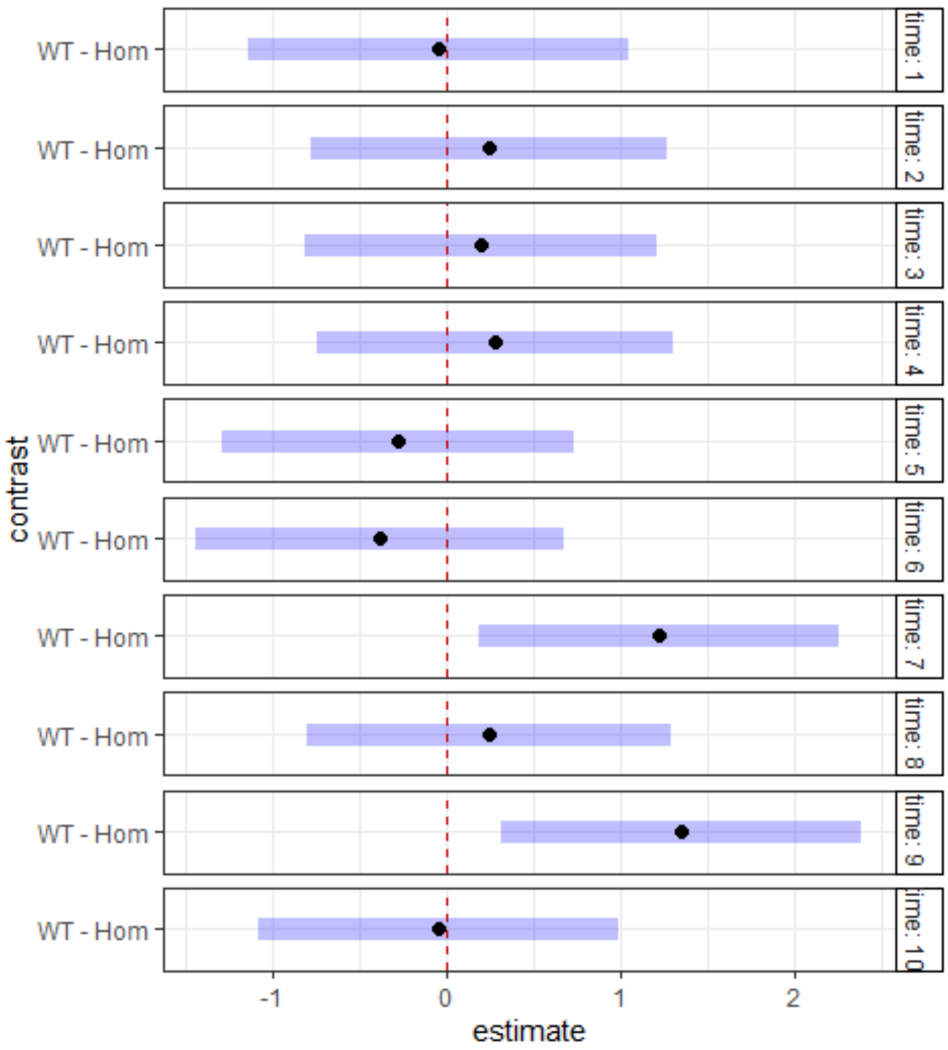


```
##
##
## Table: Post hoc Tukey's: Mean differences between groups at each trial
##
##  contrast    time     estimate         SE           df         t.ratio
p.value
## ----------  ------  ------------  -----------  ----------  ------------  -
----------
##  WT - Hom      1      -0.0403670    0.5575297    964.1214    -0.0724032
0.9422960
##  WT - Hom      2      0.2431884     0.5204058    841.6949    0.4673055
0.6404023
```

```
##  WT - Hom     3      0.1961259     0.5145035    820.0168    0.3811945
0.7031576
##  WT - Hom     4      0.2783928     0.5223010    849.9094    0.5330123
0.5941644
##  WT - Hom     5      -0.2813587    0.5161586    827.6511    -0.5451011
0.5858308
##  WT - Hom     6      -0.3810672    0.5379080    902.8423    -0.7084246
0.4788645
##  WT - Hom     7      1.2245822     0.5271519    865.2882    2.3230156
0.0204093
##  WT - Hom     8      0.2422537     0.5328998    887.6266    0.4545953
0.6495116
##  WT - Hom     9      1.3529076     0.5271440    864.8153    2.5664856
0.0104408
##  WT - Hom     10     -0.0453025    0.5291317    883.7638    -0.0856166
0.9317906
```

Checking model assumptions

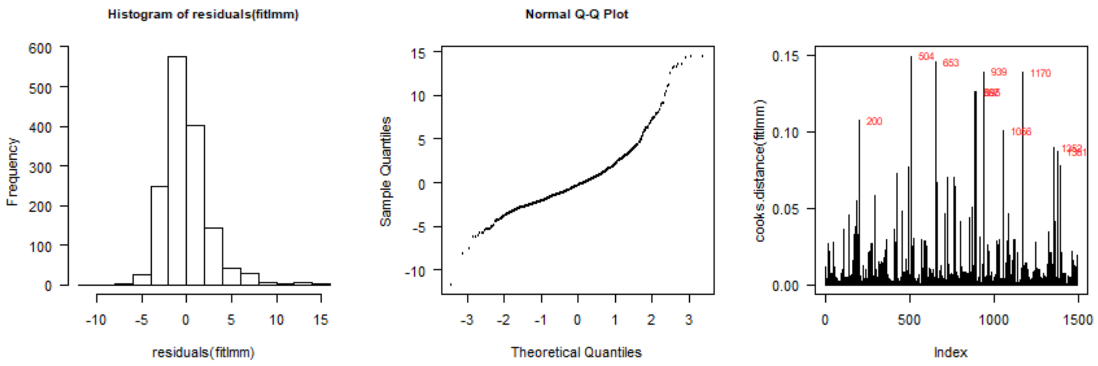

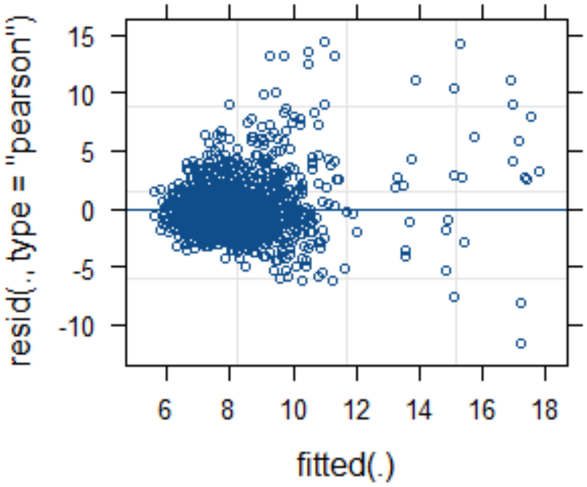

Conclusion

No significant difference was detected between the two groups (Type II Wald chi-square test; ^{ns}p = 0.362). Adj. Pvalue: ^{ns}p = 1 (for Meff = 7.456 tests).

Speed [ns]

Fitting LMM

```
## Linear mixed model fit by REML ['lmerMod']
## Formula: value ~ FishGeno * time + (1 | Clutch/Fish)
##    Data: tmp
## REML criterion at convergence: 11125.9
## Random effects:
##  Groups      Name        Std.Dev.
##  Fish:Clutch (Intercept) 5.888
##  Clutch      (Intercept) 2.041
##  Residual                9.226
## Number of obs: 1495, groups:  Fish:Clutch, 147; Clutch, 4
## Fixed Effects:
##     (Intercept)       FishGenoHom              time  FishGenoHom:time
##        38.03022           0.69723          -0.50099           0.05298

## Analysis of Deviance Table (Type II Wald chisquare tests)
##
## Response: value
##                  Chisq Df Pr(>Chisq)
## FishGeno        0.8035  1     0.3701
## time           31.6810  1  1.817e-08 ***
## FishGeno:time   0.0974  1     0.7550
## ---
## Signif. codes:  0 '***' 0.001 '**' 0.01 '*' 0.05 '.' 0.1 ' ' 1
```

Group comparison at each trial

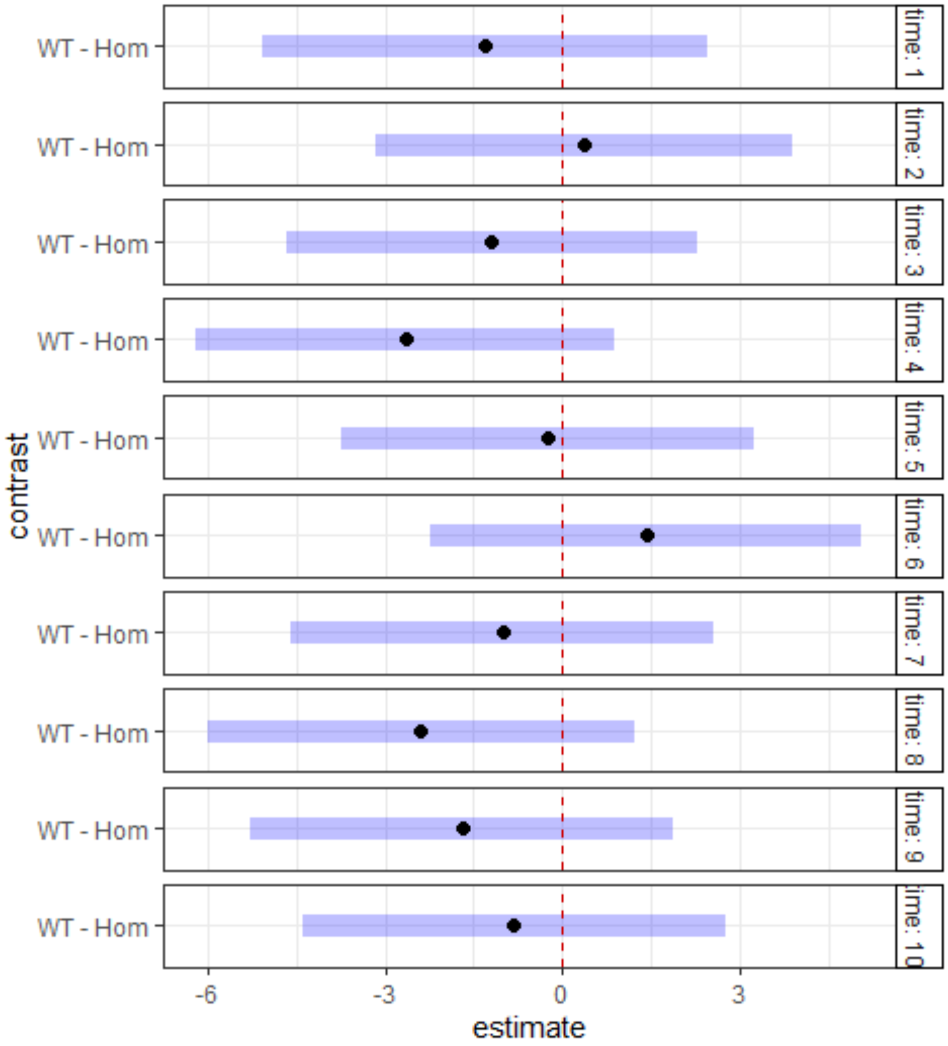


```
##
##
## Table: Post hoc Tukey's: Mean differences between groups at each trial
##
##  contrast    time     estimate        SE          df         t.ratio
p.value
## ----------  ------  ------------  ----------  ----------  ------------  --
---------
##  WT - Hom     1      -1.3004345    1.918724    882.8071    -0.6777601
0.4981014
##  WT - Hom     2      0.3646247     1.796033    761.8937    0.2030167
0.8391762
##  WT - Hom     3      -1.1879440    1.776618    741.2595    -0.6686546
0.5039240
##  WT - Hom     4      -2.6452924    1.802227    769.4645    -1.4677907
0.1425697
```

```
##  WT - Hom    5     -0.2423898    1.781993    748.1265    -0.1360217
0.8918407
##  WT - Hom    6     1.4195334     1.853791    821.1744    0.7657462
0.4440474
##  WT - Hom    7     -1.0073247    1.818289    784.6014    -0.5539959
0.5797395
##  WT - Hom    8     -2.3927226    1.837183    805.8301    -1.3023868
0.1931563
##  WT - Hom    9     -1.6926888    1.818289    784.2603    -0.9309237
0.3521795
##  WT - Hom    10    -0.8185861    1.824428    799.9087    -0.4486810
0.6537833
```

Checking model validity

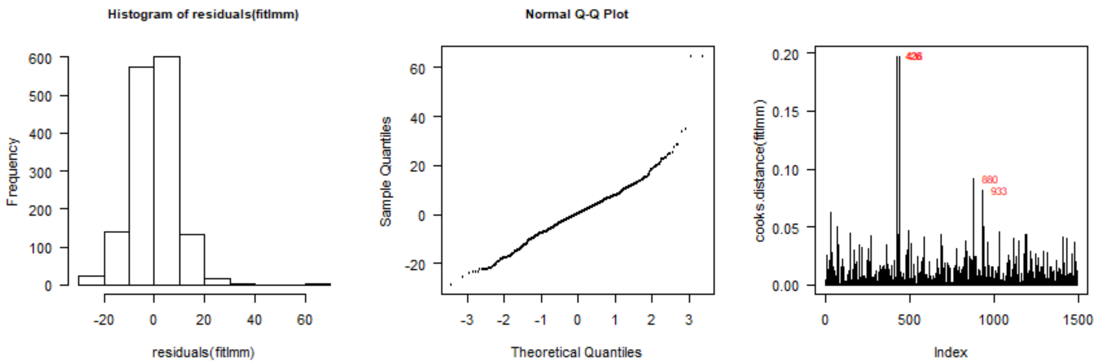

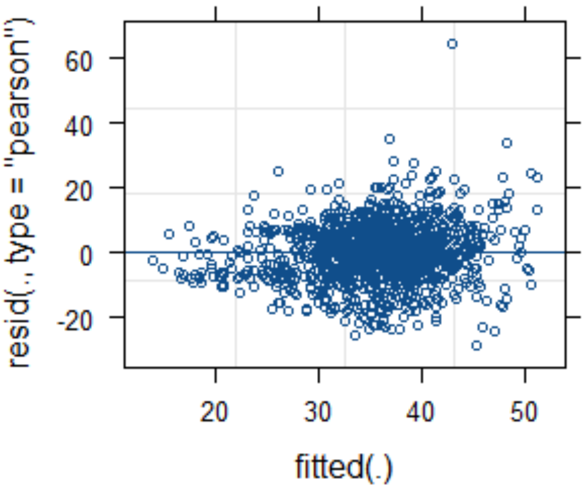

Conclusion

No significant difference was detected between the two groups (Type II Wald chi-square test; ^{ns}p = 0.370). Adj. Pvalue: ^{ns}p = 1 (for Meff = 7.456 tests). Significant effect of trial (Type II Wald chi-square test; ***p = 1.8e-8). Adj. Pvalue: ***p = 1.4e-7 (for Meff = 7.456 tests).

R session information

```
## R version 3.6.1 (2019-07-05)
## Platform: x86_64-w64-mingw32/x64 (64-bit)
## Running under: Windows 10 x64 (build 17134)
##
## Matrix products: default
##
## locale:
## [1] LC_COLLATE=French_France.1252  LC_CTYPE=French_France.1252
## [3] LC_MONETARY=French_France.1252 LC_NUMERIC=C
## [5] LC_TIME=French_France.1252
##
## attached base packages:
## [1] stats     graphics  grDevices utils     datasets  methods   base
##
## other attached packages:
##  [1] corrplot_0.84      emmeans_1.4.5      readxl_1.3.1
reshape2_1.4.3
##  [5] lme4_1.1-21        Matrix_1.2-18      car_3.0-7          carData_3.0-
3
##  [9] plyr_1.8.6         data.table_1.12.8  knitr_1.28
RColorBrewer_1.1-2
## [13] ggplot2_3.3.0
##
## loaded via a namespace (and not attached):
##  [1] Rcpp_1.0.4        mvtnorm_1.1-0     lattice_0.20-41   assertthat_0.2.1
##  [5] digest_0.6.25     R6_2.4.1          cellranger_1.1.0  evaluate_0.14
##  [9] highr_0.8         pillar_1.4.3      rlang_0.4.5       curl_4.3
## [13] minqa_1.2.4       nloptr_1.2.2.1    rmarkdown_2.1     labeling_0.3
## [17] splines_3.6.1     stringr_1.4.0     foreign_0.8-76    munsell_0.5.0
## [21] compiler_3.6.1    xfun_0.12         pkgconfig_2.0.3   htmltools_0.4.0
## [25] tidyselect_1.0.0 tibble_3.0.0       rio_0.5.16        fansi_0.4.1
## [29] crayon_1.3.4      dplyr_0.8.5       withr_2.1.2       MASS_7.3-51.5
## [33] grid_3.6.1        nlme_3.1-145      xtable_1.8-4      gtable_0.3.0
## [37] lifecycle_0.2.0   magrittr_1.5      scales_1.1.0      zip_2.0.4
## [41] estimability_1.3 cli_2.0.2          stringi_1.4.6     farver_2.0.3
## [45] ellipsis_0.3.0    vctrs_0.2.4       boot_1.3-24       openxlsx_4.1.4
## [49] tools_3.6.1       forcats_0.5.0     glue_1.3.2        purrr_0.3.3
## [53] hms_0.5.3         parallel_3.6.1    pbkrtest_0.4-8.6 abind_1.4-5
## [57] yaml_2.2.1        colorspace_1.4-1 haven_2.2.0
```
