# Supplementary material for: Somatostatin 1.1 contributes to the innate exploration of zebrafish larva
Source: Sci Rep. 2020 Sep 17;10:15235. doi: 10.1038/s41598-020-72039-x (PMC7499426; doi:10.1038/s41598-020-72039-x)
Supplement: Supplementary file 2 — Supplementary Information 2. [file 41598_2020_72039_MOESM2_ESM.pdf]

# Slow swim analysis

*FX Lejeune - iCONICS*

*2020-07-02*

## Contents

**1 Import data from files** **2**

**2 Method** **2**

**3 Forward swim** **3**
3.1 Descriptive statistics . . . . . 3
3.2 Forward correlation matrix . . . . . 4
3.3 Forward swim BoutDistance [sqrt, *] . . . . . 5
3.4 Forward swim BoutDuration [sqrt, *] . . . . . 9
3.5 Forward swim MedianBendAmp [log, *] . . . . . 13
3.6 Forward swim NumOfOsc [ns] . . . . . 17
3.7 Forward swim Speed [log, *] . . . . . 21
3.8 Forward swim TBF [ns] . . . . . 25

**4 Routine turn** **29**
4.1 Descriptive statistics . . . . . 29
4.2 All Escape correlation matrix . . . . . 30
4.3 Routine turn BoutDistance [sqrt, ns] . . . . . 31
4.4 Routine turn Duration [sqrt, ns] . . . . . 35
4.5 Routine turn MedianBendAmp [log, ns] . . . . . 39
4.6 Routine turn NumOfOsc [ns] . . . . . 43
4.7 Routine turn Speed [ns] . . . . . 47
4.8 Routine turn TBF [ns] . . . . . 51

**5 All bouts** **55**
5.1 Descriptive statistics . . . . . 55

**6 All bouts** **56**
6.1 All bouts BoutRate [ns] . . . . . 56
6.2 All bouts Distribution of MaxBendAmp . . . . . 60

**7 R session information** **61**

Statistical analysis for Feng Quan and Claire Wyart

# 1 Import data from files

6 kinematic parameters in ForwardSwin and in RoutineTurn: BoutDuration, NumberOfOscillations, BoutDistance, Speed, TBF and MedianBendAmp

Analysis is repeated in ForwardSwim (MaxBendAmplitude $<$ 25) and RoutineTurn (MaxBendAmplitude $>$ 25)

Fish number 204 (WT) was removed as it had a morphological deformity (as seen in PCA and videos)

# 2 Method

All statistical analyses were conducted using R version 3.6.1 (https://www.R-project.org/), and plots were generated with the ggplot2 package.

The correlation plots based on Spearman's rank correlations were made using the corrplot package.

Between-group comparison of SlowSwim data was conducted through a linear mixed model using the lmer function in the lme4 package. Significance for the main effect of genotype (Wild-type 'WT' vs Homozygous mutant 'Hom') was then evaluated using the Anova function in the car package with a Type II Wald chi-square test. To better meet the model assumptions (normality and homoscedasticity of residuals), data were transformed by the natural logarithm or the square root function prior to modeling.

For data representation, the estimated marginal means and model-based standard errors on the original scale (non-transformed data) were extracted for each condition using the emmeans package.

The level of statistical significance was set at $p < 0.05$ for all tests.

# 3 Forward swim

## 3.1 Descriptive statistics

Table 1: Forward swim WT - Mean values by clutch

| | WT_1 | WT_2 | WT_3 | WT_4 | MEAN |
|---|---|---|---|---|---|
| BoutDuration | 0.26 | 0.28 | 0.24 | 0.26 | 0.26 |
| NumberOfOscillations | 3.23 | 3.51 | 3.02 | 3.37 | 3.28 |
| BoutDistance | 0.50 | 0.50 | 0.39 | 0.51 | 0.48 |
| Speed | 1.82 | 1.75 | 1.59 | 1.89 | 1.76 |
| TBF | 11.50 | 12.12 | 11.69 | 12.17 | 11.87 |
| MedianBendAmp | 2.51 | 2.87 | 2.56 | 2.37 | 2.58 |

Table 2: Forward swim Hom - Mean values by clutch

| | Hom_1 | Hom_2 | Hom_3 | Hom_4 | MEAN |
|---|---|---|---|---|---|
| BoutDuration | 0.28 | 0.27 | 0.27 | 0.29 | 0.28 |
| NumberOfOscillations | 3.42 | 3.44 | 3.38 | 3.72 | 3.49 |
| BoutDistance | 0.57 | 0.51 | 0.54 | 0.66 | 0.57 |
| Speed | 1.98 | 1.86 | 1.91 | 2.19 | 1.98 |
| TBF | 11.42 | 11.74 | 11.71 | 11.92 | 11.70 |
| MedianBendAmp | 2.80 | 2.61 | 3.38 | 2.81 | 2.90 |

### 3.2 Forward correlation matrix

Using Spearman's rank correlations:

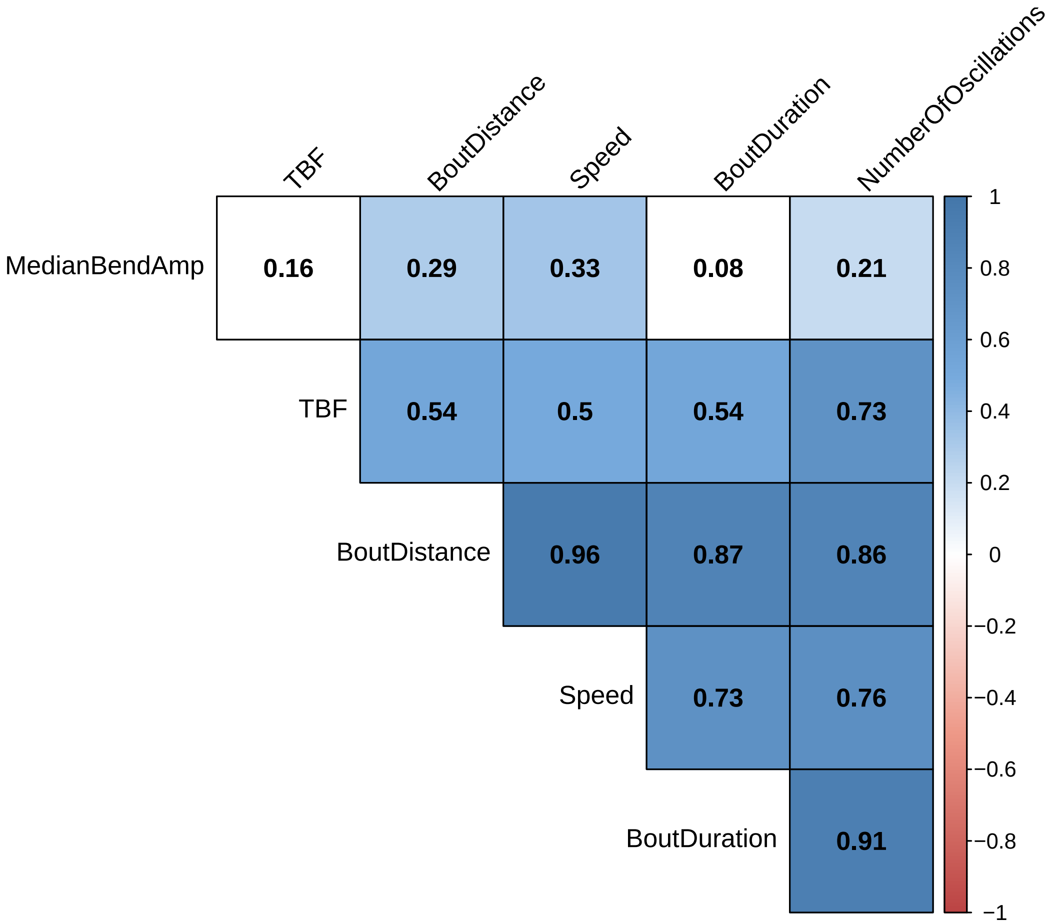


Based on 6 kinetic parameters, variance of eigenvalues of the correlation matrix is 2.396 leading to an effective number of independent tests (Meff) of 4.003 to be used for multiple testing correction. Method described in Nyholt DR, A simple correction for multiple testing for single-nucleotide polymorphisms in linkage disequilibrium with each other, Am J Hum Genet, 2004 [PMID: 14997420].

### 3.3 Forward swim BoutDistance [sqrt, *]

#### 3.3.1 Data visualization with clutch info

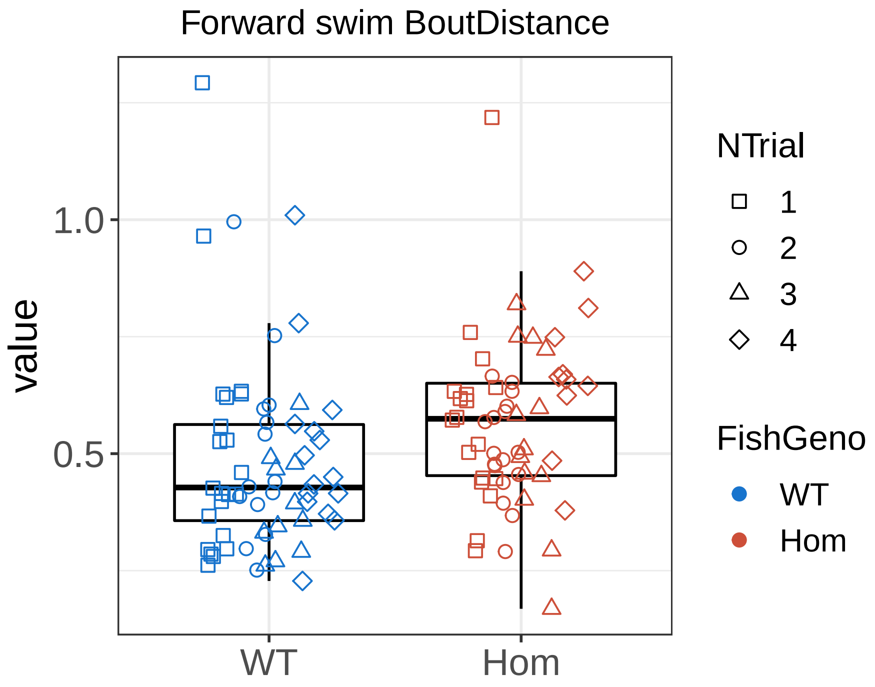


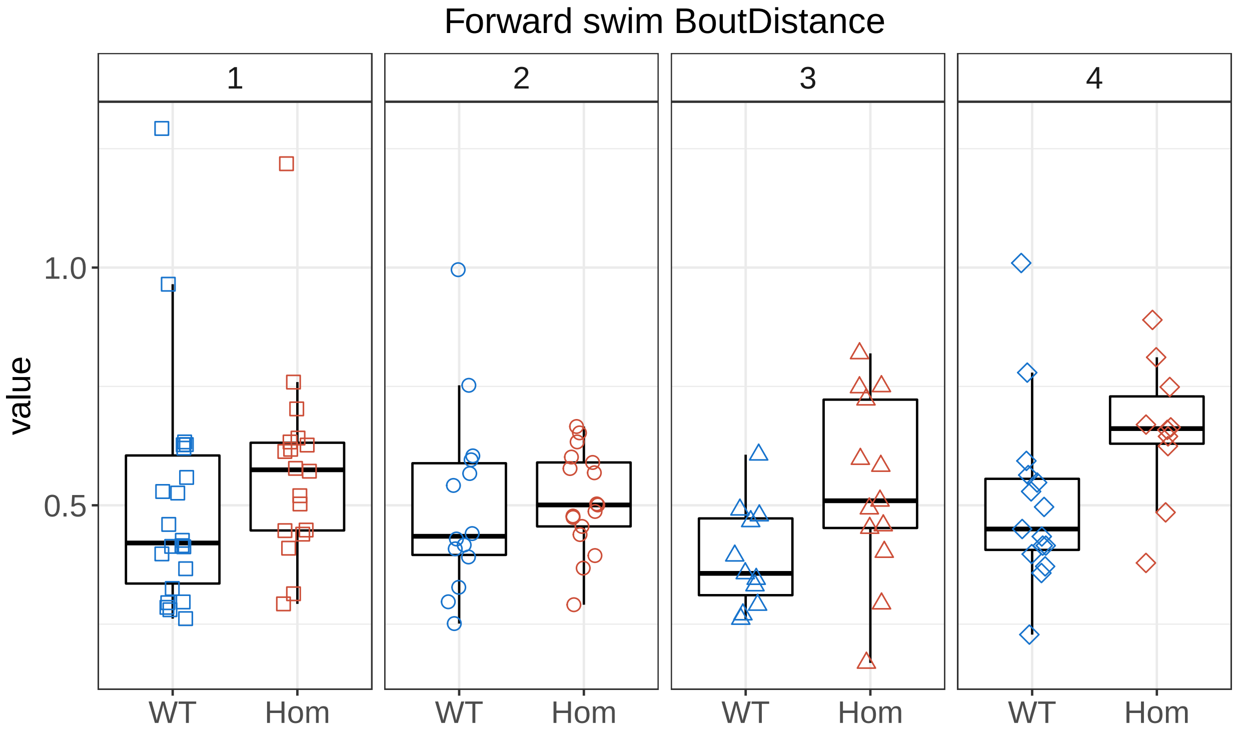

Table 3: Forward swim BoutDistance - Descriptive statistics

| | Min. | 1st Qu. | Median | Mean | 3rd Qu. | Max. |
|---|---|---|---|---|---|---|
| Hom_1 | 0.29 | 0.45 | 0.57 | 0.57 | 0.63 | 1.22 |
| Hom_2 | 0.29 | 0.46 | 0.50 | 0.51 | 0.59 | 0.67 |
| Hom_3 | 0.17 | 0.45 | 0.51 | 0.54 | 0.72 | 0.82 |
| Hom_4 | 0.38 | 0.63 | 0.66 | 0.66 | 0.73 | 0.89 |
| WT_1 | 0.26 | 0.34 | 0.42 | 0.50 | 0.60 | 1.29 |
| WT_2 | 0.25 | 0.40 | 0.43 | 0.50 | 0.59 | 1.00 |
| WT_3 | 0.26 | 0.31 | 0.36 | 0.39 | 0.47 | 0.61 |
| WT_4 | 0.23 | 0.41 | 0.45 | 0.51 | 0.56 | 1.01 |

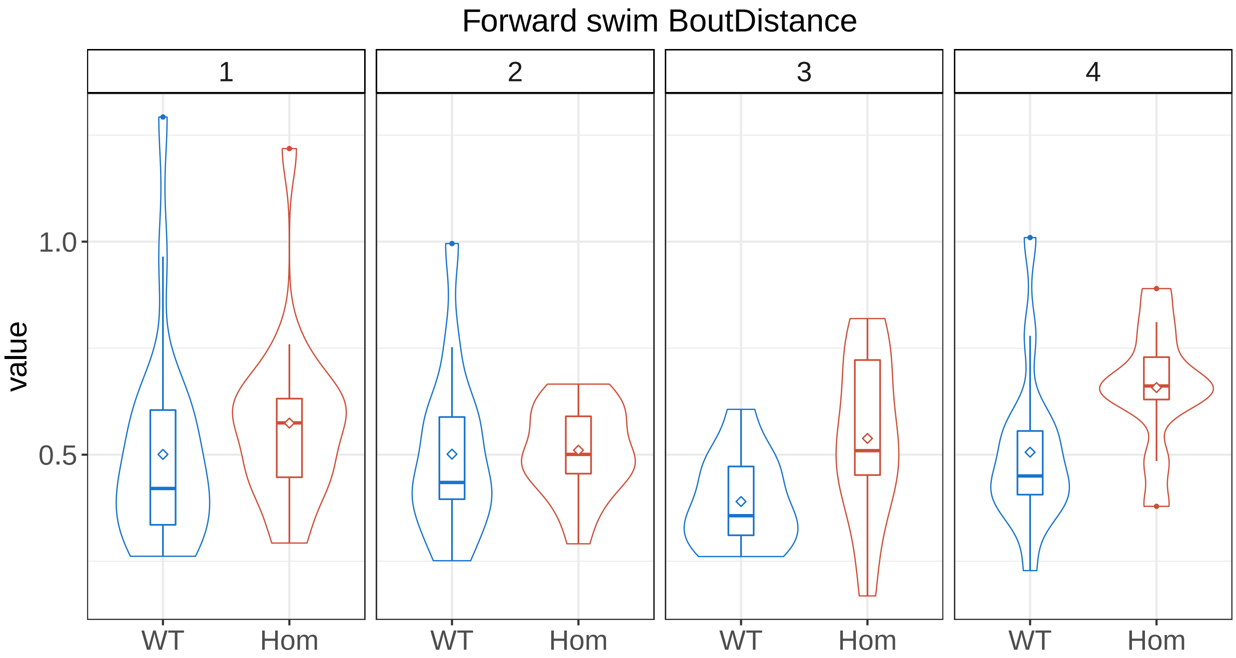

### 3.3.2 Fitting LMM

```
## Linear mixed model fit by REML ['lmerMod']
## Formula: sqrt(value) ~ FishGeno + (1 | NTrial)
##    Data: tmp
## REML criterion at convergence: -150.4576
## Random effects:
##  Groups   Name        Std.Dev.
##  NTrial   (Intercept) 0.01895
##  Residual             0.12272
## Number of obs: 120, groups:  NTrial, 4
## Fixed Effects:
## (Intercept)  FishGenoHom
##     0.68065      0.06091
```

```
## Analysis of Deviance Table (Type II Wald chisquare tests)
##
## Response: sqrt(value)
##           Chisq Df Pr(>Chisq)
## FishGeno 7.3396  1   0.006745 **
## ---
## Signif. codes:  0 '***' 0.001 '**' 0.01 '*' 0.05 '.' 0.1 ' ' 1
```

Estimated marginal means with model-based standard errors and 95% confidence limits on the original scale (non-transformed data):

```
## 'emmGrid' object with variables:
##     FishGeno = WT, Hom
## Transformation: "sqrt"
```

```
##  FishGeno response     SE   df lower.CL upper.CL
##  WT          0.463 0.0252 6.78    0.405    0.525
##  Hom         0.550 0.0280 7.48    0.486    0.617
##
## Degrees-of-freedom method: kenward-roger
## Confidence level used: 0.95
## Intervals are back-transformed from the sqrt scale
```

### 3.3.3 Checking model assumptions

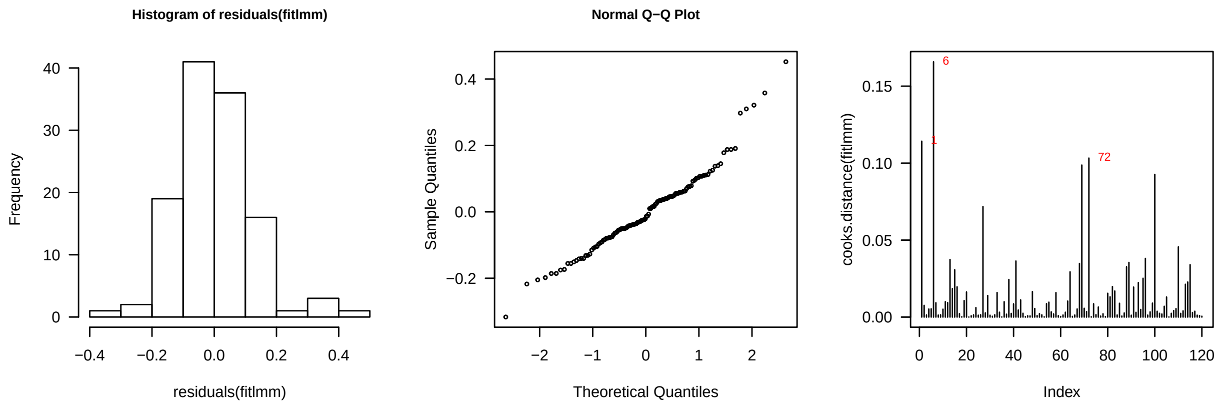


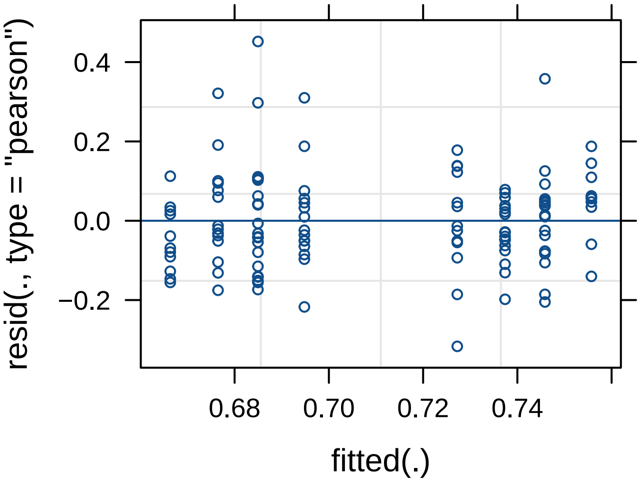


### 3.3.4 Conclusion

Linear mixed model indicated a significant difference between the two genotype (Type II Wald chi-square test; **p = 6.7e-3). Adj. Pvalue: *p = 0.027 (using Meff = 4.003 tests).

### 3.4 Forward swim BoutDuration [sqrt, *]

#### 3.4.1 Data visualization with clutch info

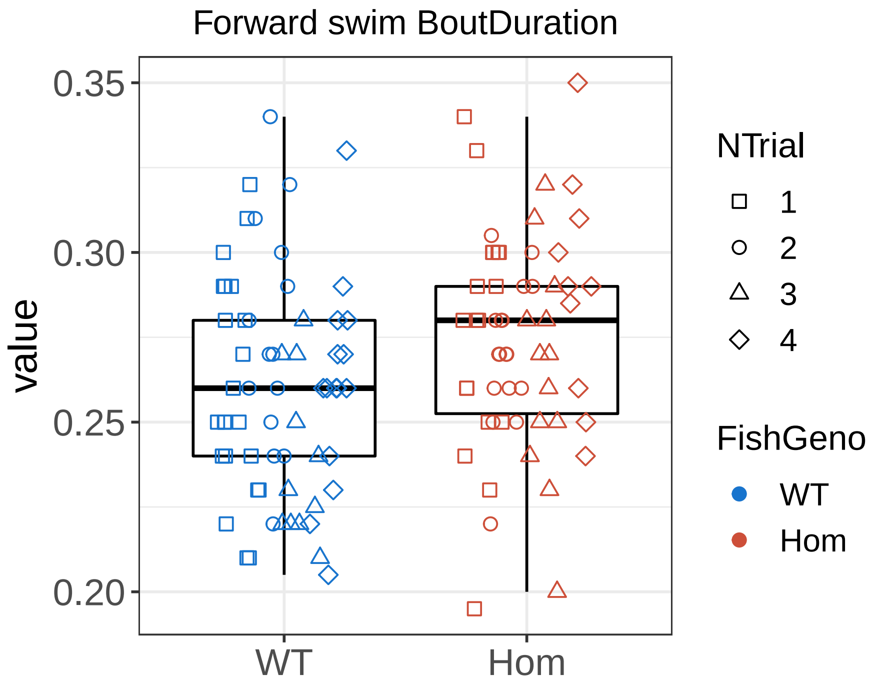


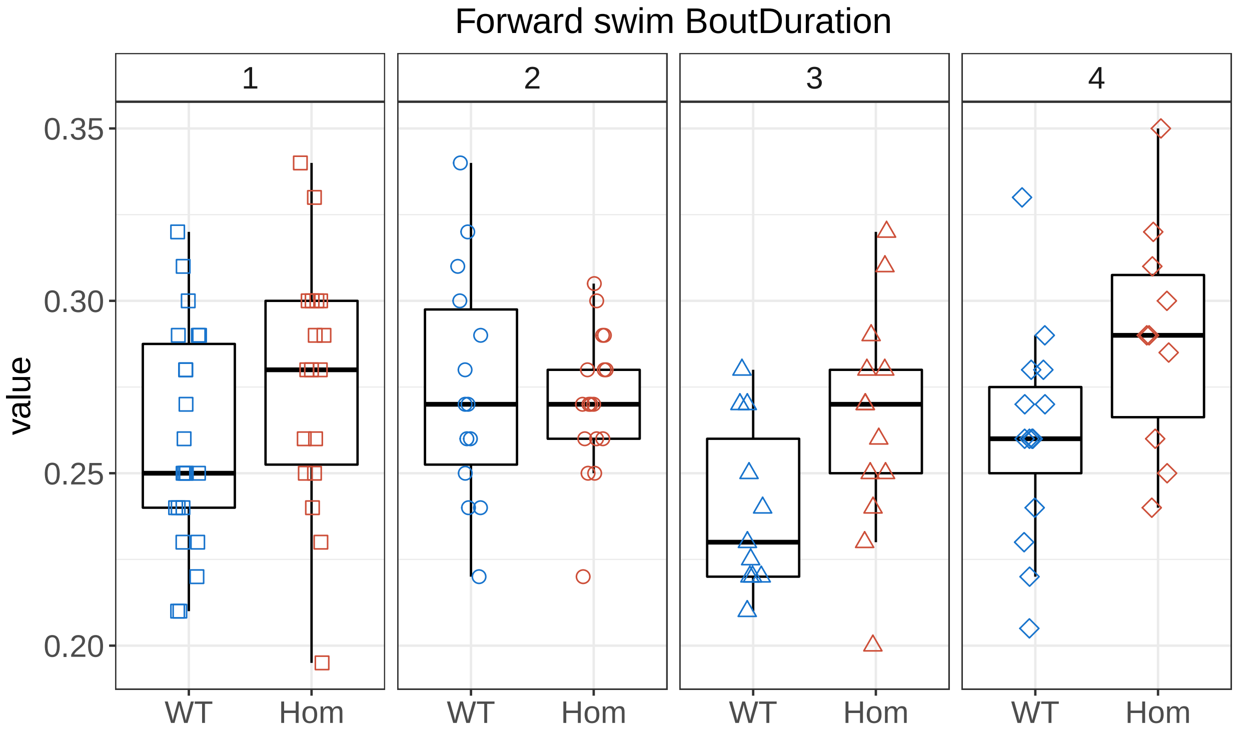

Table 4: Forward swim BoutDuration - Descriptive statistics

| | Min. | 1st Qu. | Median | Mean | 3rd Qu. | Max. |
|---|---|---|---|---|---|---|
| Hom_1 | 0.20 | 0.25 | 0.28 | 0.28 | 0.30 | 0.34 |
| Hom_2 | 0.22 | 0.26 | 0.27 | 0.27 | 0.28 | 0.30 |
| Hom_3 | 0.20 | 0.25 | 0.27 | 0.27 | 0.28 | 0.32 |
| Hom_4 | 0.24 | 0.27 | 0.29 | 0.29 | 0.31 | 0.35 |
| WT_1 | 0.21 | 0.24 | 0.25 | 0.26 | 0.29 | 0.32 |
| WT_2 | 0.22 | 0.25 | 0.27 | 0.28 | 0.30 | 0.34 |
| WT_3 | 0.21 | 0.22 | 0.23 | 0.24 | 0.26 | 0.28 |
| WT_4 | 0.20 | 0.25 | 0.26 | 0.26 | 0.28 | 0.33 |

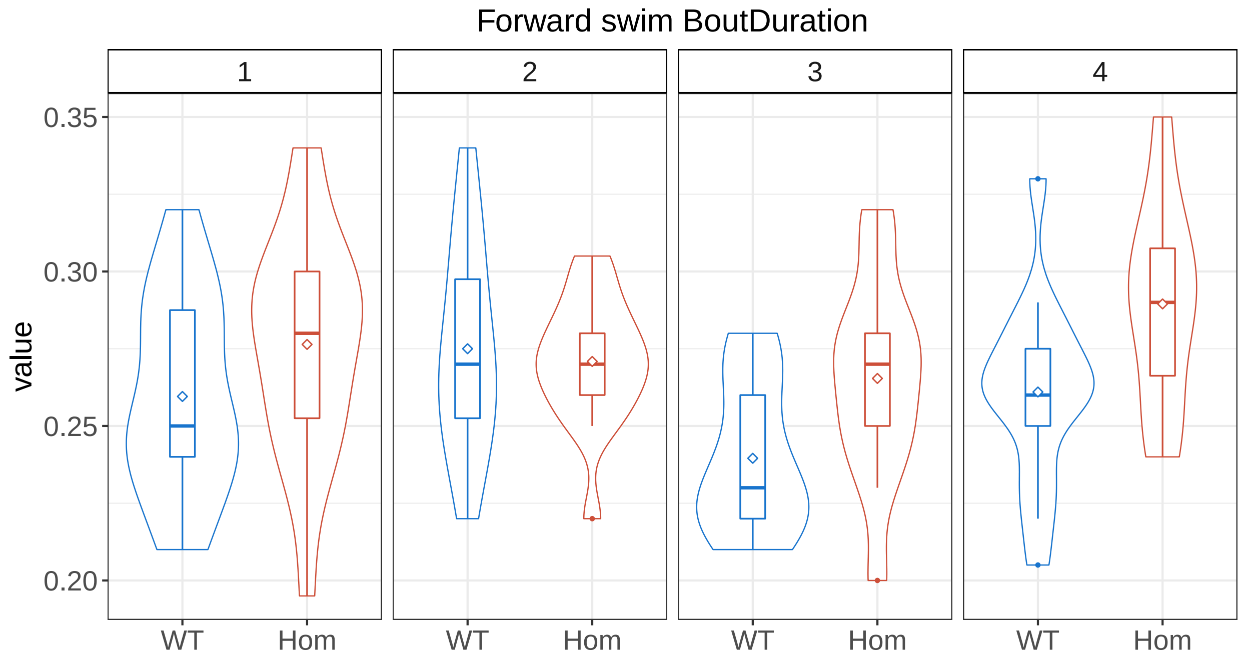

### 3.4.2 Fitting LMM

```
## Linear mixed model fit by REML ['lmerMod']
## Formula: sqrt(value) ~ FishGeno + (1 | NTrial)
##    Data: tmp
## REML criterion at convergence: -481.1822
## Random effects:
##  Groups   Name        Std.Dev.
##  NTrial   (Intercept) 0.006721
##  Residual             0.030078
## Number of obs: 120, groups:  NTrial, 4
## Fixed Effects:
## (Intercept)  FishGenoHom
##     0.50831      0.01478
```

```
## Analysis of Deviance Table (Type II Wald chisquare tests)
##
## Response: sqrt(value)
##           Chisq Df Pr(>Chisq)
## FishGeno 7.1709  1   0.007409 **
## ---
## Signif. codes:  0 '***' 0.001 '**' 0.01 '*' 0.05 '.' 0.1 ' ' 1
```

Estimated marginal means with model-based standard errors and 95% confidence limits on the original scale (non-transformed data):

```
## 'emmGrid' object with variables:
##     FishGeno = WT, Hom
## Transformation: "sqrt"
```

```
##  FishGeno response      SE   df lower.CL upper.CL
##  WT          0.258 0.00523 5.53    0.245    0.272
##  Hom         0.274 0.00547 5.94    0.260    0.287
##
## Degrees-of-freedom method: kenward-roger
## Confidence level used: 0.95
## Intervals are back-transformed from the sqrt scale
```

### 3.4.3 Checking model assumptions

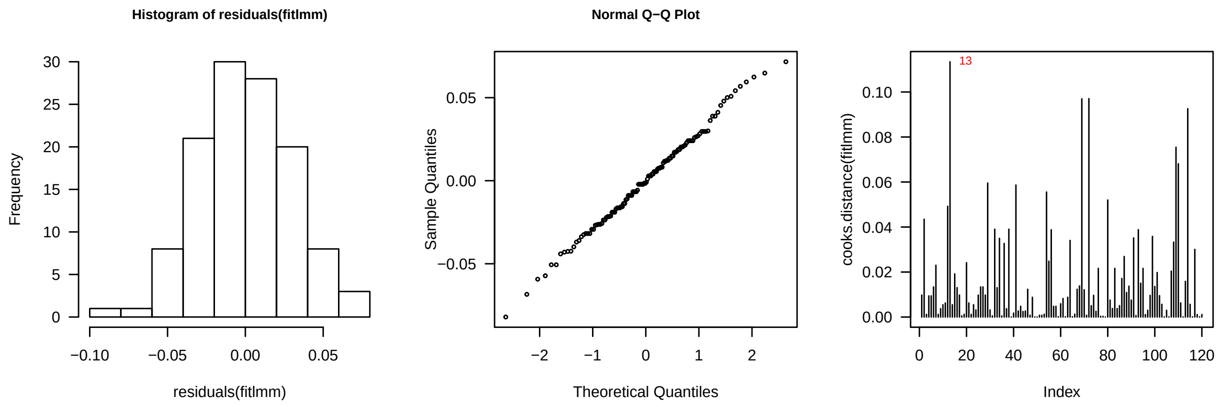


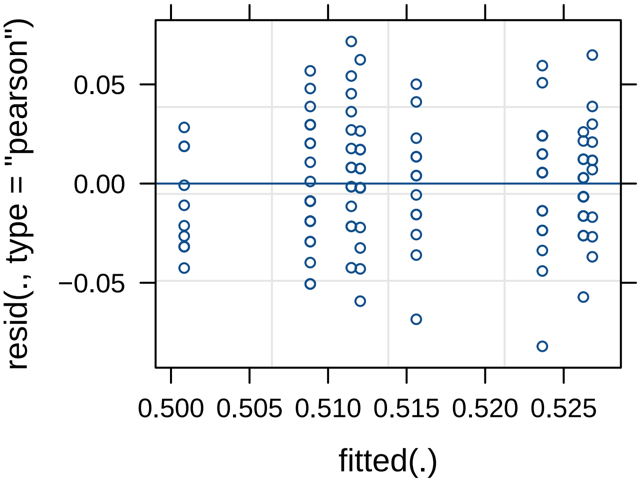


### 3.4.4 Conclusion

Linear mixed model indicated a significant difference between the two genotypes (Type II Wald chi-square test; **p = 7.4e-3). Adj. Pvalue: *p = 0.030 (using Meff = 4.003 tests).

### 3.5 Forward swim MedianBendAmp [log, *]

#### 3.5.1 Data visualization with clutch info

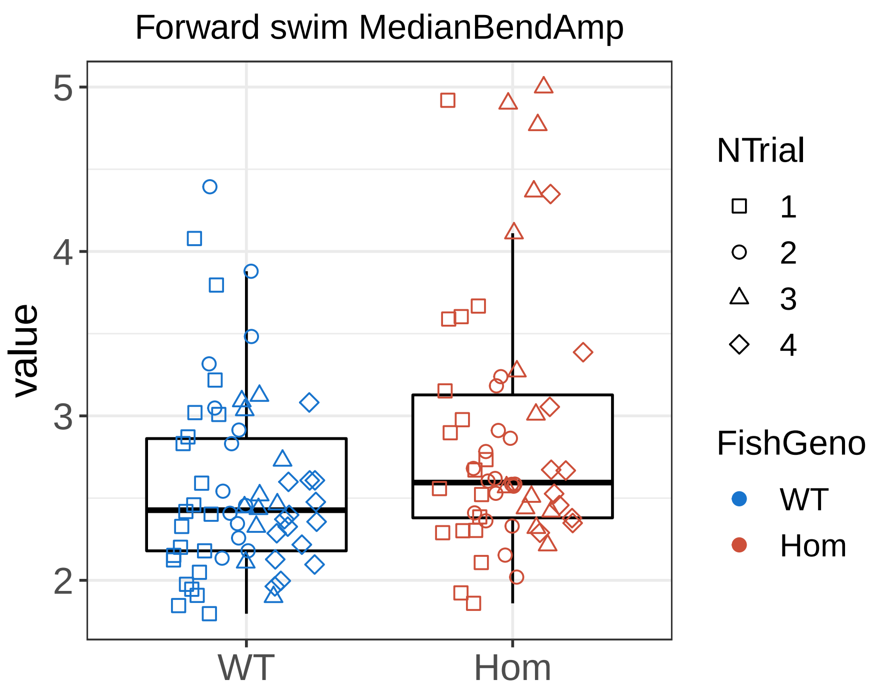


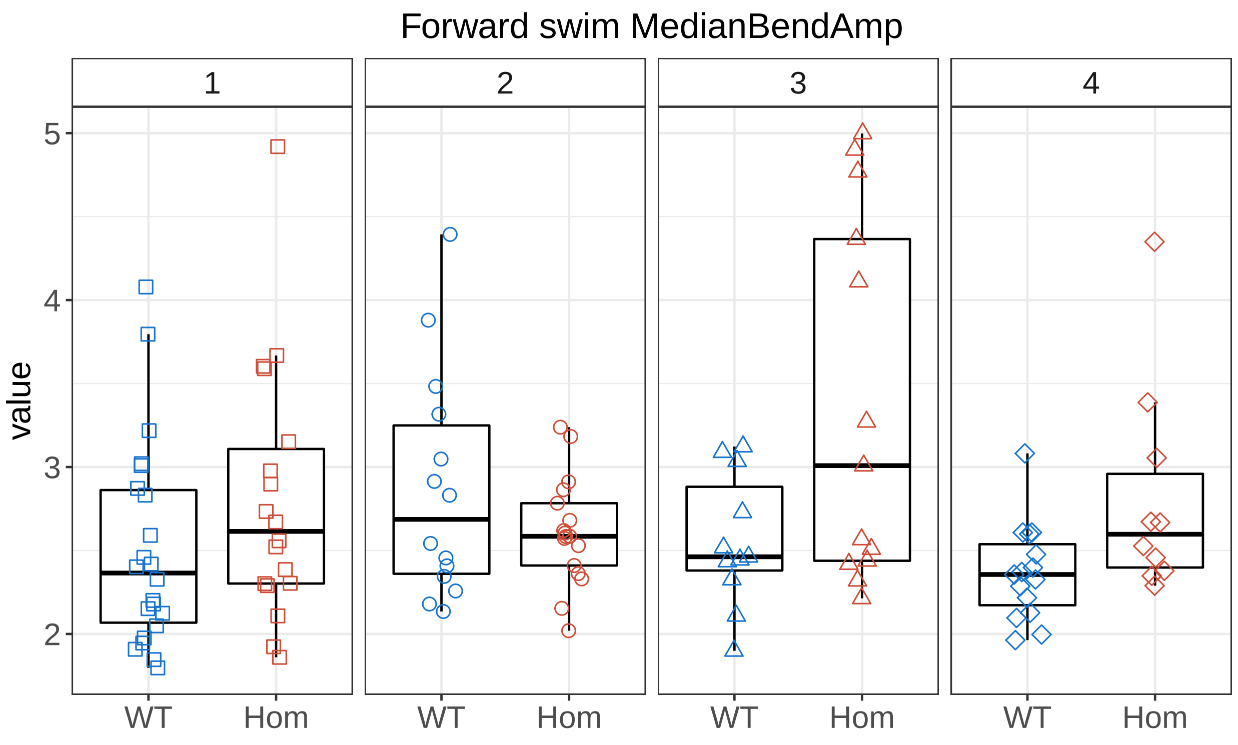

Table 5: Forward swim MedianBendAmp - Descriptive statistics

| | Min. | 1st Qu. | Median | Mean | 3rd Qu. | Max. |
|---|---|---|---|---|---|---|
| Hom_1 | 1.86 | 2.30 | 2.61 | 2.80 | 3.11 | 4.92 |
| Hom_2 | 2.02 | 2.41 | 2.58 | 2.61 | 2.78 | 3.24 |
| Hom_3 | 2.21 | 2.44 | 3.01 | 3.38 | 4.37 | 5.00 |
| Hom_4 | 2.29 | 2.40 | 2.60 | 2.81 | 2.96 | 4.35 |
| WT_1 | 1.80 | 2.07 | 2.36 | 2.51 | 2.86 | 4.08 |
| WT_2 | 2.13 | 2.36 | 2.69 | 2.87 | 3.25 | 4.39 |
| WT_3 | 1.90 | 2.38 | 2.46 | 2.56 | 2.88 | 3.12 |
| WT_4 | 1.96 | 2.17 | 2.36 | 2.37 | 2.54 | 3.08 |

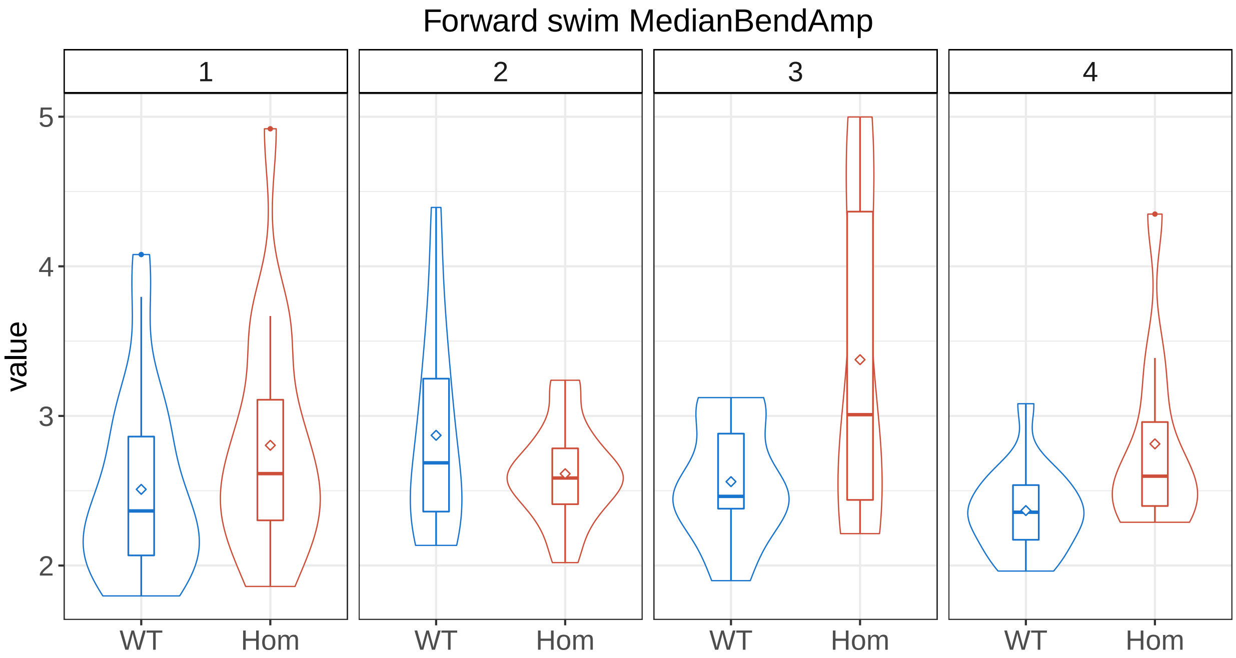

### 3.5.2 Fitting LMM

```
## Linear mixed model fit by REML ['lmerMod']
## Formula: log(value) ~ FishGeno + (1 | NTrial)
##    Data: tmp
## REML criterion at convergence: -14.7918
## Random effects:
##  Groups   Name        Std.Dev.
##  NTrial   (Intercept) 0.03226
##  Residual             0.21814
## Number of obs: 120, groups:  NTrial, 4
## Fixed Effects:
## (Intercept)  FishGenoHom
##      0.9249       0.1021
```

```
## Analysis of Deviance Table (Type II Wald chisquare tests)
##
## Response: log(value)
##           Chisq Df Pr(>Chisq)
## FishGeno 6.5288  1    0.01061 *
## ---
## Signif. codes:  0 '***' 0.001 '**' 0.01 '*' 0.05 '.' 0.1 ' ' 1
```

Estimated marginal means with model-based standard errors and 95% confidence limits on the original scale (non-transformed data):

```
## 'emmGrid' object with variables:
##     FishGeno = WT, Hom
## Transformation: "log"
```

```
##  FishGeno response     SE   df lower.CL upper.CL
##  WT           2.52 0.0821 6.92     2.33     2.72
##  Hom          2.79 0.0928 7.66     2.58     3.02
##
## Degrees-of-freedom method: kenward-roger
## Confidence level used: 0.95
## Intervals are back-transformed from the log scale
```

### 3.5.3 Checking model assumptions

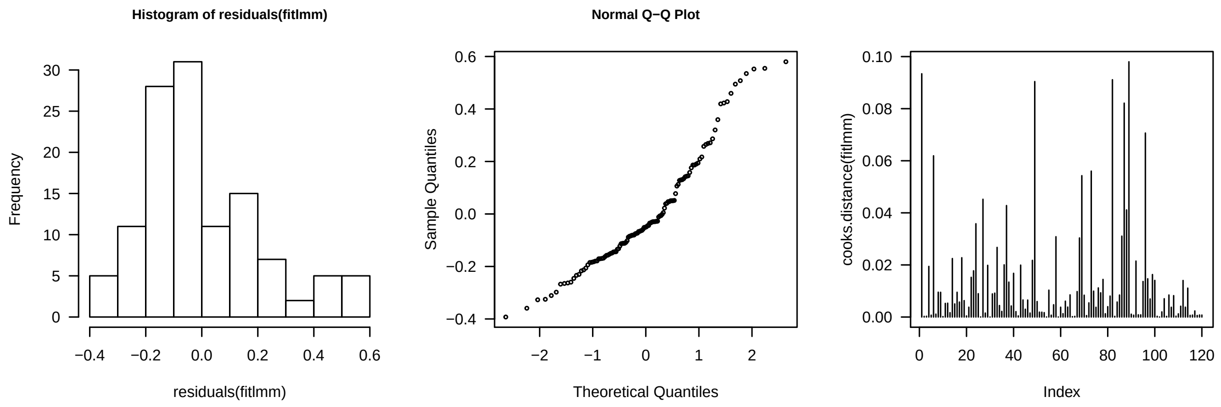


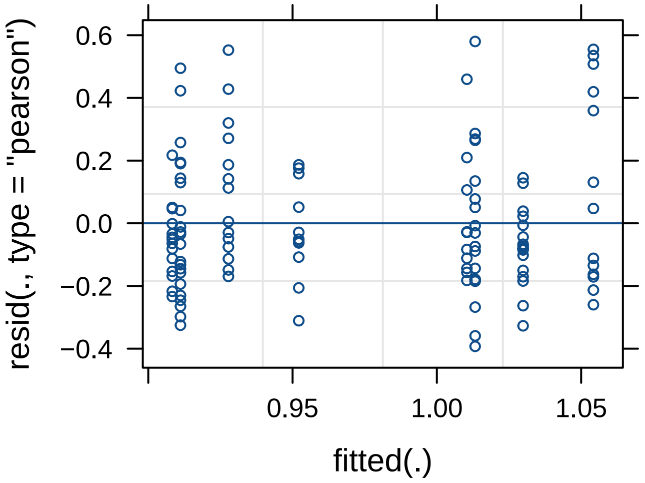


### 3.5.4 Conclusion

Linear mixed model indicated a significant difference between the two genotypes (Type II Wald chi-square test; *p = 0.011). Adj. Pvalue: *p = 0.042 (using Meff = 4.003 tests).

## 3.6 Forward swim NumOfOsc [ns]

### 3.6.1 Data visualization with clutch info

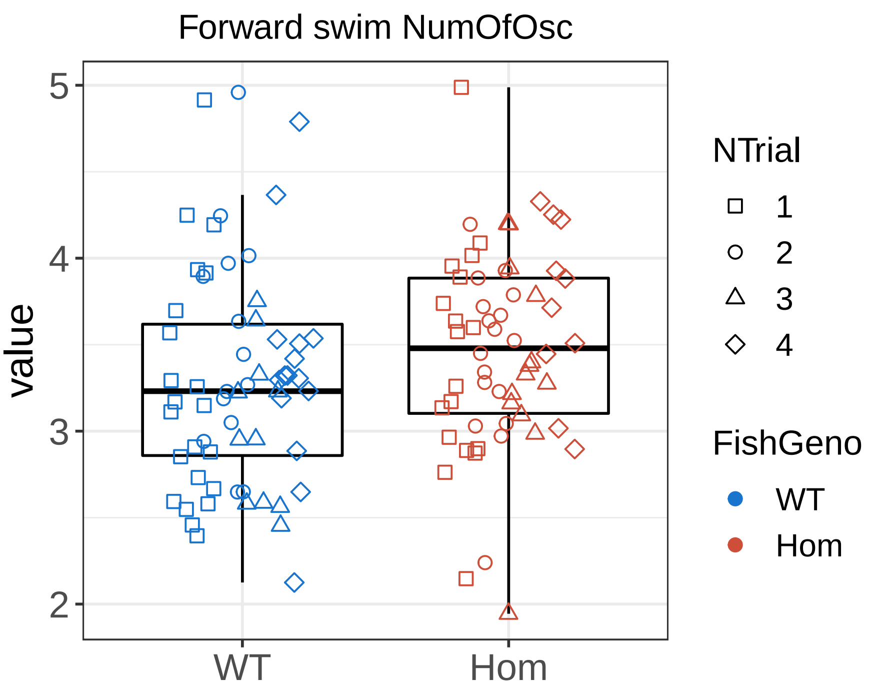


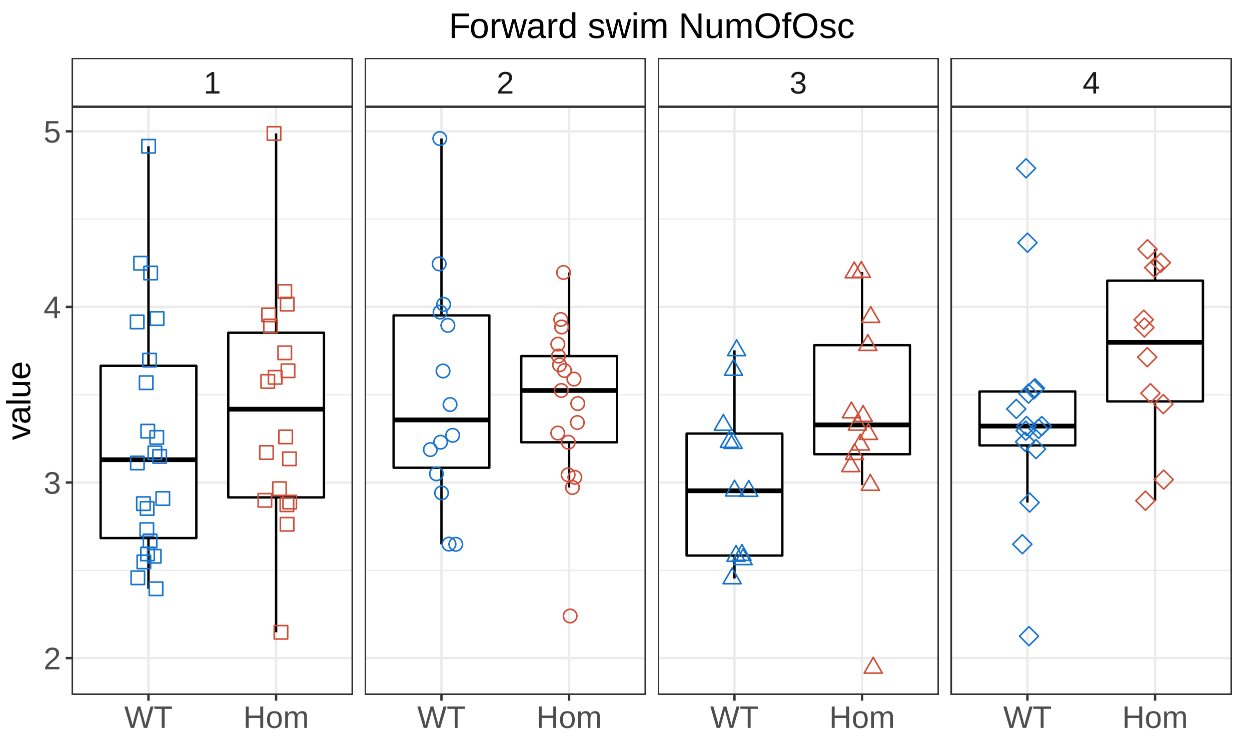

Table 6: Forward swim NumOfOsc - Descriptive statistics

| | Min. | 1st Qu. | Median | Mean | 3rd Qu. | Max. |
|---|---|---|---|---|---|---|
| Hom_1 | 2.15 | 2.92 | 3.42 | 3.42 | 3.85 | 4.99 |
| Hom_2 | 2.24 | 3.23 | 3.52 | 3.44 | 3.72 | 4.20 |
| Hom_3 | 1.94 | 3.16 | 3.33 | 3.38 | 3.78 | 4.20 |
| Hom_4 | 2.90 | 3.46 | 3.80 | 3.72 | 4.15 | 4.33 |
| WT_1 | 2.40 | 2.68 | 3.13 | 3.23 | 3.66 | 4.92 |
| WT_2 | 2.65 | 3.08 | 3.36 | 3.51 | 3.95 | 4.96 |
| WT_3 | 2.45 | 2.58 | 2.95 | 3.02 | 3.28 | 3.75 |
| WT_4 | 2.12 | 3.21 | 3.32 | 3.37 | 3.52 | 4.79 |

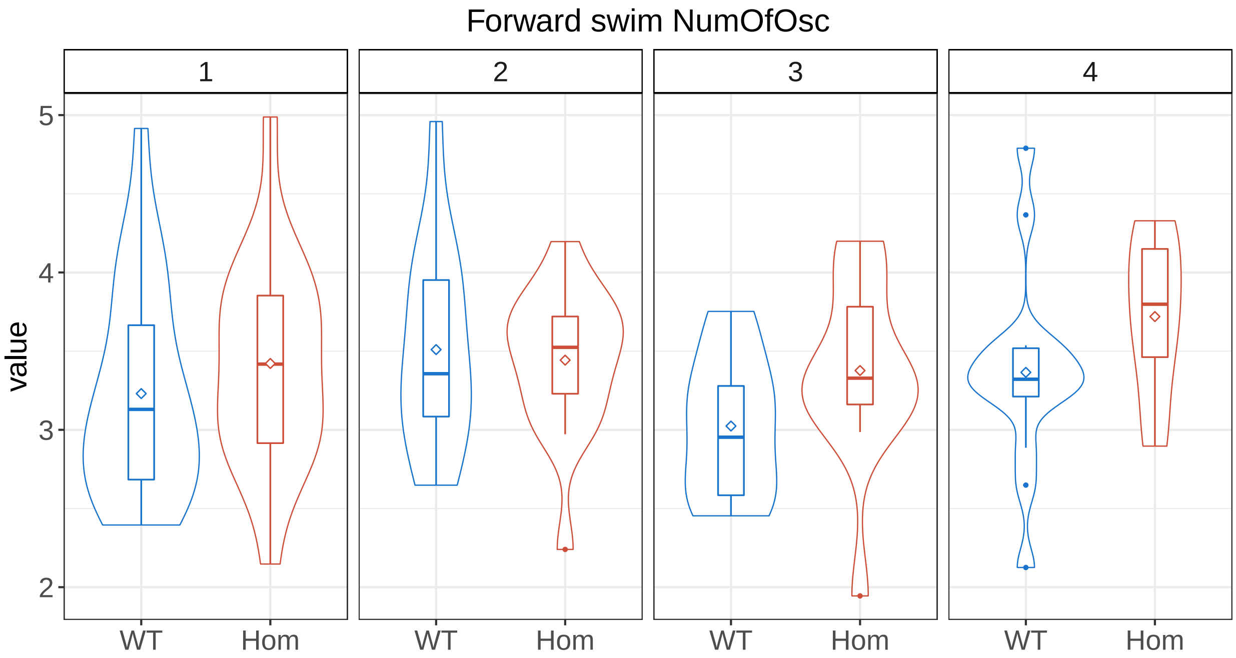

### 3.6.2 Fitting LMM

```
## Linear mixed model fit by REML ['lmerMod']
## Formula: value ~ FishGeno + (1 | NTrial)
##    Data: tmp
## REML criterion at convergence: 221.5206
## Random effects:
##  Groups   Name        Std.Dev.
##  NTrial   (Intercept) 0.07107
##  Residual             0.59486
## Number of obs: 120, groups:  NTrial, 4
## Fixed Effects:
## (Intercept)  FishGenoHom
##      3.2892       0.1819
```

```
## Analysis of Deviance Table (Type II Wald chisquare tests)
##
## Response: value
##          Chisq Df Pr(>Chisq)
## FishGeno 2.789  1    0.09491 .
## ---
## Signif. codes:  0 '***' 0.001 '**' 0.01 '*' 0.05 '.' 0.1 ' ' 1
```

Estimated marginal means with model-based standard errors and 95% confidence limits on the original scale (non-transformed data):

```
## 'emmGrid' object with variables:
##     FishGeno = WT, Hom
```

```
##  FishGeno emmean     SE   df lower.CL upper.CL
##  WT         3.29 0.0850 7.58     3.09     3.49
##  Hom        3.47 0.0869 8.51     3.27     3.67
##
## Degrees-of-freedom method: kenward-roger
## Confidence level used: 0.95
```

### 3.6.3 Checking model assumptions

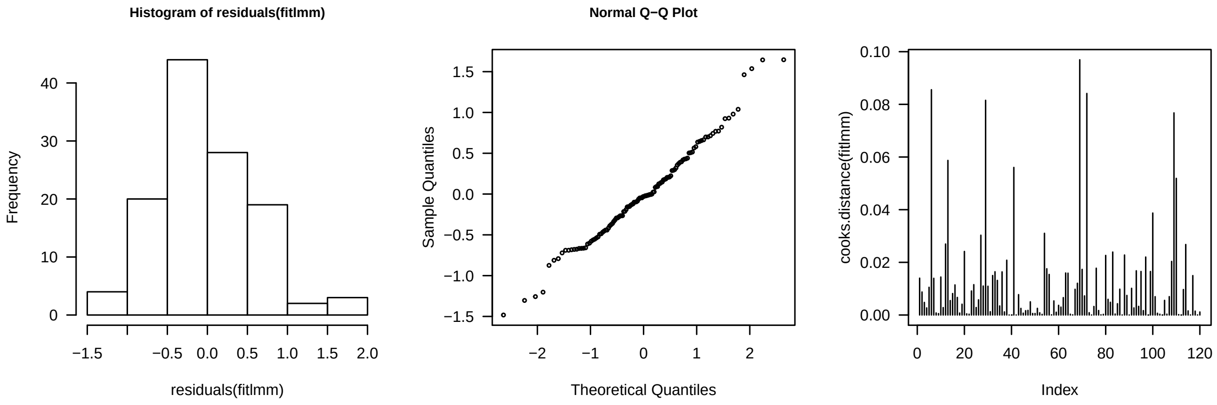


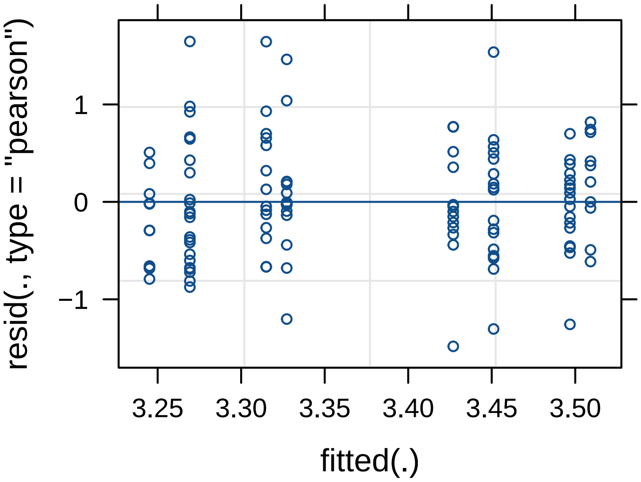


### 3.6.4 Conclusion

No significant difference was detected between the two groups (Type II Wald chi-square test; $^{ns}$p = 0.094). Adj. Pvalue: $^{ns}$p = 0.380 (using Meff = 4.003 tests).

## 3.7 Forward swim Speed [log, *]

### 3.7.1 Data visualization with clutch info

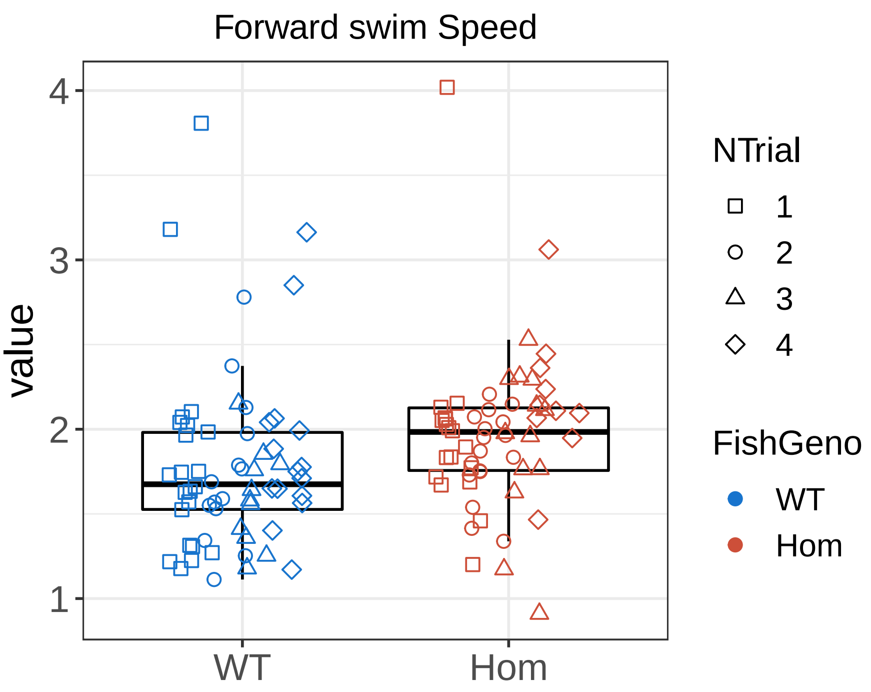


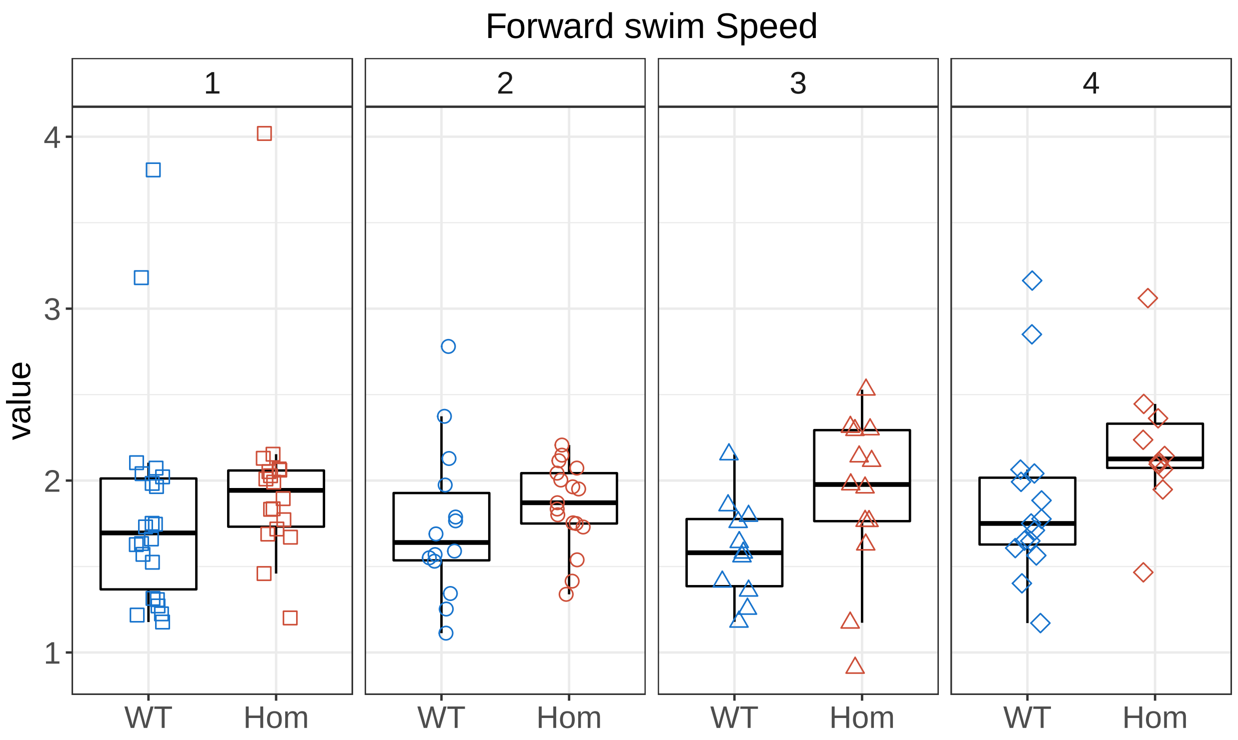

Table 7: Forward swim Speed - Descriptive statistics

| | Min. | 1st Qu. | Median | Mean | 3rd Qu. | Max. |
|---|---|---|---|---|---|---|
| Hom_1 | 1.20 | 1.73 | 1.94 | 1.98 | 2.06 | 4.02 |
| Hom_2 | 1.34 | 1.75 | 1.87 | 1.86 | 2.04 | 2.21 |
| Hom_3 | 0.91 | 1.76 | 1.98 | 1.91 | 2.29 | 2.53 |
| Hom_4 | 1.47 | 2.07 | 2.13 | 2.19 | 2.33 | 3.06 |
| WT_1 | 1.18 | 1.37 | 1.70 | 1.82 | 2.01 | 3.81 |
| WT_2 | 1.11 | 1.54 | 1.64 | 1.75 | 1.93 | 2.78 |
| WT_3 | 1.18 | 1.39 | 1.58 | 1.59 | 1.78 | 2.15 |
| WT_4 | 1.17 | 1.63 | 1.75 | 1.89 | 2.02 | 3.16 |

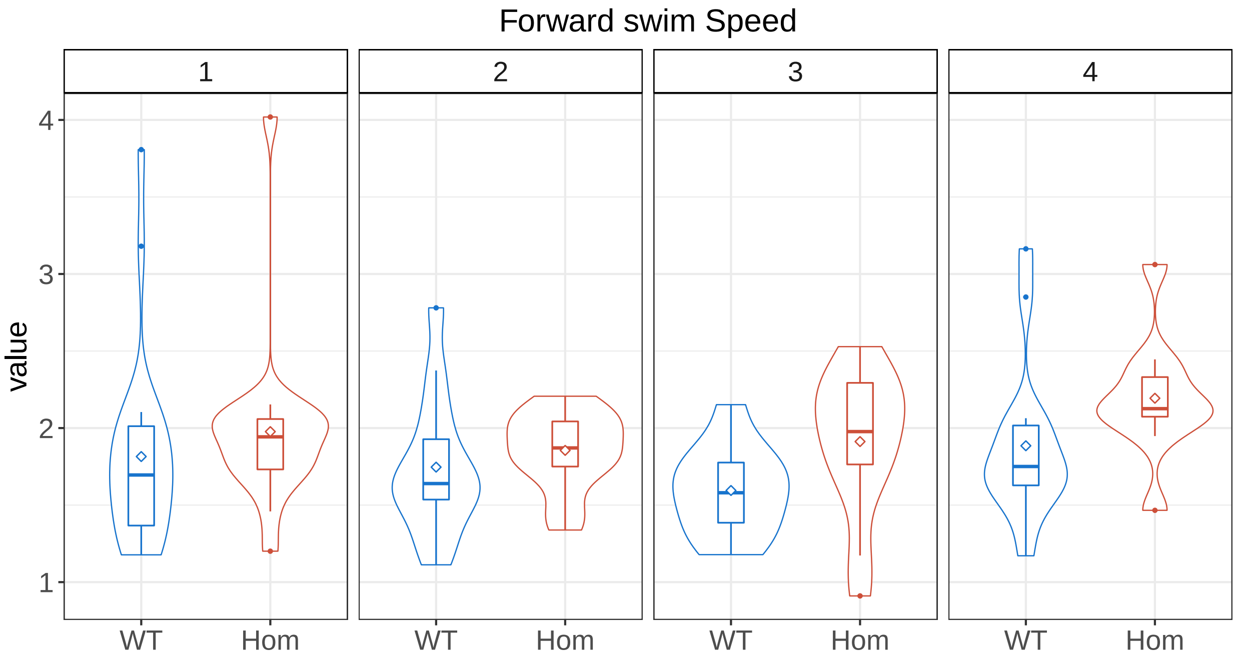

### 3.7.2 Fitting LMM

```
## Linear mixed model fit by REML ['lmerMod']
## Formula: log(value) ~ FishGeno + (1 | NTrial)
##    Data: tmp
## REML criterion at convergence: 5.0714
## Random effects:
##  Groups   Name        Std.Dev.
##  NTrial   (Intercept) 0.03756
##  Residual             0.23713
## Number of obs: 120, groups:  NTrial, 4
## Fixed Effects:
## (Intercept)  FishGenoHom
##      0.5389       0.1146
```

```
## Analysis of Deviance Table (Type II Wald chisquare tests)
##
## Response: log(value)
##           Chisq Df Pr(>Chisq)
## FishGeno 6.9551  1   0.008358 **
## ---
## Signif. codes:  0 '***' 0.001 '**' 0.01 '*' 0.05 '.' 0.1 ' ' 1
```

Estimated marginal means with model-based standard errors and 95% confidence limits on the original scale (non-transformed data):

```
## 'emmGrid' object with variables:
##     FishGeno = WT, Hom
## Transformation: "log"
```

```
##  FishGeno response     SE   df lower.CL upper.CL
##  WT           1.71 0.0617 6.69     1.57     1.87
##  Hom          1.92 0.0706 7.37     1.76     2.09
##
## Degrees-of-freedom method: kenward-roger
## Confidence level used: 0.95
## Intervals are back-transformed from the log scale
```

### 3.7.3 Checking model assumptions

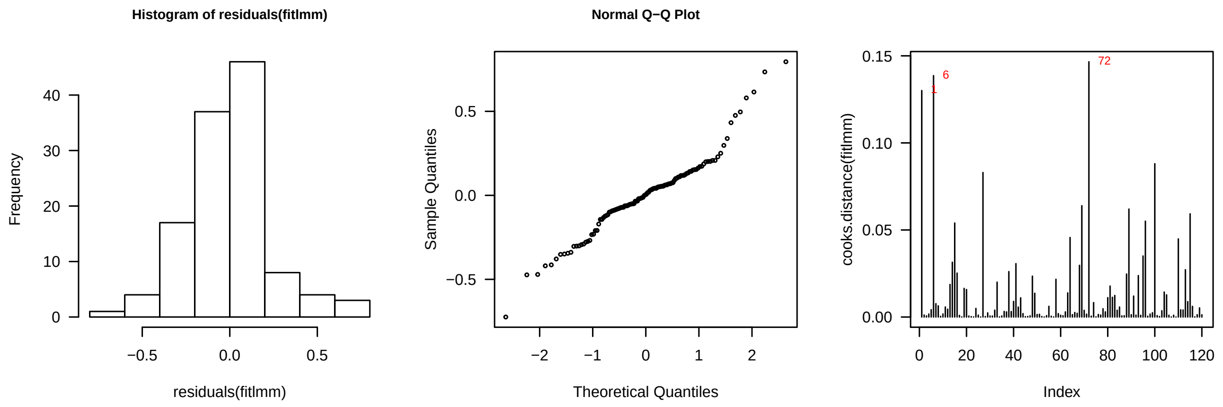


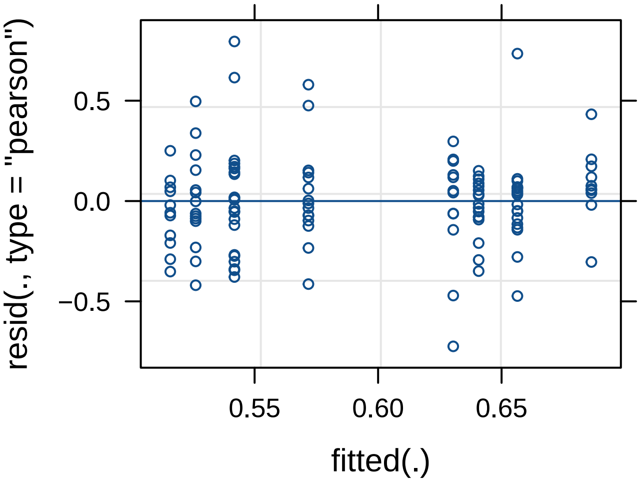


### 3.7.4 Conclusion

Linear mixed model indicated a significant difference between the two genotypes (Type II Wald chi-square test; *p = 8.4e-3). Adj. Pvalue: *p = 0.033 (using Meff = 4.003 tests).

## 3.8 Forward swim TBF [ns]

### 3.8.1 Data visualization with clutch info

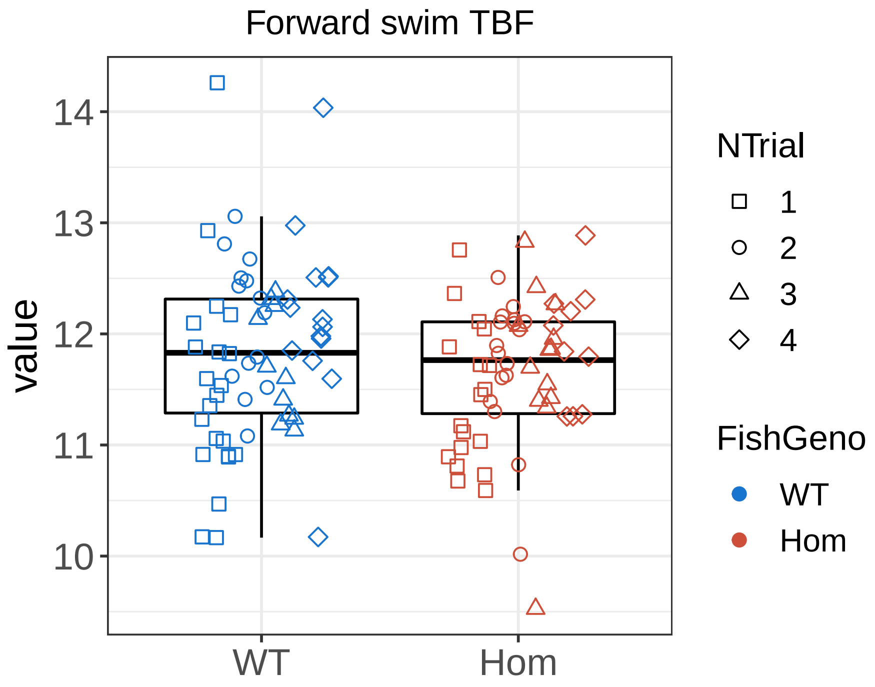


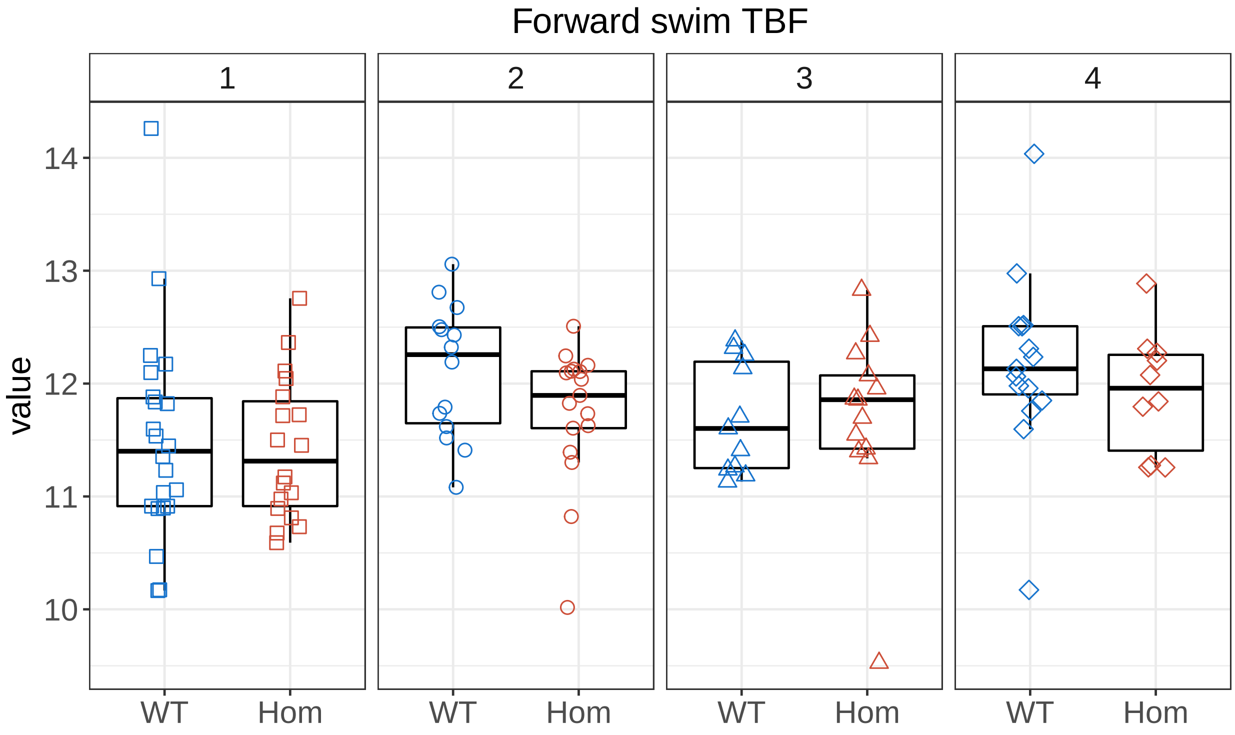

Table 8: Forward swim TBF - Descriptive statistics

| | Min. | 1st Qu. | Median | Mean | 3rd Qu. | Max. |
|---|---|---|---|---|---|---|
| Hom_1 | 10.59 | 10.91 | 11.31 | 11.42 | 11.84 | 12.76 |
| Hom_2 | 10.02 | 11.61 | 11.90 | 11.74 | 12.11 | 12.51 |
| Hom_3 | 9.53 | 11.42 | 11.86 | 11.71 | 12.07 | 12.83 |
| Hom_4 | 11.26 | 11.41 | 11.96 | 11.92 | 12.25 | 12.89 |
| WT_1 | 10.17 | 10.91 | 11.40 | 11.50 | 11.87 | 14.26 |
| WT_2 | 11.08 | 11.65 | 12.26 | 12.12 | 12.50 | 13.06 |
| WT_3 | 11.13 | 11.25 | 11.60 | 11.69 | 12.19 | 12.38 |
| WT_4 | 10.17 | 11.90 | 12.13 | 12.17 | 12.51 | 14.04 |

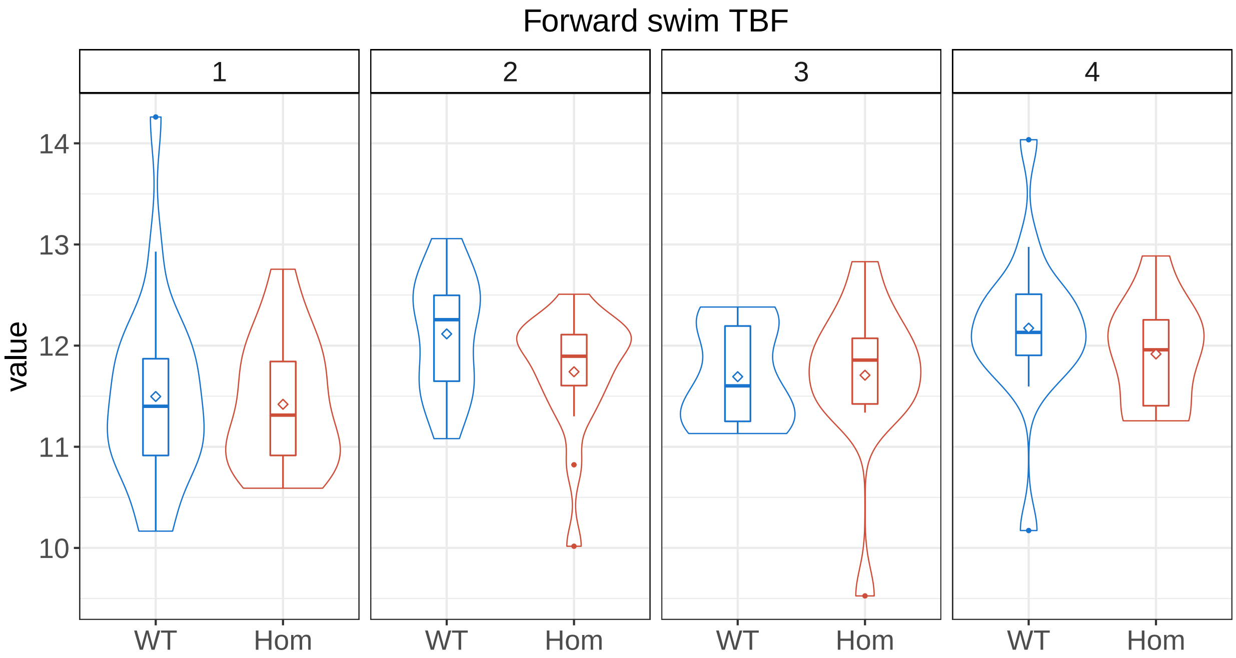

### 3.8.2 Fitting LMM

```
## Linear mixed model fit by REML ['lmerMod']
## Formula: value ~ FishGeno + (1 | NTrial)
##    Data: tmp
## REML criterion at convergence: 265.3554
## Random effects:
##  Groups   Name        Std.Dev.
##  NTrial   (Intercept) 0.2342
##  Residual             0.7065
## Number of obs: 120, groups:  NTrial, 4
## Fixed Effects:
## (Intercept)  FishGenoHom
##     11.8634      -0.1733
```

```
## Analysis of Deviance Table (Type II Wald chisquare tests)
##
## Response: value
##           Chisq Df Pr(>Chisq)
## FishGeno 1.7846  1     0.1816
```

Estimated marginal means with model-based standard errors and 95% confidence limits on the original scale (non-transformed data):

```
## 'emmGrid' object with variables:
##     FishGeno = WT, Hom
```

```
##  FishGeno emmean    SE   df lower.CL upper.CL
##  WT         11.9 0.148 4.42     11.5     12.3
##  Hom        11.7 0.150 4.63     11.3     12.1
##
## Degrees-of-freedom method: kenward-roger
## Confidence level used: 0.95
```

### 3.8.3 Checking model assumptions

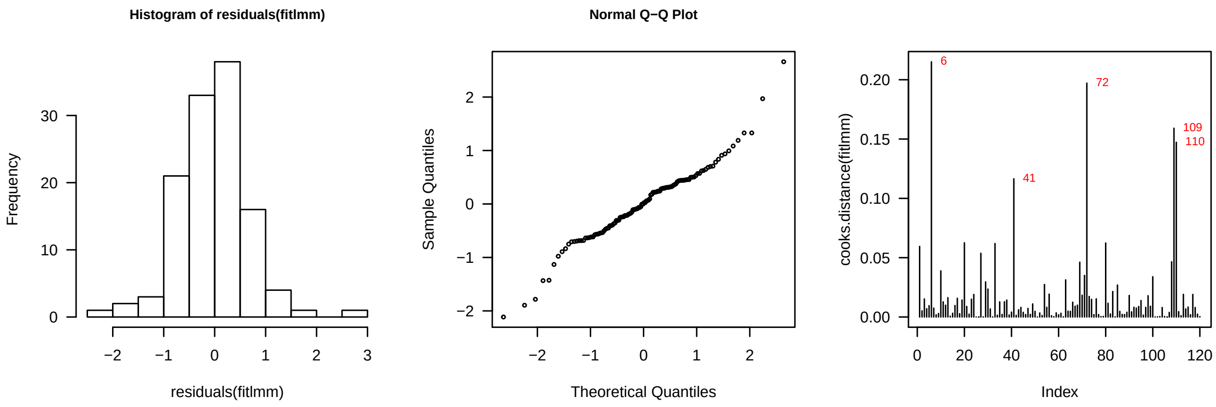


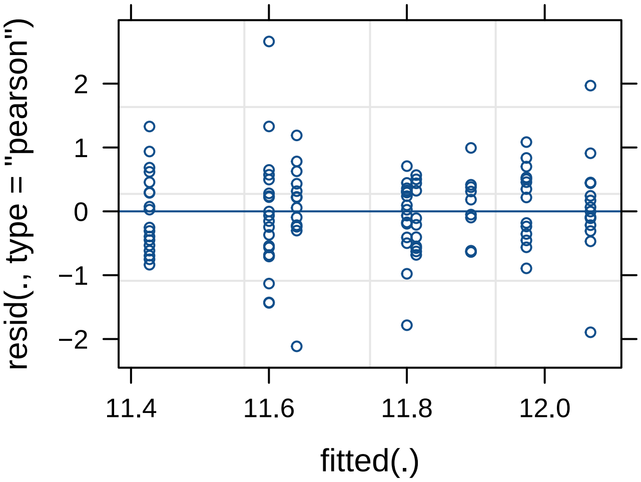


### 3.8.4 Conclusion

No significant difference was detected between the two groups (Type II Wald chi-square test; ${}^{ns}$p = 0.182). Adj. Pvalue: ${}^{ns}$p = 0.727 (using Meff = 4.003 tests).

# 4 Routine turn

## 4.1 Descriptive statistics

Table 9: Routine turn WT - Mean values by clutch

| | WT_1 | WT_2 | WT_3 | WT_4 | MEAN |
|---|---|---|---|---|---|
| BoutDuration | 0.40 | 0.38 | 0.34 | 0.36 | 0.37 |
| NumberOfOscillations | 5.67 | 5.06 | 4.40 | 4.88 | 5.00 |
| BoutDistance | 1.59 | 1.53 | 1.23 | 1.50 | 1.46 |
| Speed | 3.91 | 3.85 | 3.47 | 4.10 | 3.83 |
| TBF | 11.78 | 11.72 | 11.69 | 12.15 | 11.84 |
| MedianBendAmp | 4.43 | 5.14 | 5.25 | 3.88 | 4.67 |

Table 10: Routine turn Hom - Mean values by clutch

| | Hom_1 | Hom_2 | Hom_3 | Hom_4 | MEAN |
|---|---|---|---|---|---|
| BoutDuration | 0.41 | 0.38 | 0.40 | 0.39 | 0.40 |
| NumberOfOscillations | 5.45 | 4.85 | 6.37 | 5.10 | 5.44 |
| BoutDistance | 1.61 | 1.48 | 1.61 | 1.58 | 1.57 |
| Speed | 3.86 | 3.80 | 3.84 | 3.98 | 3.87 |
| TBF | 11.54 | 11.40 | 12.17 | 11.77 | 11.72 |
| MedianBendAmp | 4.66 | 5.09 | 5.23 | 4.41 | 4.85 |

## 4.2 All Escape correlation matrix

Using Spearman's rank correlations:

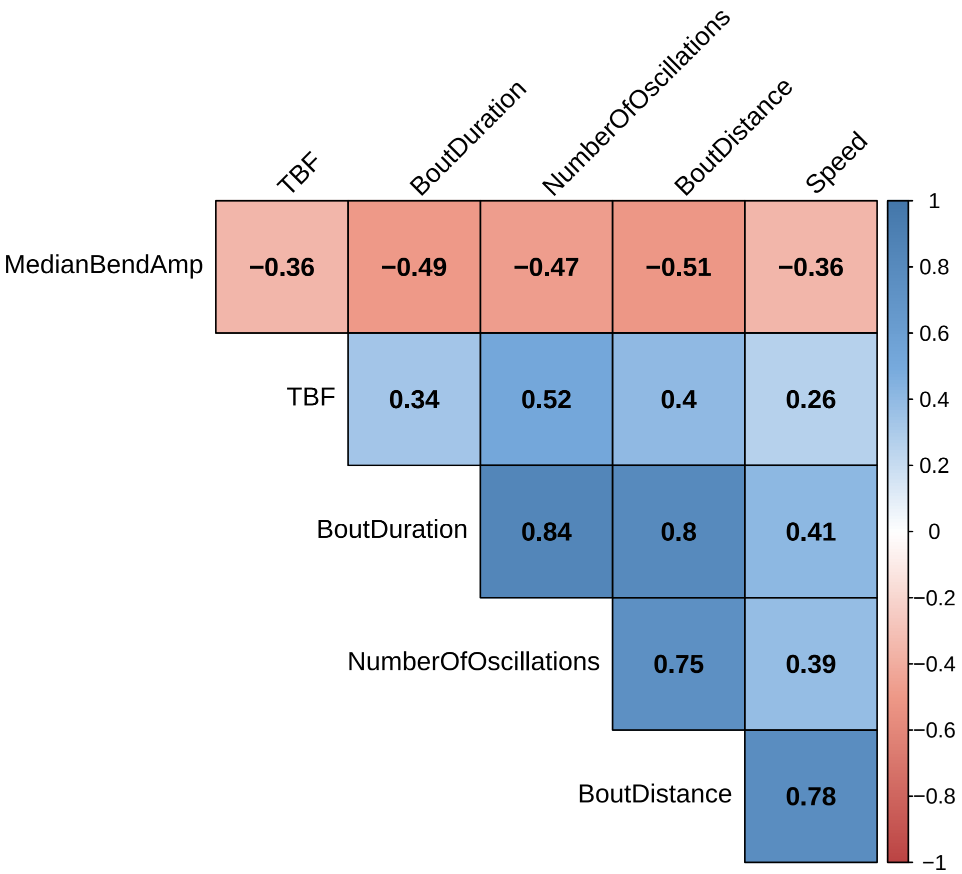


Based on 6 kinematic parameters, variance of eigenvalues of the correlation matrix is 1.778 leading to a Meff value of 4.518 to be used for multiple testing correction.

## 4.3 Routine turn BoutDistance [sqrt, ns]

### 4.3.1 Data visualization with clutch info

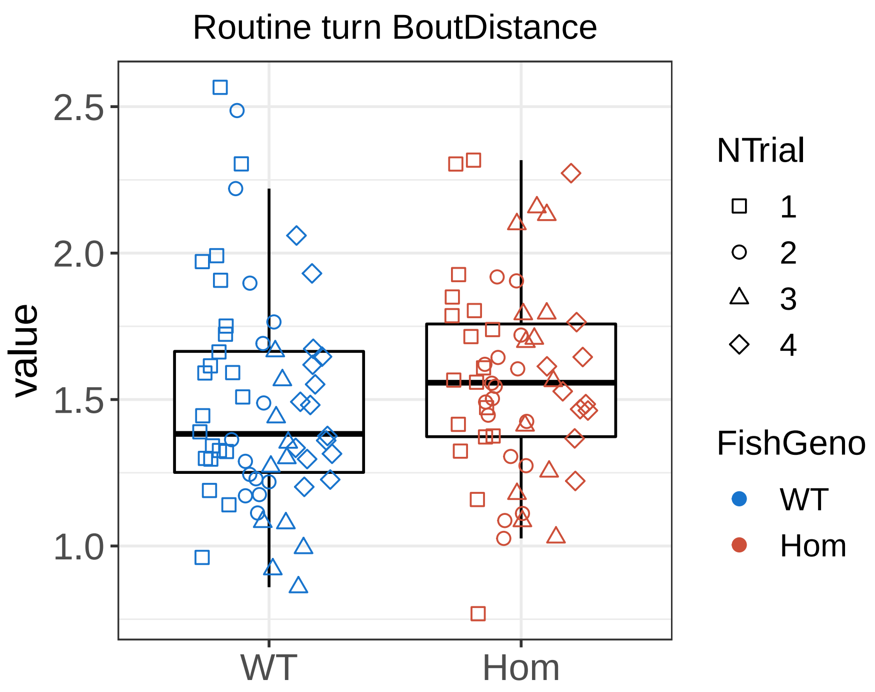


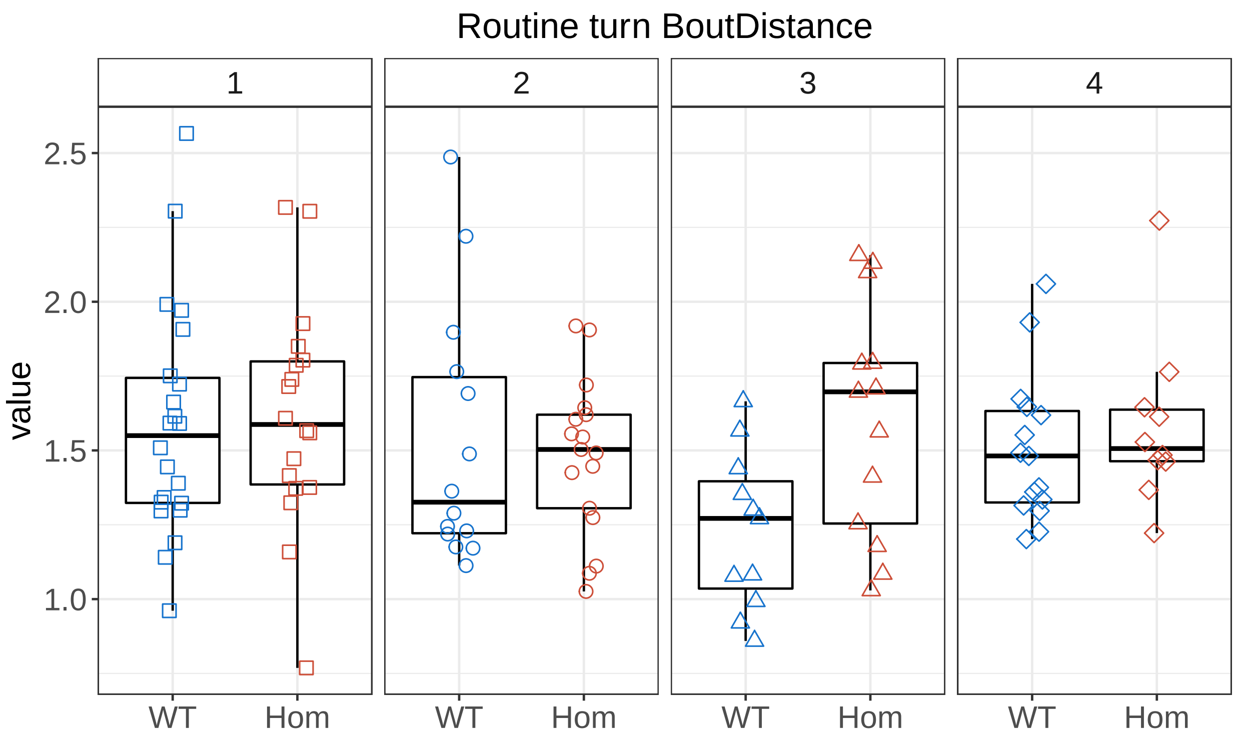

Table 11: Routine turn BoutDistance - Descriptive statistics

| | Min. | 1st Qu. | Median | Mean | 3rd Qu. | Max. |
|---|---|---|---|---|---|---|
| Hom_1 | 0.77 | 1.39 | 1.59 | 1.61 | 1.80 | 2.32 |
| Hom_2 | 1.03 | 1.31 | 1.50 | 1.48 | 1.62 | 1.92 |
| Hom_3 | 1.03 | 1.25 | 1.70 | 1.61 | 1.79 | 2.16 |
| Hom_4 | 1.22 | 1.46 | 1.51 | 1.58 | 1.64 | 2.27 |
| WT_1 | 0.96 | 1.32 | 1.55 | 1.59 | 1.74 | 2.57 |
| WT_2 | 1.11 | 1.22 | 1.33 | 1.53 | 1.75 | 2.49 |
| WT_3 | 0.86 | 1.04 | 1.27 | 1.23 | 1.40 | 1.67 |
| WT_4 | 1.20 | 1.32 | 1.48 | 1.50 | 1.63 | 2.06 |

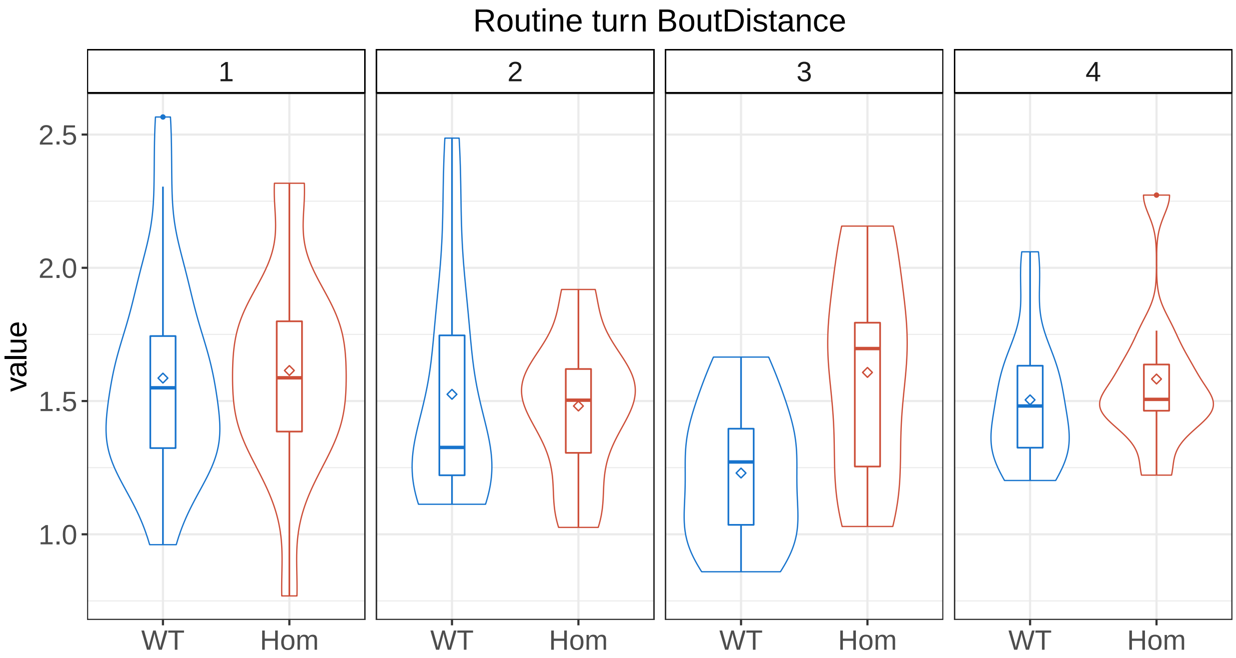

### 4.3.2 Fitting LMM

```
## Linear mixed model fit by REML ['lmerMod']
## Formula: sqrt(value) ~ FishGeno + (1 | NTrial)
##    Data: tmp
## REML criterion at convergence: -122.8113
## Random effects:
##  Groups   Name        Std.Dev.
##  NTrial   (Intercept) 0.01785
##  Residual             0.13820
## Number of obs: 120, groups:  NTrial, 4
## Fixed Effects:
## (Intercept)  FishGenoHom
##     1.20979      0.03497
```

```
## Analysis of Deviance Table (Type II Wald chisquare tests)
##
## Response: sqrt(value)
##           Chisq Df Pr(>Chisq)
## FishGeno 1.9098  1      0.167
```

Estimated marginal means with model-based standard errors and 95% confidence limits on the original scale (non-transformed data):

```
## 'emmGrid' object with variables:
##     FishGeno = WT, Hom
## Transformation: "sqrt"
```

```
##  FishGeno response     SE   df lower.CL upper.CL
##  WT           1.46 0.0485 7.35     1.35     1.58
##  Hom          1.55 0.0509 8.21     1.43     1.67
##
## Degrees-of-freedom method: kenward-roger
## Confidence level used: 0.95
## Intervals are back-transformed from the sqrt scale
```

### 4.3.3 Checking model assumptions

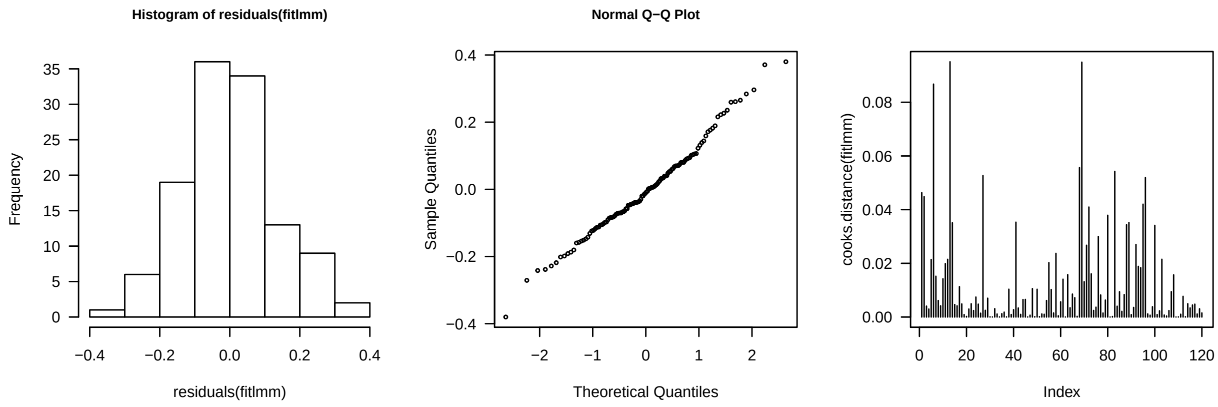


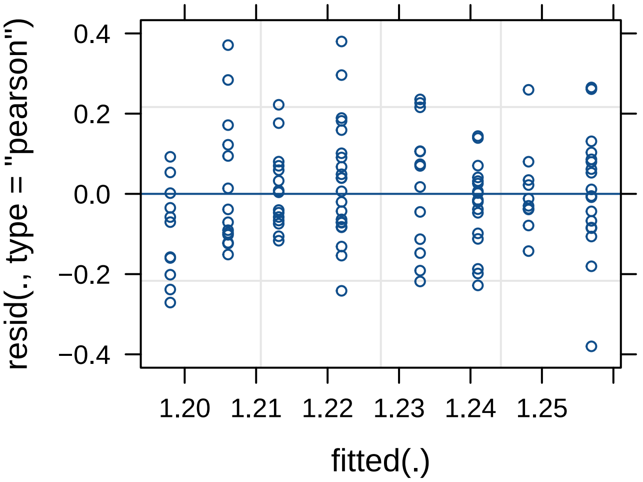


### 4.3.4 Conclusion

No significant difference was detected between the two groups (Type II Wald chi-square test; $^{ns}$p = 0.167). Adj. Pvalue: $^{ns}$p = 0.755 (using Meff = 4.518 tests).

## 4.4 Routine turn Duration [sqrt, ns]

### 4.4.1 Data visualization with clutch info

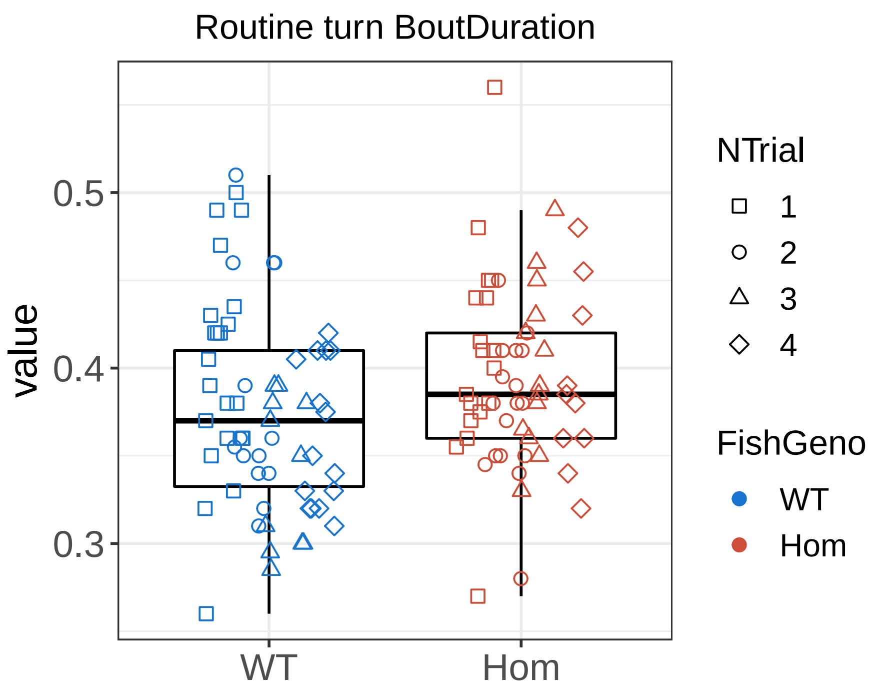


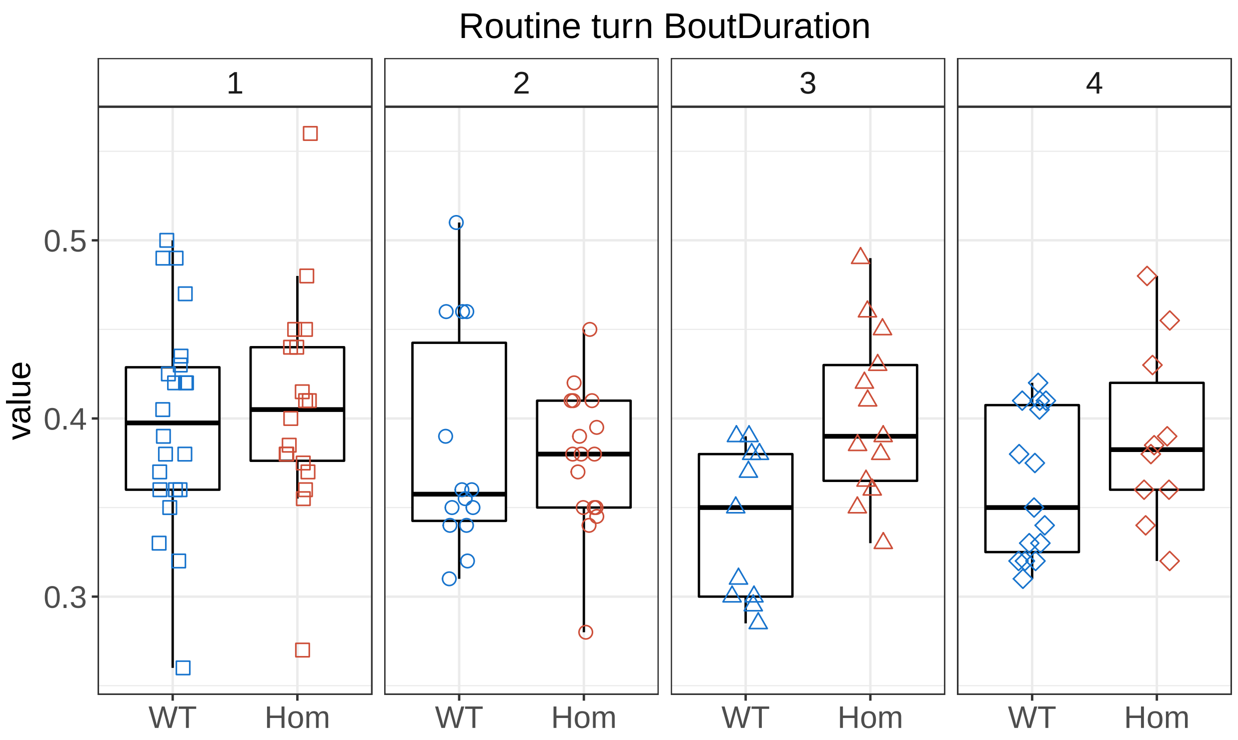

Table 12: Routine turn BoutDuration - Descriptive statistics

| | Min. | 1st Qu. | Median | Mean | 3rd Qu. | Max. |
|---|---|---|---|---|---|---|
| Hom_1 | 0.27 | 0.38 | 0.40 | 0.41 | 0.44 | 0.56 |
| Hom_2 | 0.28 | 0.35 | 0.38 | 0.38 | 0.41 | 0.45 |
| Hom_3 | 0.33 | 0.36 | 0.39 | 0.40 | 0.43 | 0.49 |
| Hom_4 | 0.32 | 0.36 | 0.38 | 0.39 | 0.42 | 0.48 |
| WT_1 | 0.26 | 0.36 | 0.40 | 0.40 | 0.43 | 0.50 |
| WT_2 | 0.31 | 0.34 | 0.36 | 0.38 | 0.44 | 0.51 |
| WT_3 | 0.28 | 0.30 | 0.35 | 0.34 | 0.38 | 0.39 |
| WT_4 | 0.31 | 0.32 | 0.35 | 0.36 | 0.41 | 0.42 |

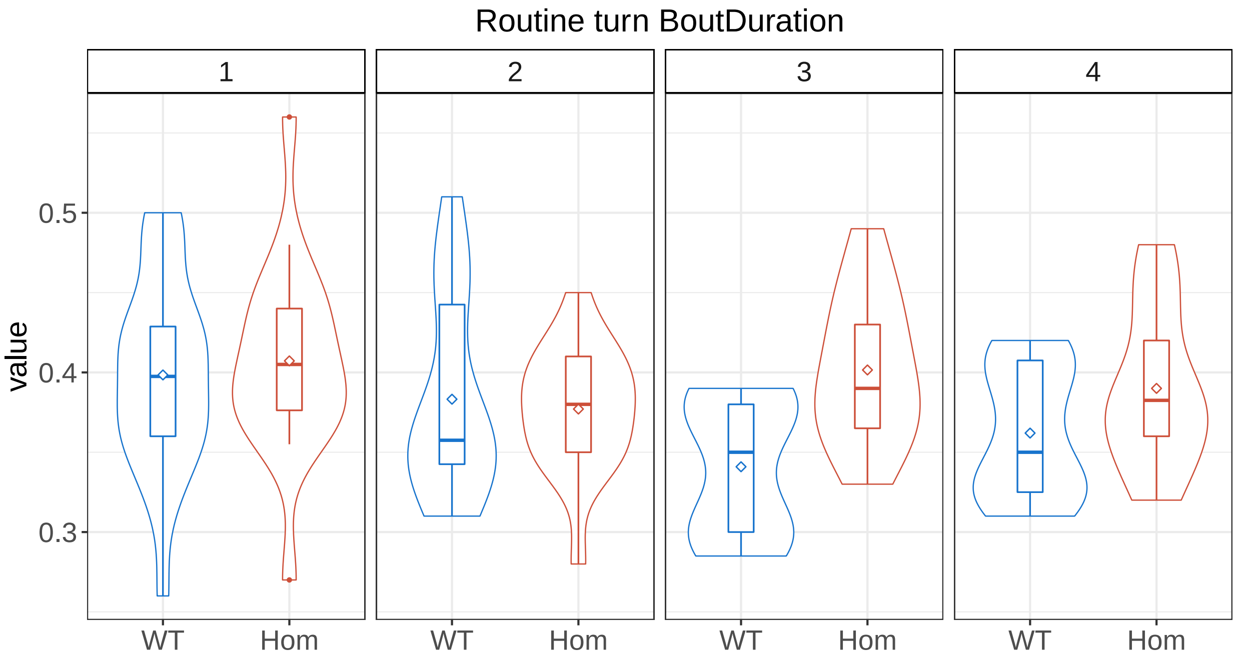

### 4.4.2 Fitting LMM

```
## Linear mixed model fit by REML ['lmerMod']
## Formula: sqrt(value) ~ FishGeno + (1 | NTrial)
##    Data: tmp
## REML criterion at convergence: -400.1581
## Random effects:
##  Groups   Name        Std.Dev.
##  NTrial   (Intercept) 0.00893
##  Residual             0.04244
## Number of obs: 120, groups:  NTrial, 4
## Fixed Effects:
## (Intercept)  FishGenoHom
##     0.61003      0.01561
```

```
## Analysis of Deviance Table (Type II Wald chisquare tests)
##
## Response: sqrt(value)
##          Chisq Df Pr(>Chisq)
## FishGeno 4.025  1    0.04483 *
## ---
## Signif. codes:  0 '***' 0.001 '**' 0.01 '*' 0.05 '.' 0.1 ' ' 1
```

Estimated marginal means with model-based standard errors and 95% confidence limits on the original scale (non-transformed data):

```
## 'emmGrid' object with variables:
##     FishGeno = WT, Hom
## Transformation: "sqrt"
```

```
##  FishGeno response      SE   df lower.CL upper.CL
##  WT          0.372 0.00864 5.72    0.351    0.394
##  Hom         0.391 0.00901 6.18    0.370    0.414
##
## Degrees-of-freedom method: kenward-roger
## Confidence level used: 0.95
## Intervals are back-transformed from the sqrt scale
```

### 4.4.3 Checking model assumptions

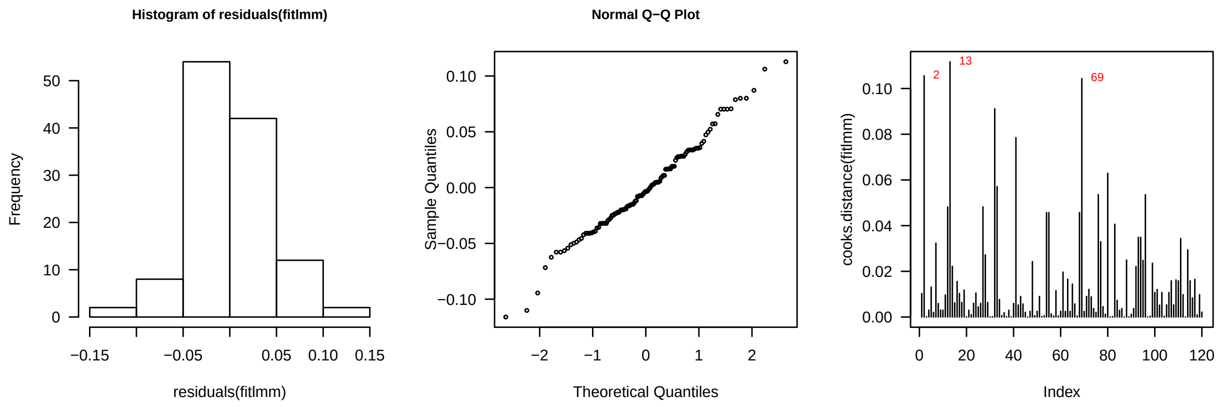


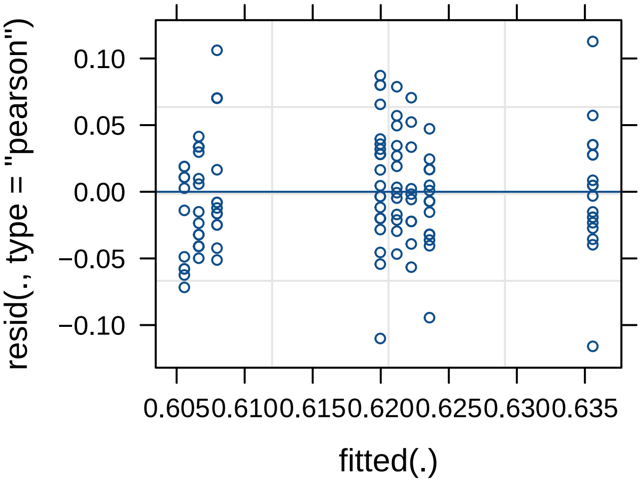


### 4.4.4 Conclusion

Linear mixed model indicated a significant difference between the two genotypes (Type II Wald chi-square test; $^{*}p = 0.045$). Adj. Pvalue: $^{ns}p = 0.203$ (using Meff = 4.518 tests).

## 4.5 Routine turn MedianBendAmp [log, ns]

### 4.5.1 Data visualization with clutch info

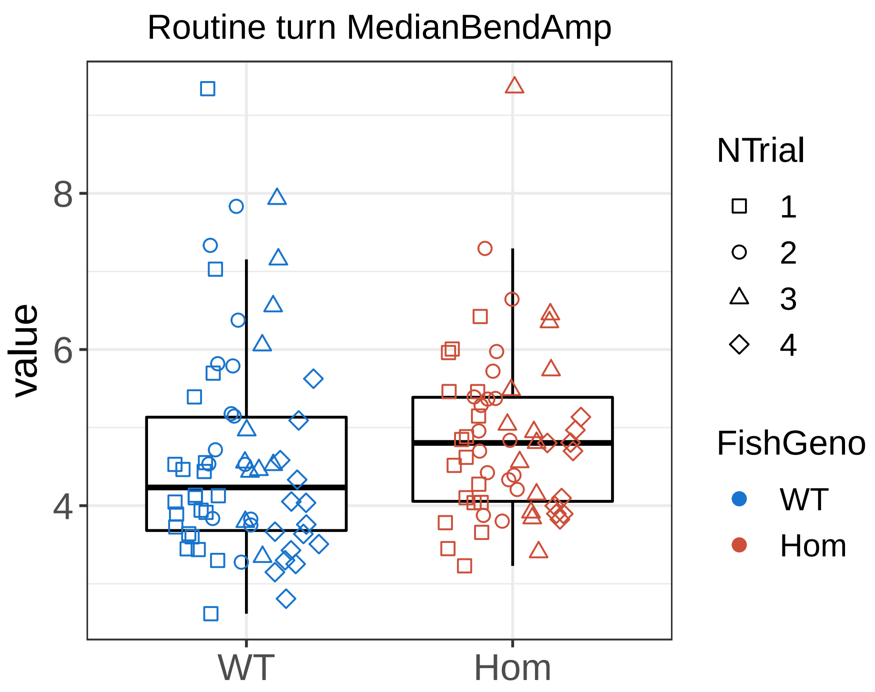


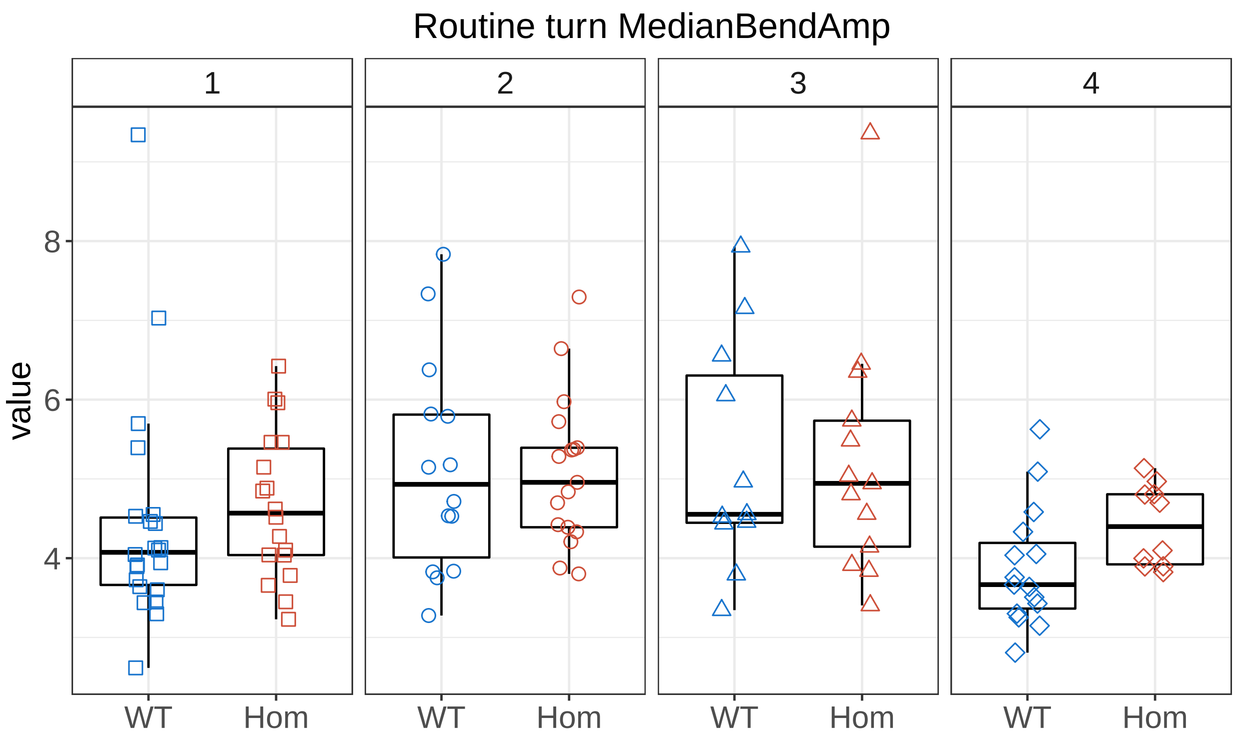

Table 13: Routine turn MedianBendAmp - Descriptive statistics

| | Min. | 1st Qu. | Median | Mean | 3rd Qu. | Max. |
|---|---|---|---|---|---|---|
| Hom_1 | 3.23 | 4.04 | 4.57 | 4.66 | 5.38 | 6.42 |
| Hom_2 | 3.80 | 4.39 | 4.96 | 5.09 | 5.39 | 7.29 |
| Hom_3 | 3.40 | 4.14 | 4.94 | 5.23 | 5.73 | 9.36 |
| Hom_4 | 3.82 | 3.92 | 4.40 | 4.41 | 4.81 | 5.14 |
| WT_1 | 2.62 | 3.66 | 4.07 | 4.43 | 4.51 | 9.34 |
| WT_2 | 3.28 | 4.01 | 4.93 | 5.14 | 5.81 | 7.83 |
| WT_3 | 3.34 | 4.45 | 4.55 | 5.25 | 6.30 | 7.93 |
| WT_4 | 2.81 | 3.36 | 3.67 | 3.88 | 4.19 | 5.63 |

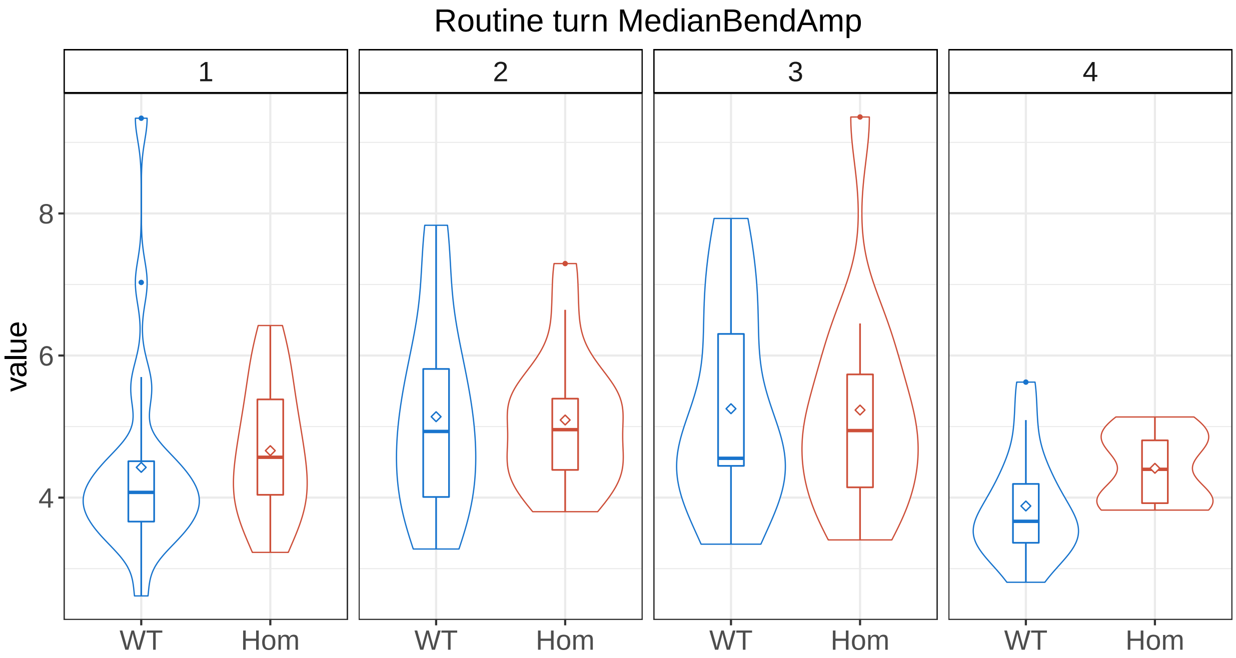

### 4.5.2 Fitting LMM

```
## Linear mixed model fit by REML ['lmerMod']
## Formula: log(value) ~ FishGeno + (1 | NTrial)
##    Data: tmp
## REML criterion at convergence: -2.3412
## Random effects:
##  Groups   Name        Std.Dev.
##  NTrial   (Intercept) 0.09369
##  Residual             0.22621
## Number of obs: 120, groups:  NTrial, 4
## Fixed Effects:
## (Intercept)  FishGenoHom
##     1.49909      0.05658
```

```
## Analysis of Deviance Table (Type II Wald chisquare tests)
##
## Response: log(value)
##           Chisq Df Pr(>Chisq)
## FishGeno 1.8526  1     0.1735
```

Estimated marginal means with model-based standard errors and 95% confidence limits on the original scale (non-transformed data):

```
## 'emmGrid' object with variables:
##     FishGeno = WT, Hom
## Transformation: "log"
```

```
##  FishGeno response    SE   df lower.CL upper.CL
##  WT           4.48 0.247 3.97     3.84     5.22
##  Hom          4.74 0.264 4.11     4.07     5.52
##
## Degrees-of-freedom method: kenward-roger
## Confidence level used: 0.95
## Intervals are back-transformed from the log scale
```

### 4.5.3 Checking model assumptions

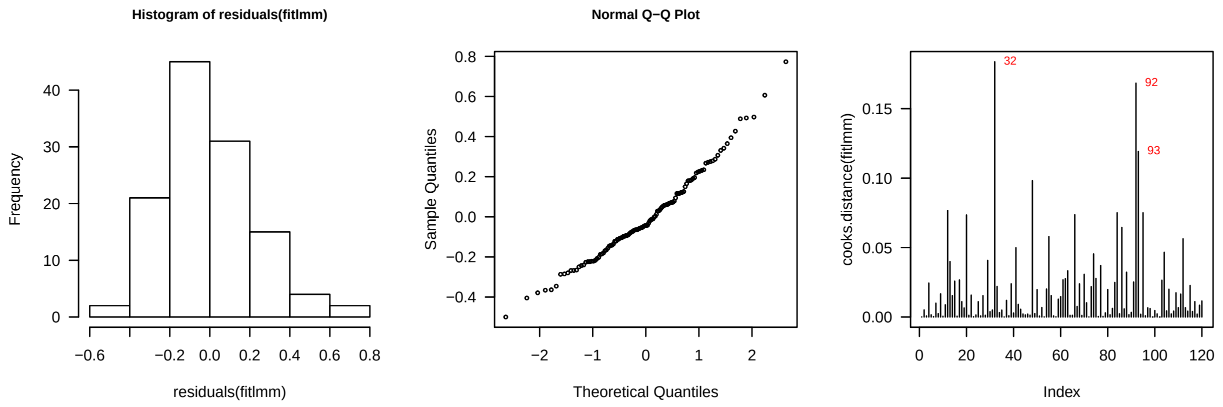


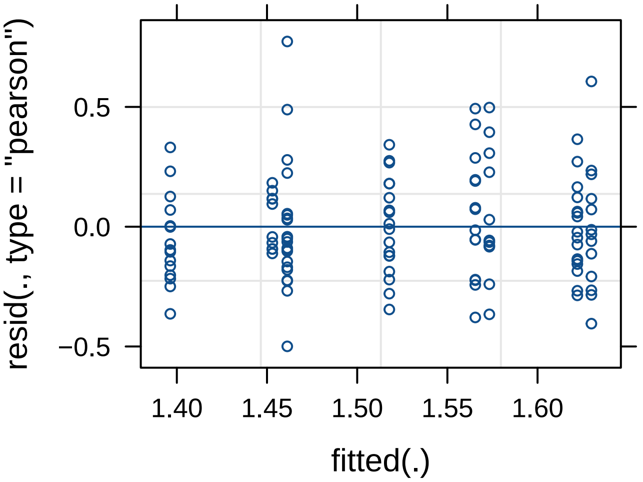


### 4.5.4 Conclusion

No significant difference was detected between the two groups (Type II Wald chi-square test; $^{ns}$p = 0.174). Adj. Pvalue: $^{ns}$p = 0.784 (using Meff = 4.518 tests).

### 4.6 Routine turn NumOfOsc [ns]

#### 4.6.1 Data visualization with clutch info

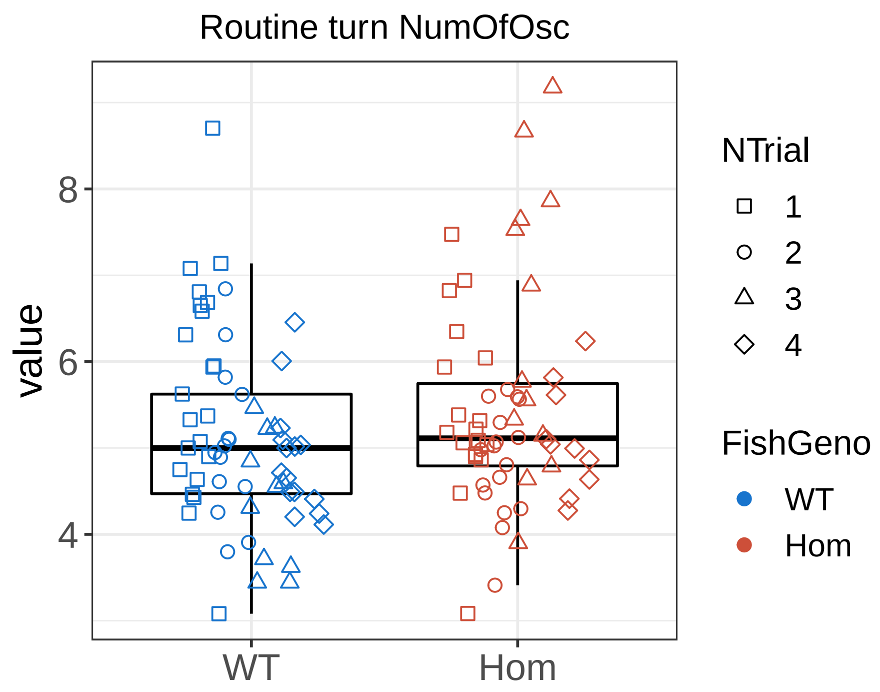


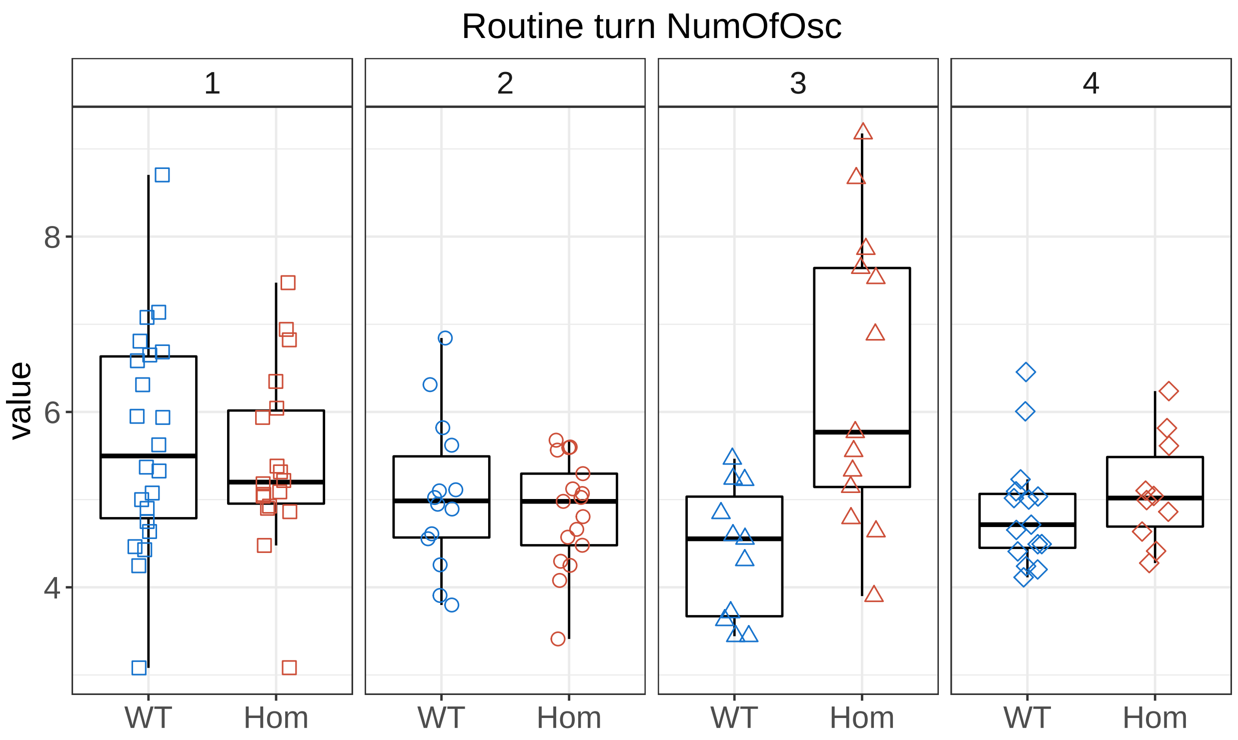

Table 14: Routine turn NumOfOsc - Descriptive statistics

| | Min. | 1st Qu. | Median | Mean | 3rd Qu. | Max. |
|---|---|---|---|---|---|---|
| Hom_1 | 3.08 | 4.95 | 5.20 | 5.45 | 6.02 | 7.47 |
| Hom_2 | 3.41 | 4.48 | 4.98 | 4.85 | 5.30 | 5.68 |
| Hom_3 | 3.90 | 5.14 | 5.77 | 6.37 | 7.64 | 9.18 |
| Hom_4 | 4.28 | 4.69 | 5.02 | 5.10 | 5.49 | 6.24 |
| WT_1 | 3.08 | 4.79 | 5.50 | 5.67 | 6.63 | 8.70 |
| WT_2 | 3.80 | 4.57 | 4.99 | 5.06 | 5.49 | 6.84 |
| WT_3 | 3.44 | 3.67 | 4.55 | 4.40 | 5.03 | 5.47 |
| WT_4 | 4.11 | 4.45 | 4.71 | 4.88 | 5.07 | 6.46 |

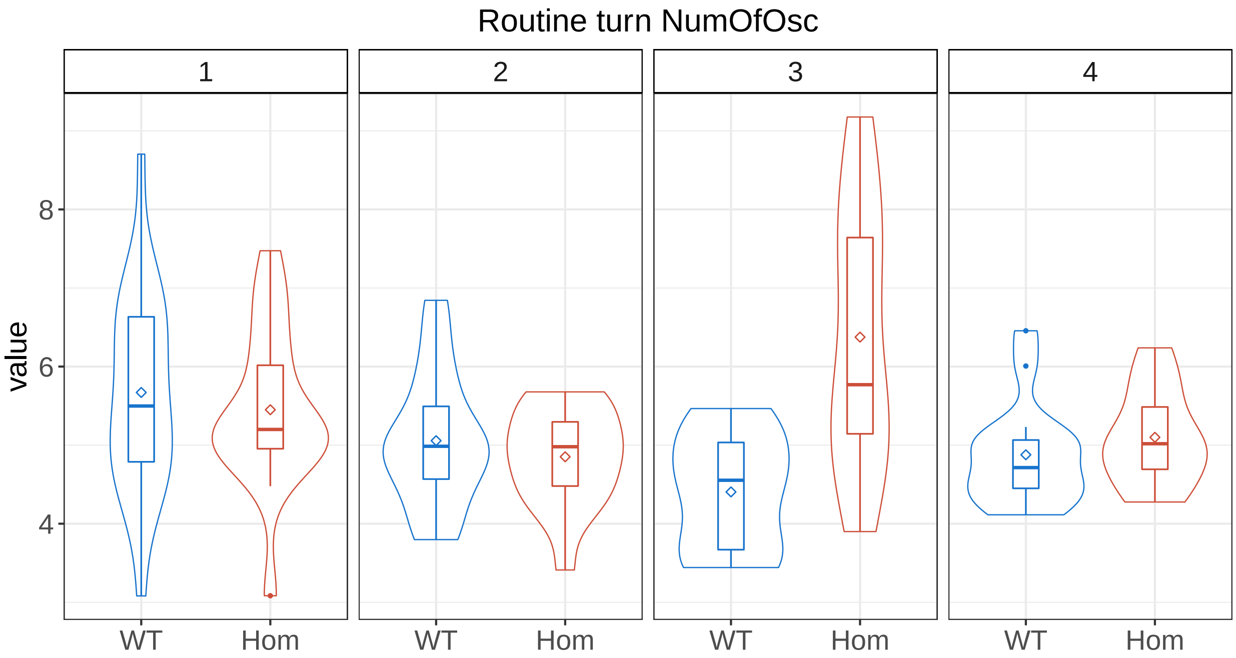

### 4.6.2 Fitting LMM

```
## Linear mixed model fit by REML ['lmerMod']
## Formula: value ~ FishGeno + (1 | NTrial)
##    Data: tmp
## REML criterion at convergence: 366.8914
## Random effects:
##  Groups   Name        Std.Dev.
##  NTrial   (Intercept) 0.2704
##  Residual             1.0919
## Number of obs: 120, groups:  NTrial, 4
## Fixed Effects:
## (Intercept)  FishGenoHom
##      5.0930       0.3145
```

```
## Analysis of Deviance Table (Type II Wald chisquare tests)
##
## Response: value
##           Chisq Df Pr(>Chisq)
## FishGeno 2.4639  1     0.1165
```

Estimated marginal means with model-based standard errors and 95% confidence limits on the original scale (non-transformed data):

```
## 'emmGrid' object with variables:
##     FishGeno = WT, Hom
```

```
##  FishGeno emmean    SE   df lower.CL upper.CL
##  WT         5.09 0.196 5.20     4.60     5.59
##  Hom        5.41 0.199 5.56     4.91     5.90
##
## Degrees-of-freedom method: kenward-roger
## Confidence level used: 0.95
```

### 4.6.3 Checking model assumptions

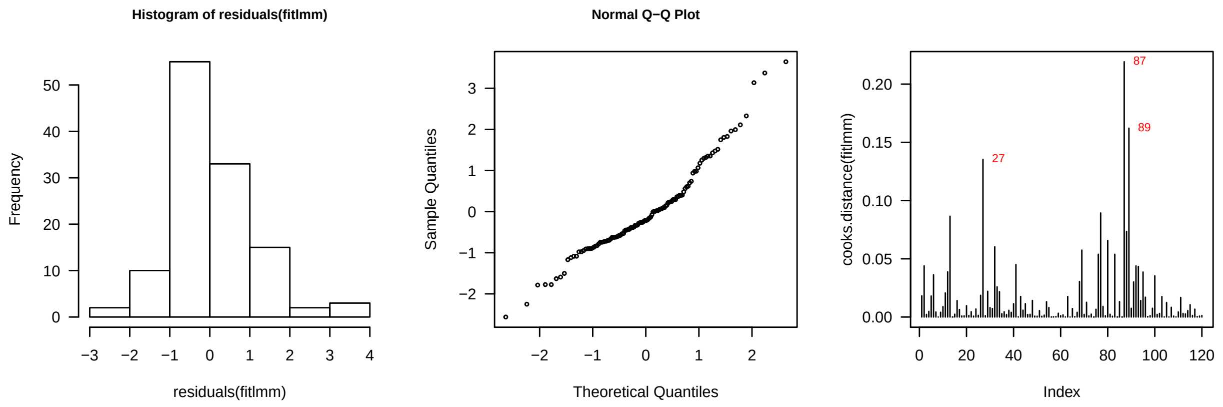


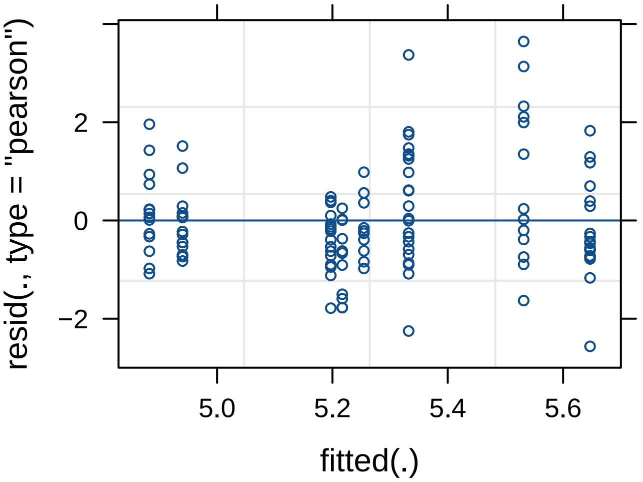


### 4.6.4 Conclusion

No significant difference was detected between the two groups (Type II Wald chi-square test; $^{ns}$p = 0.117). Adj. Pvalue: $^{ns}$p = 0.526 (using Meff = 4.518 tests).

## 4.7 Routine turn Speed [ns]

### 4.7.1 Data visualization with clutch info

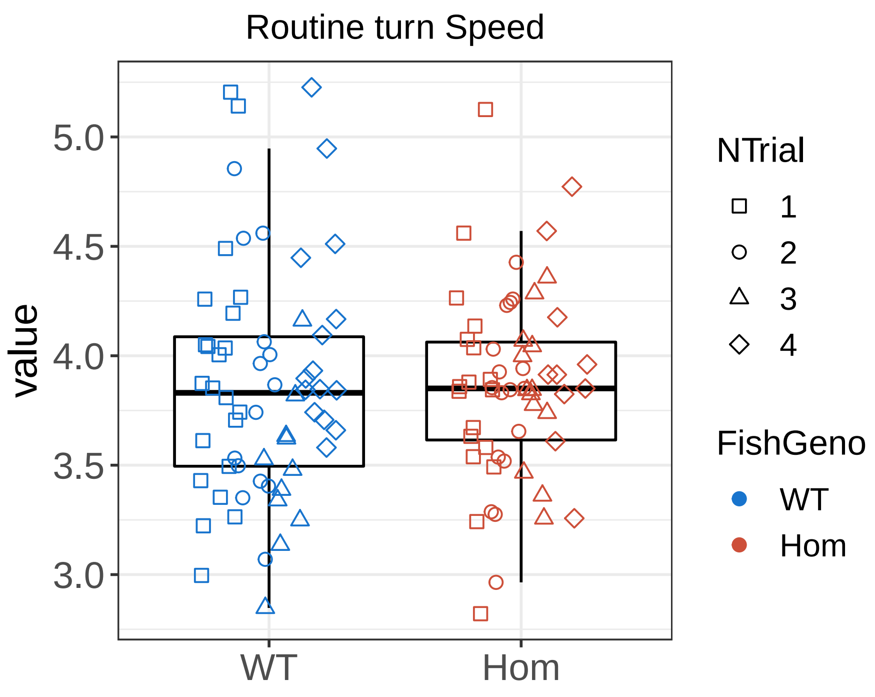


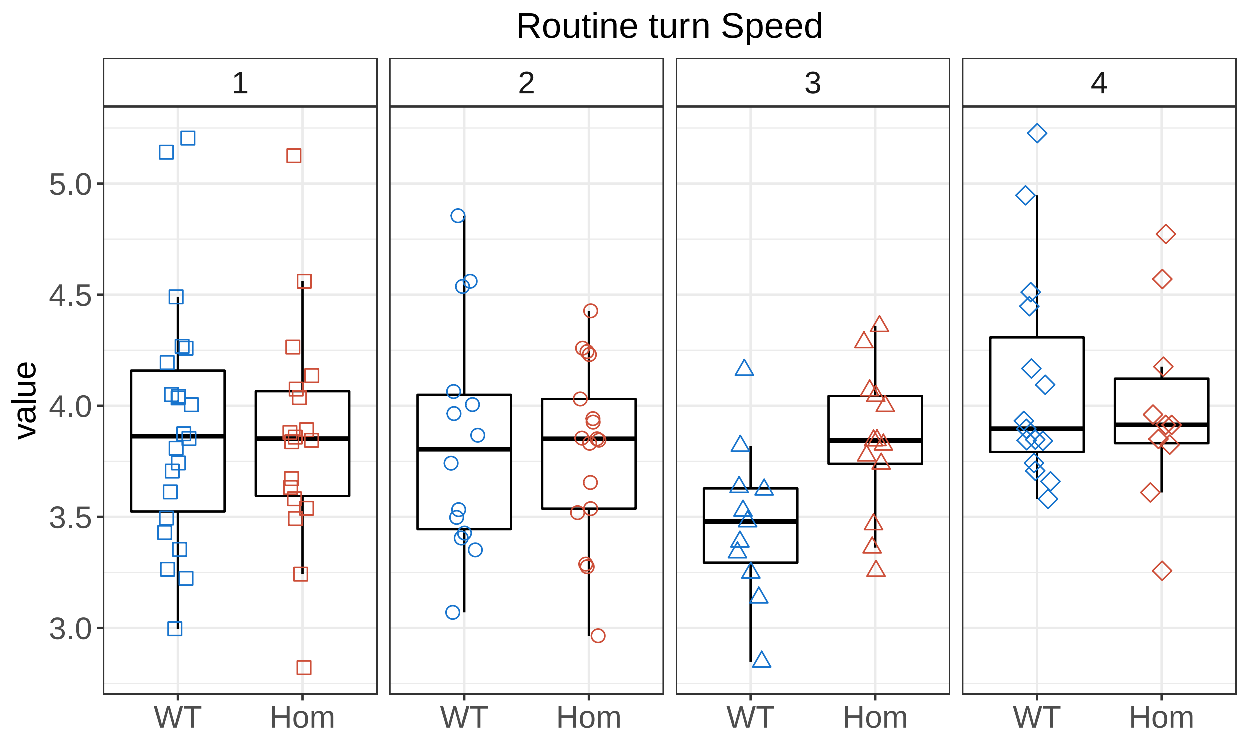

Table 15: Routine turn Speed - Descriptive statistics

| | Min. | 1st Qu. | Median | Mean | 3rd Qu. | Max. |
|---|---|---|---|---|---|---|
| Hom_1 | 2.82 | 3.59 | 3.85 | 3.86 | 4.07 | 5.13 |
| Hom_2 | 2.96 | 3.54 | 3.85 | 3.80 | 4.03 | 4.43 |
| Hom_3 | 3.26 | 3.74 | 3.84 | 3.84 | 4.04 | 4.36 |
| Hom_4 | 3.26 | 3.83 | 3.91 | 3.98 | 4.12 | 4.77 |
| WT_1 | 3.00 | 3.52 | 3.86 | 3.91 | 4.16 | 5.20 |
| WT_2 | 3.07 | 3.44 | 3.80 | 3.85 | 4.05 | 4.86 |
| WT_3 | 2.85 | 3.29 | 3.48 | 3.47 | 3.63 | 4.16 |
| WT_4 | 3.58 | 3.79 | 3.90 | 4.10 | 4.31 | 5.23 |

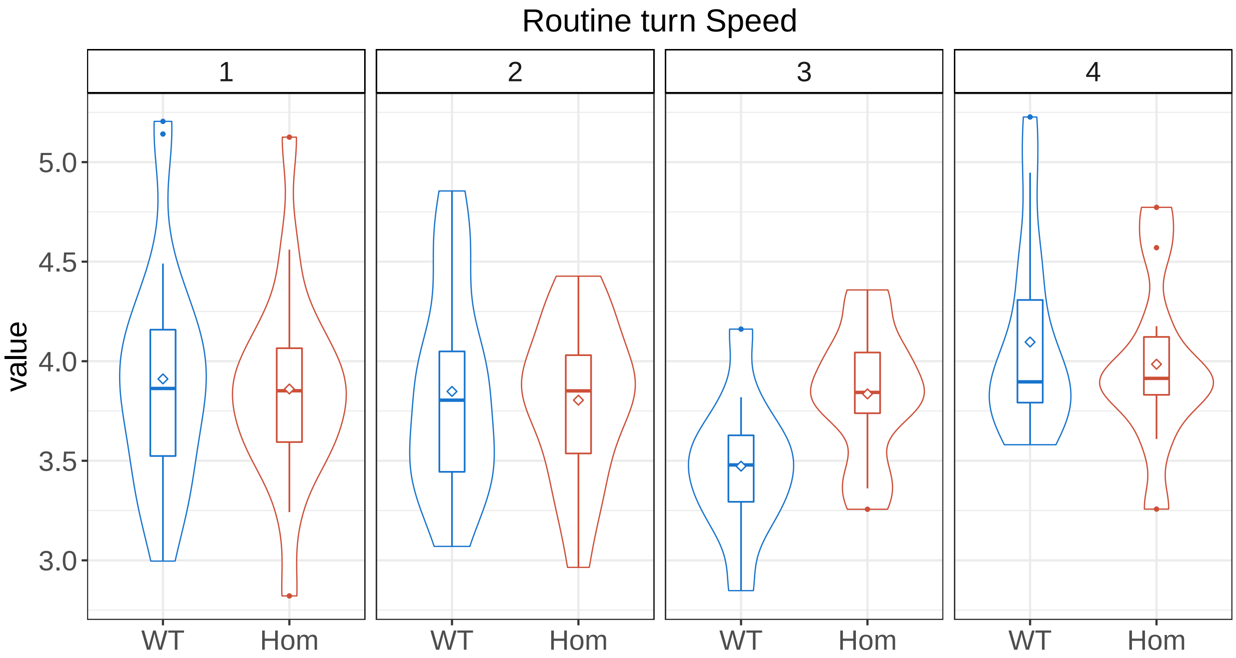

### 4.7.2 Fitting LMM

```
## Linear mixed model fit by REML ['lmerMod']
## Formula: value ~ FishGeno + (1 | NTrial)
##    Data: tmp
## REML criterion at convergence: 167.7369
## Random effects:
##  Groups   Name        Std.Dev.
##  NTrial   (Intercept) 0.1227
##  Residual             0.4692
## Number of obs: 120, groups:  NTrial, 4
## Fixed Effects:
## (Intercept)  FishGenoHom
##     3.85358      0.01258
```

```
## Analysis of Deviance Table (Type II Wald chisquare tests)
##
## Response: value
##           Chisq Df Pr(>Chisq)
## FishGeno 0.0213  1     0.8839
```

Estimated marginal means with model-based standard errors and 95% confidence limits on the original scale (non-transformed data):

```
## 'emmGrid' object with variables:
##     FishGeno = WT, Hom
```

```
##  FishGeno emmean     SE   df lower.CL upper.CL
##  WT         3.85 0.0863 5.04     3.63     4.07
##  Hom        3.87 0.0875 5.36     3.65     4.09
##
## Degrees-of-freedom method: kenward-roger
## Confidence level used: 0.95
```

### 4.7.3 Checking model assumptions

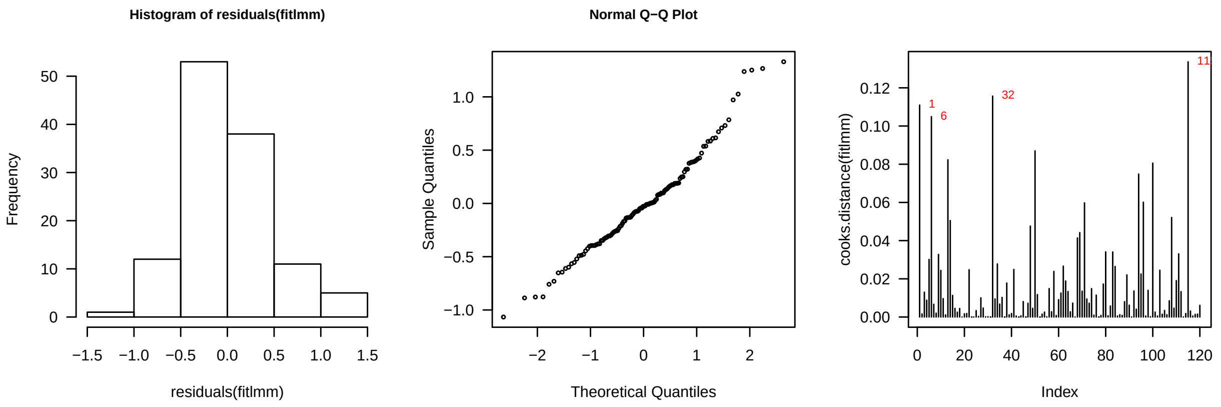


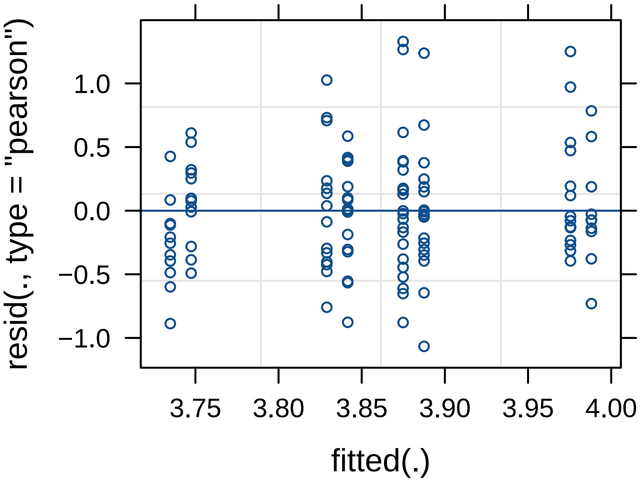


### 4.7.4 Conclusion

No significant difference was detected between the two groups (Type II Wald chi-square test; $^{ns}$p = 0.884). Adj. Pvalue: $^{ns}$p = 1 (using Meff = 4.518 tests).

## 4.8 Routine turn TBF [ns]

### 4.8.1 Data visualization with clutch info

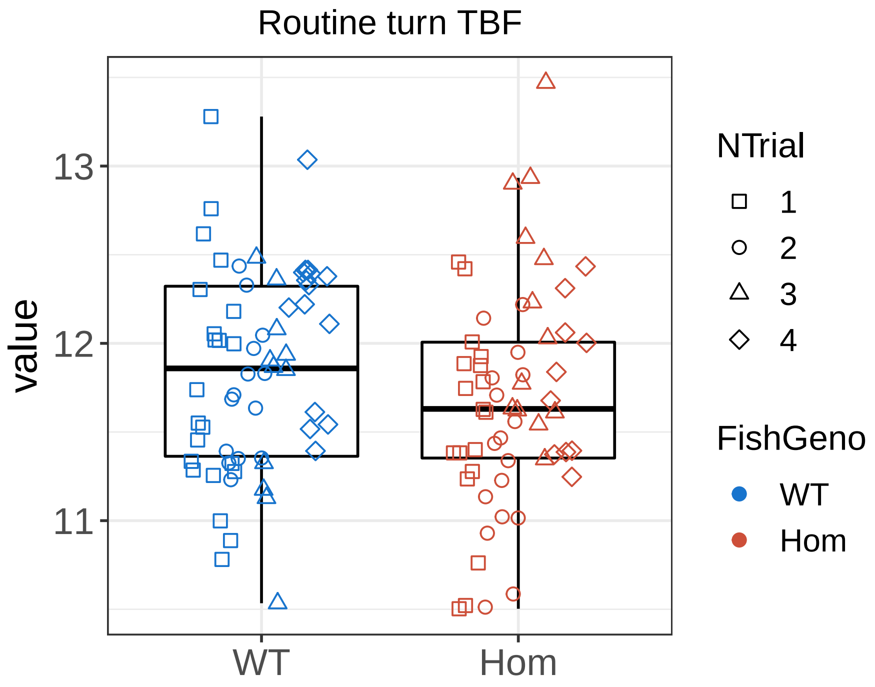


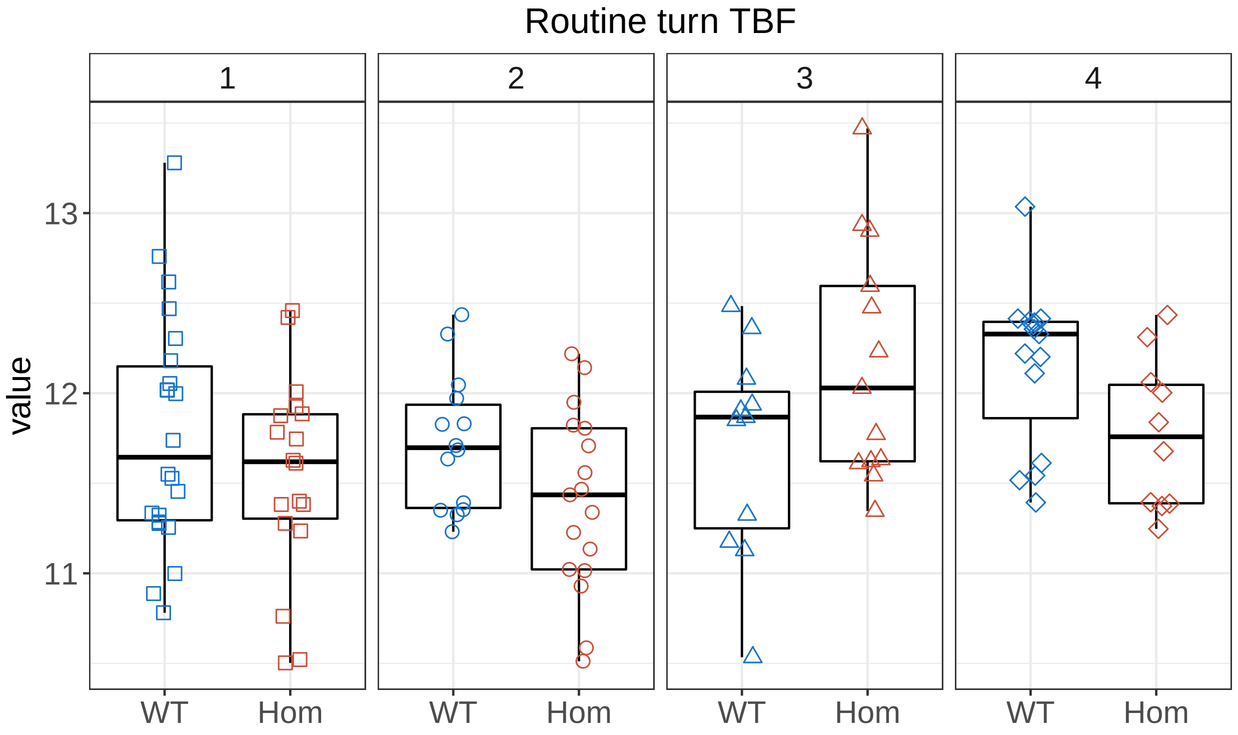

Table 16: Routine turn TBF - Descriptive statistics

| | Min. | 1st Qu. | Median | Mean | 3rd Qu. | Max. |
|---|---|---|---|---|---|---|
| Hom_1 | 10.50 | 11.30 | 11.62 | 11.54 | 11.88 | 12.46 |
| Hom_2 | 10.51 | 11.02 | 11.44 | 11.40 | 11.81 | 12.22 |
| Hom_3 | 11.35 | 11.62 | 12.03 | 12.17 | 12.60 | 13.47 |
| Hom_4 | 11.25 | 11.39 | 11.76 | 11.77 | 12.05 | 12.43 |
| WT_1 | 10.78 | 11.29 | 11.64 | 11.78 | 12.15 | 13.28 |
| WT_2 | 11.23 | 11.36 | 11.70 | 11.72 | 11.94 | 12.44 |
| WT_3 | 10.53 | 11.25 | 11.87 | 11.69 | 12.01 | 12.48 |
| WT_4 | 11.39 | 11.86 | 12.33 | 12.15 | 12.40 | 13.04 |

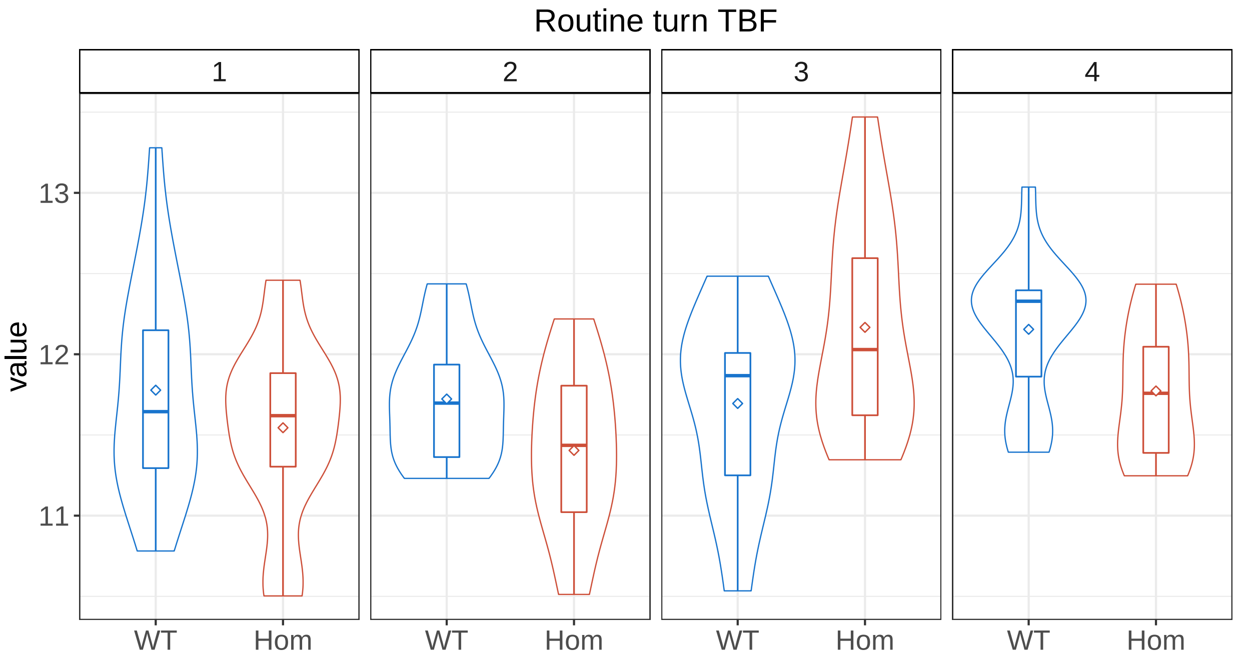

### 4.8.2 Fitting LMM

```
## Linear mixed model fit by REML ['lmerMod']
## Formula: value ~ FishGeno + (1 | NTrial)
##    Data: tmp
## REML criterion at convergence: 210.5648
## Random effects:
##  Groups   Name        Std.Dev.
##  NTrial   (Intercept) 0.1853
##  Residual             0.5601
## Number of obs: 120, groups:  NTrial, 4
## Fixed Effects:
## (Intercept)  FishGenoHom
##     11.8579      -0.1475
```

```
## Analysis of Deviance Table (Type II Wald chisquare tests)
##
## Response: value
##           Chisq Df Pr(>Chisq)
## FishGeno 2.0543  1     0.1518
```

Estimated marginal means with model-based standard errors and 95% confidence limits on the original scale (non-transformed data):

```
## 'emmGrid' object with variables:
##     FishGeno = WT, Hom
```

```
##  FishGeno emmean    SE   df lower.CL upper.CL
##  WT         11.9 0.118 4.42     11.5     12.2
##  Hom        11.7 0.119 4.64     11.4     12.0
##
## Degrees-of-freedom method: kenward-roger
## Confidence level used: 0.95
```

### 4.8.3 Checking model assumptions

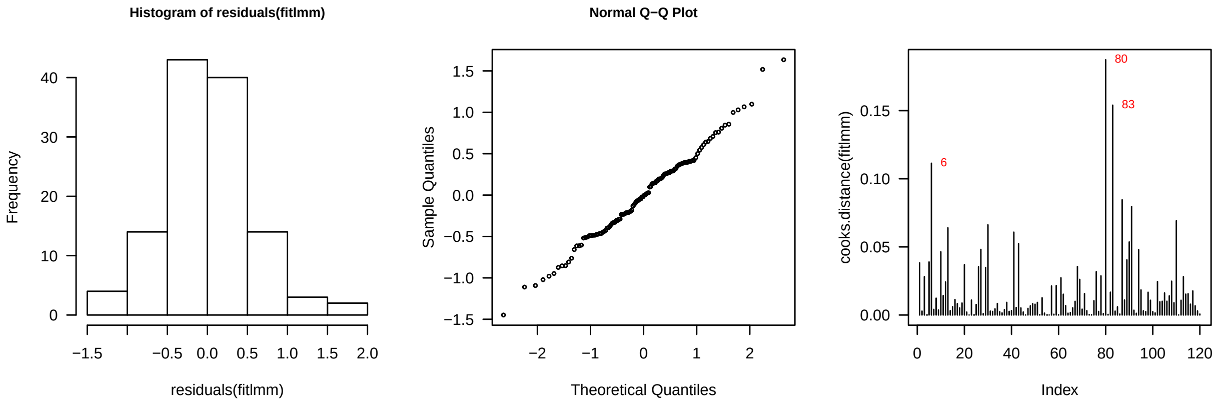


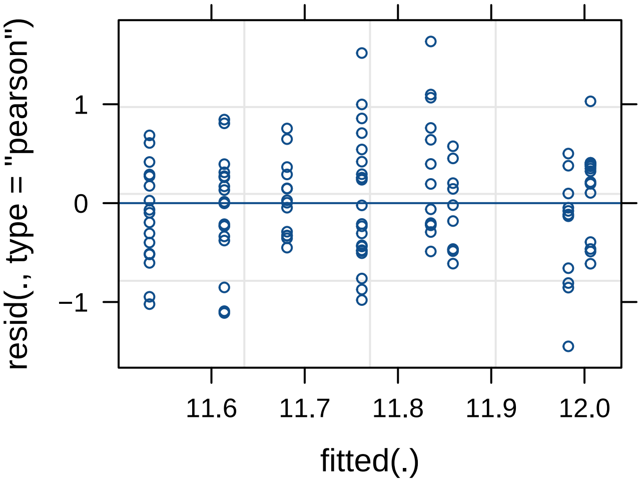


### 4.8.4 Conclusion

No significant difference was detected between the two groups (Type II Wald chi-square test; $^{ns}$p = 0.152). Adj. Pvalue: $^{ns}$p = 0.686 (using Meff = 4.518 tests).

# 5 All bouts

## 5.1 Descriptive statistics

Table 17: ALL WT - Mean values by clutch

| | WT_1 | WT_2 | WT_3 | WT_4 | MEAN |
|---|---|---|---|---|---|
| BoutRate | 0.25 | 0.23 | 0.19 | 0.24 | 0.23 |
| BoutFrequency | 1.63 | 1.45 | 1.52 | 1.60 | 1.55 |

Table 18: ALL Hom - Mean values by clutch

| | Hom_1 | Hom_2 | Hom_3 | Hom_4 | MEAN |
|---|---|---|---|---|---|
| BoutRate | 0.26 | 0.23 | 0.15 | 0.25 | 0.22 |
| BoutFrequency | 1.39 | 1.46 | 1.82 | 1.74 | 1.60 |

# 6 All bouts

## 6.1 All bouts BoutRate [ns]

### 6.1.1 Data visualization with clutch info

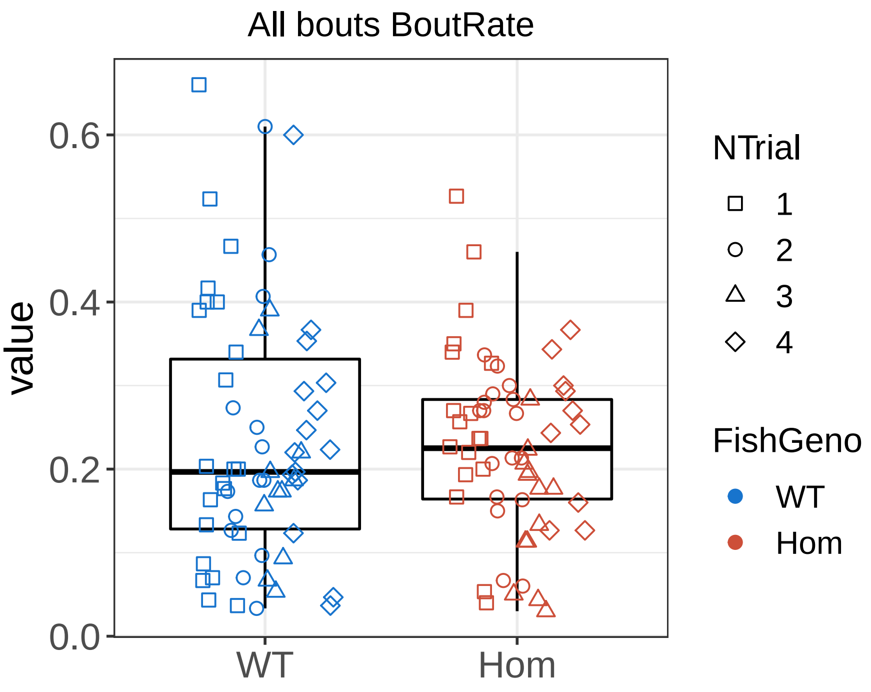


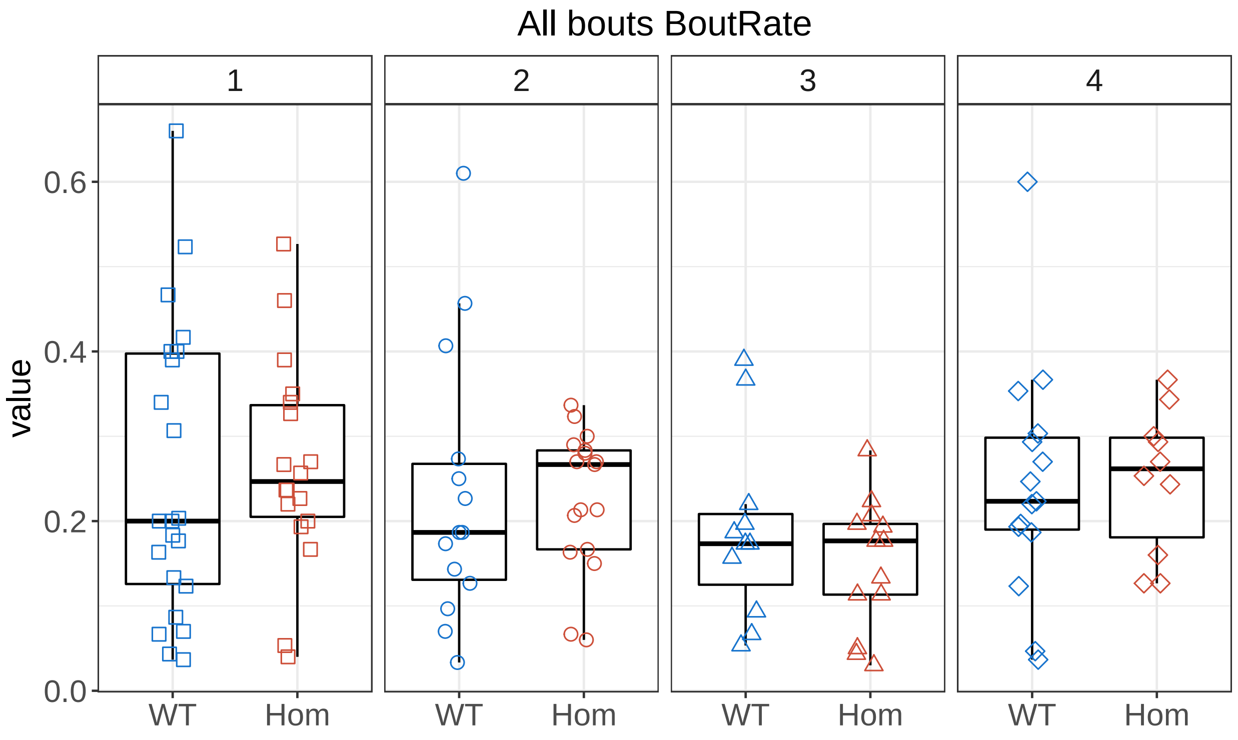

Table 19: All bouts BoutRate - Descriptive statistics

| | Min. | 1st Qu. | Median | Mean | 3rd Qu. | Max. |
|---|---|---|---|---|---|---|
| Hom_1 | 0.04 | 0.20 | 0.25 | 0.26 | 0.34 | 0.53 |
| Hom_2 | 0.06 | 0.17 | 0.27 | 0.23 | 0.28 | 0.34 |
| Hom_3 | 0.03 | 0.11 | 0.18 | 0.15 | 0.20 | 0.28 |
| Hom_4 | 0.13 | 0.18 | 0.26 | 0.25 | 0.30 | 0.37 |
| WT_1 | 0.04 | 0.13 | 0.20 | 0.25 | 0.40 | 0.66 |
| WT_2 | 0.03 | 0.13 | 0.19 | 0.23 | 0.27 | 0.61 |
| WT_3 | 0.05 | 0.13 | 0.17 | 0.19 | 0.21 | 0.39 |
| WT_4 | 0.04 | 0.19 | 0.22 | 0.24 | 0.30 | 0.60 |

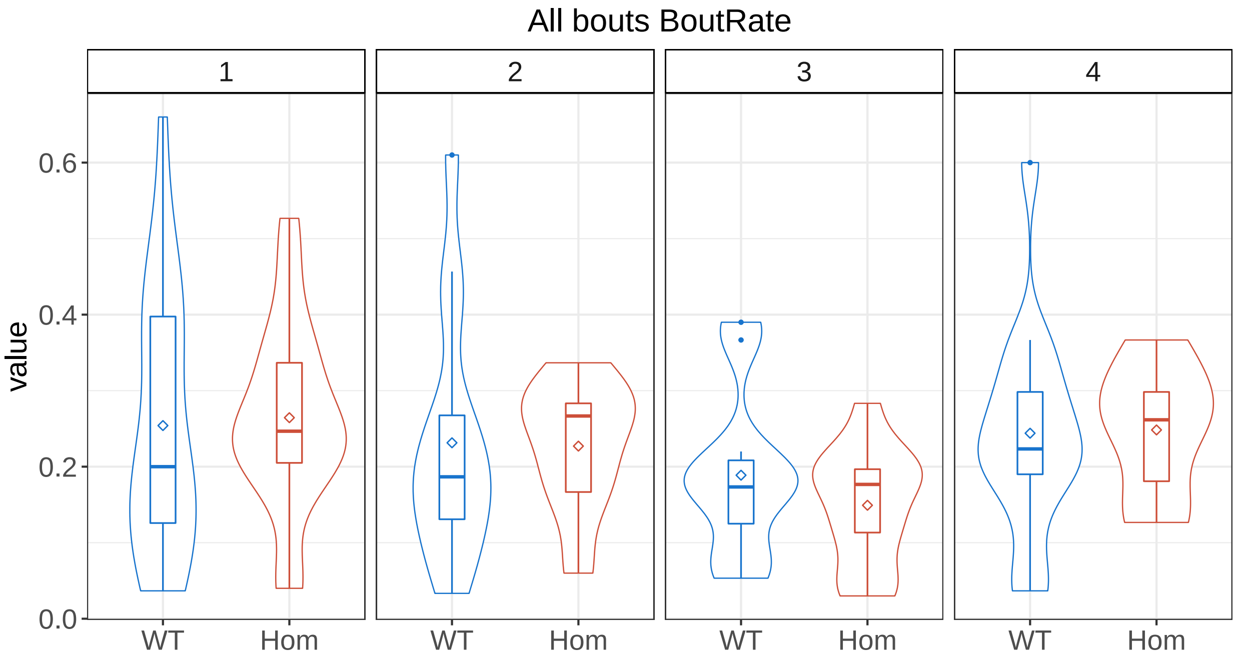

### 6.1.2 Fitting LMM

```
## Linear mixed model fit by REML ['lmerMod']
## Formula: value ~ FishGeno + (1 | NTrial)
##    Data: tmp
## REML criterion at convergence: -140.379
## Random effects:
##  Groups   Name        Std.Dev.
##  NTrial   (Intercept) 0.03103
##  Residual             0.12730
## Number of obs: 120, groups:  NTrial, 4
## Fixed Effects:
## (Intercept)  FishGenoHom
##      0.2302      -0.0068
```

```
## Analysis of Deviance Table (Type II Wald chisquare tests)
##
## Response: value
##           Chisq Df Pr(>Chisq)
## FishGeno 0.0848  1      0.771
```

Estimated marginal means with model-based standard errors and 95% confidence limits on the original scale (non-transformed data):

```
## 'emmGrid' object with variables:
##     FishGeno = WT, Hom
```

```
##  FishGeno emmean     SE   df lower.CL upper.CL
##  WT        0.230 0.0226 5.25    0.173    0.288
##  Hom       0.223 0.0230 5.61    0.166    0.281
##
## Degrees-of-freedom method: kenward-roger
## Confidence level used: 0.95
```

### 6.1.3 Checking model assumptions

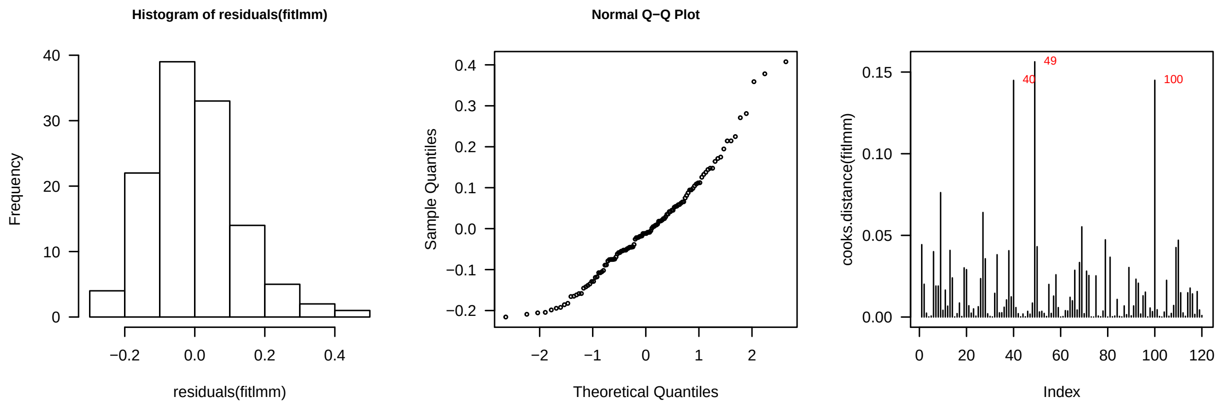


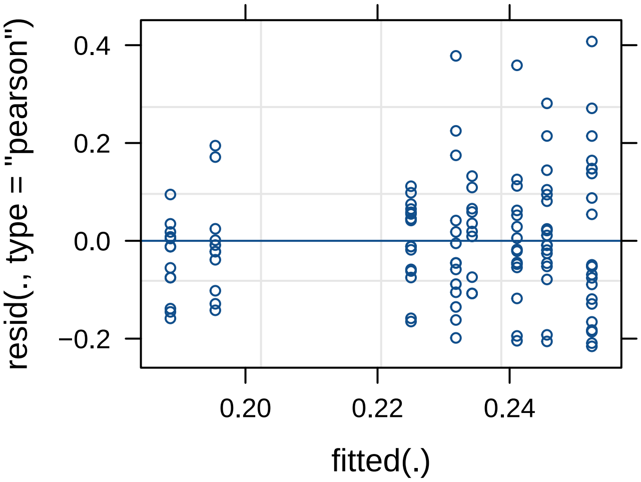


### 6.1.4 Conclusion

No significant difference was detected between the two genotypes (Type II Wald chi-square test; $^{ns}$p = 0.771).

## 6.2 All bouts Distribution of MaxBendAmp

### 6.2.1 Histogram of MaxBendAmp

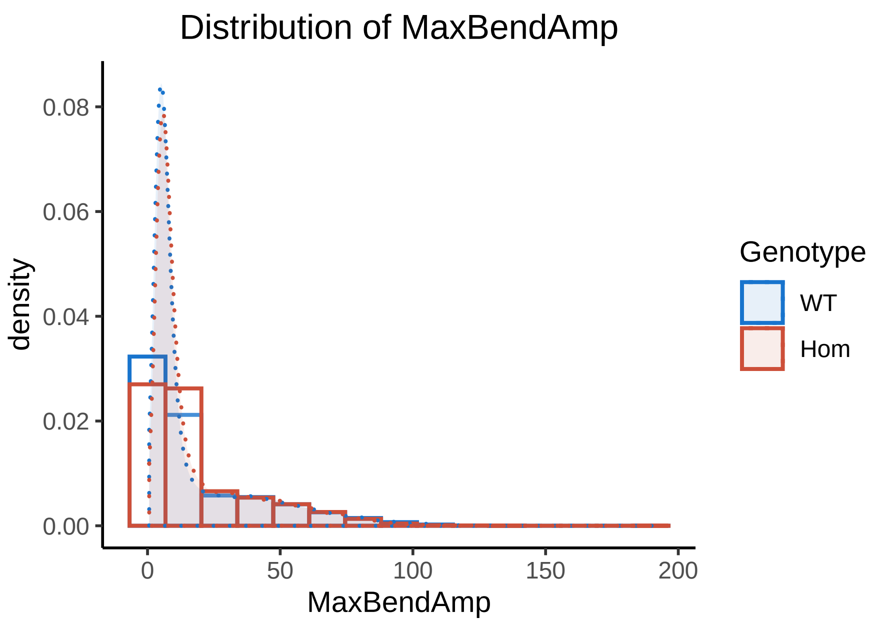


### 6.2.2 Histogram of log(MaxBendAmp)

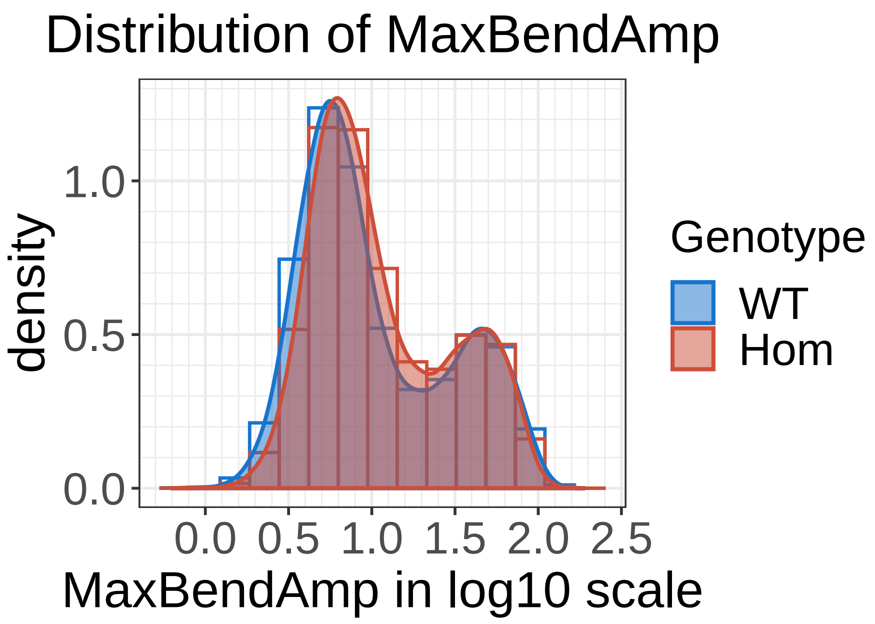

# 7 R session information

```
## R version 3.5.2 (2018-12-20)
## Platform: x86_64-pc-linux-gnu (64-bit)
## Running under: Linux Mint 19.1
##
## Matrix products: default
## BLAS: /opt/microsoft/ropen/3.5.2/lib64/R/lib/libRblas.so
## LAPACK: /opt/microsoft/ropen/3.5.2/lib64/R/lib/libRlapack.so
##
## locale:
##  [1] LC_CTYPE=en_GB.UTF-8       LC_NUMERIC=C
##  [3] LC_TIME=en_GB.UTF-8        LC_COLLATE=en_GB.UTF-8
##  [5] LC_MONETARY=fr_FR.UTF-8    LC_MESSAGES=en_GB.UTF-8
##  [7] LC_PAPER=fr_FR.UTF-8       LC_NAME=C
##  [9] LC_ADDRESS=C               LC_TELEPHONE=C
## [11] LC_MEASUREMENT=fr_FR.UTF-8 LC_IDENTIFICATION=C
##
## attached base packages:
## [1] stats     graphics  grDevices utils     datasets  methods   base
##
## other attached packages:
##  [1] corrplot_0.84       emmeans_1.3.2       readxl_1.2.0
##  [4] reshape2_1.4.3      lme4_1.1-19         Matrix_1.2-15
##  [7] car_3.0-2           carData_3.0-2       data.table_1.12.2
## [10] knitr_1.22          RColorBrewer_1.1-2  gridExtra_2.3
## [13] ggplot2_3.2.1       RevoUtils_11.0.2    RevoUtilsMath_11.0.0
##
## loaded via a namespace (and not attached):
##  [1] Rcpp_1.0.3       mvtnorm_1.0-8     lattice_0.20-38  zoo_1.8-7
##  [5] assertthat_0.2.1 digest_0.6.18     R6_2.3.0         cellranger_1.1.0
##  [9] plyr_1.8.4       evaluate_0.13     coda_0.19-2      highr_0.8
## [13] pillar_1.3.1     rlang_0.4.4       lazyeval_0.2.2   curl_3.3
## [17] multcomp_1.4-8   minqa_1.2.4       nloptr_1.2.1     rmarkdown_1.11
## [21] labeling_0.3     splines_3.5.2     stringr_1.4.0    foreign_0.8-71
## [25] munsell_0.5.0    compiler_3.5.2    xfun_0.6         pkgconfig_2.0.2
## [29] htmltools_0.3.6  tidyselect_0.2.5  tibble_2.1.3     codetools_0.2-15
## [33] rio_0.5.16       crayon_1.3.4      dplyr_0.8.4      withr_2.1.2
## [37] MASS_7.3-51.5    grid_3.5.2        nlme_3.1-137     xtable_1.8-4
## [41] gtable_0.3.0     magrittr_1.5      scales_1.0.0     zip_1.0.0
## [45] estimability_1.3 stringi_1.2.4     sandwich_2.5-0   openxlsx_4.1.0
## [49] TH.data_1.0-10   tools_3.5.2       forcats_0.3.0    glue_1.3.1
## [53] purrr_0.3.2      hms_0.4.2         parallel_3.5.2   pbkrtest_0.4-7
## [57] survival_2.43-3  abind_1.4-5       yaml_2.2.0       colorspace_1.4-1
## [61] haven_2.0.0
```
