# Supplementary material for: Somatostatin 1.1 contributes to the innate exploration of zebrafish larva
Source: Sci Rep. 2020 Sep 17;10:15235. doi: 10.1038/s41598-020-72039-x (PMC7499426; doi:10.1038/s41598-020-72039-x)
Supplement: Supplementary file 3 — Supplementary Information 3. [file 41598_2020_72039_MOESM3_ESM.pdf]

SUPPLEMENTAL INFORMATION

Title

Somatostatin 1.1 contributes to the innate exploration of larval zebrafish

Authors

Feng B Quan[1,2], Laura Desban[1,3], Olivier Mirat[1], Maxime Kermarquer[1], Julian Roussel[1], Fanny Koeth[1], Hugo Marnas[1], Lydia Djenoune[1,4], Francois-Xavier Lejeune[1], Hervé Tostivint[2], Claire Wyart[1,#]

Affiliations

[1]Sorbonne Université, Institut du Cerveau (ICM), F-75013, Paris, France.

[2]Muséum National d'Histoire Naturelle (MNHN), CNRS UMR 7221, Paris, France.

[3]Current address: Institute of Neuroscience, University of Oregon, Eugene, Oregon, USA.

[4]Current address: Cardiovascular Research Center, Massachusetts General Hospital, Charlestown, MA 02129, USA; Harvard Medical School, Boston, MA 02115, USA.

# Corresponding author: Claire Wyart, claire.wyart@icm-institute.org, Institut du Cerveau (ICM), Campus Hospitalier Universitaire Pitié-Salpêtrière, 47, bld de l'Hôpital, 75013 Paris, France.

Supplemental Videos

Video S1. Tracking acousto-vestibular escapes responses of 32 zebrafish larvae. Related to Figure 1.

Acousto-vestibular escapes responses of 32 zebrafish larvae were tracked using the ZebraZoom algorithm, which detects the head direction (white line), the body position (red dots) and the tail end position (blue dot).

Supplemental Figures

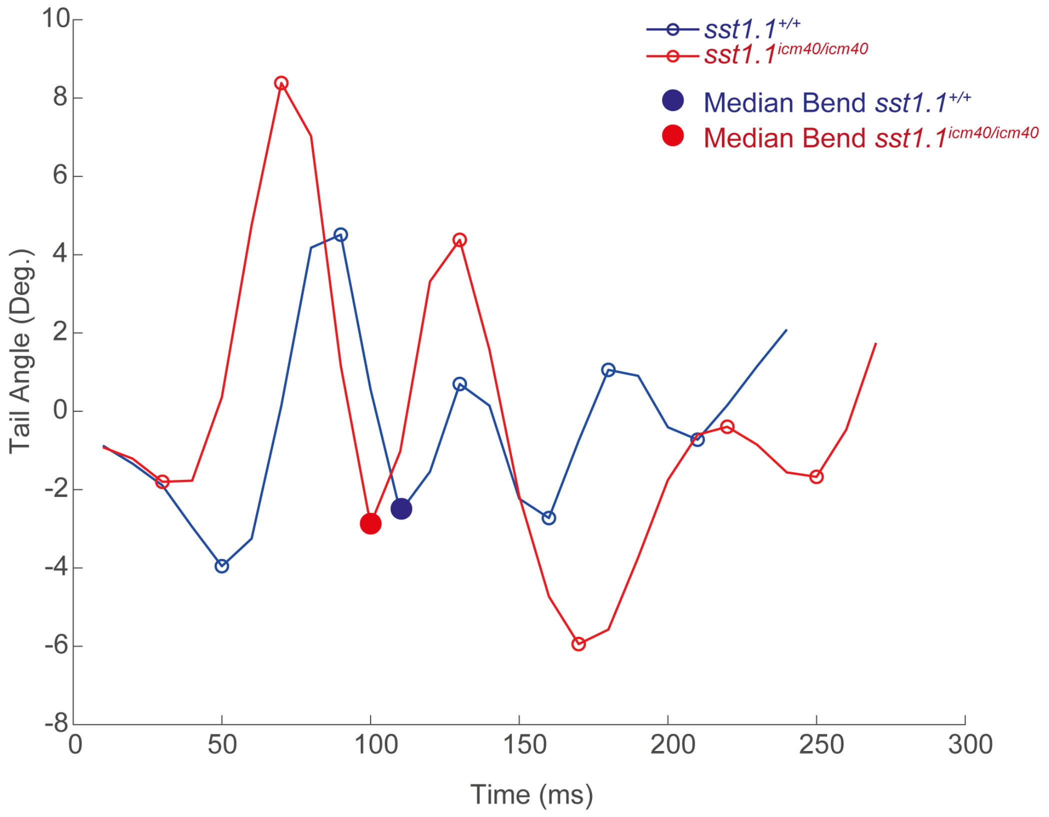

Figure S1. Example traces of typical kinematic changes between *sst1.1*$^{+/+}$ and *sst1.1*$^{icm40/icm40}$ in median bend amplitude. Example traces of forward swim bouts of *sst1.1*$^{+/+}$ and *sst1.1*$^{icm40/icm40}$ showing the typical tail angle traces over time and the amplitude of the effect observed in the mutant. Subsequent peaks (circles) correspond to each bend amplitude. Median bend of *sst1.1*$^{+/+}$ (red dot) bout (2.88 degrees) is larger than the median bend of *sst1.1*$^{icm40/icm40}$ bout (blue dot, 2.56 degrees), both values correspond the mean value of wild-type sibling group and the one of *sst1.1*$^{icm40/icm40}$ group. Bout duration for *sst1.1*$^{+/+}$ (red traces) bout is 270 ms, and bout duration for *sst1.1*$^{icm40/icm40}$ bout (blue traces) is 240 ms.

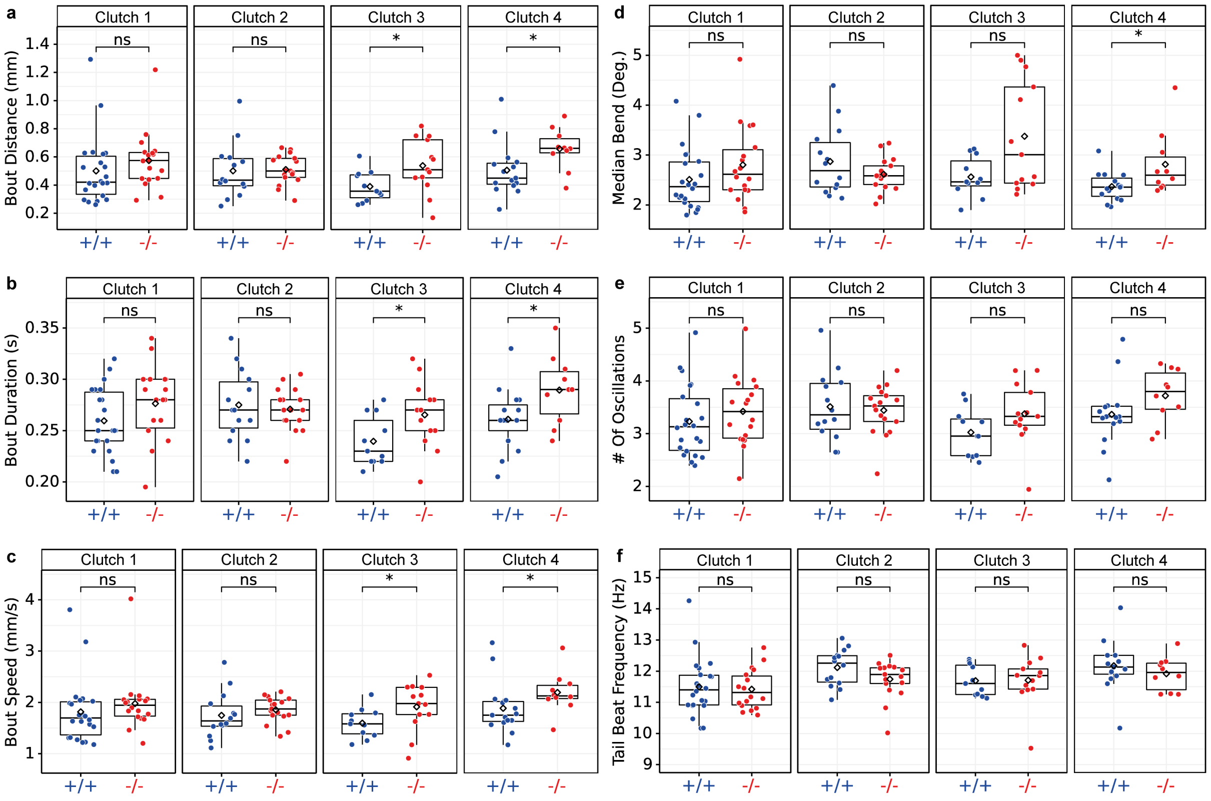

Figure S2. The comparison of kinematic parameters in forward swims between *sst1.1*$^{icm40/icm40}$ mutant and wild-type siblings for each of the 4 clutches. Comparison for individual experiment between WT larvae versus *sst1.1*$^{icm40/icm40}$ mutant was tested with Wilcoxon. Clutch 1 (22 WT larvae versus 18 *sst1.1*$^{icm40/icm40}$ mutants), clutch 2 (14 WT larvae versus 17 *sst1.1*$^{icm40/icm40}$ mutants), clutch 3 (10 WT larvae versus 13 *sst1.1*$^{icm40/icm40}$ mutants), clutch 4 (15 WT larvae versus 10 *sst1.1*$^{icm40/icm40}$ mutants). Forward swims in *sst1.1*$^{icm40/icm40}$ mutant (red, '-/-') had significant increased distance traveled **(a)** in clutch 3 (mean ± s.e.m. 0.54 ± 0.05 mm versus 0.39 ± 0.03 mm in WTs; p=0.037) and clutch 4 (mean ± s.e.m. 0.66 ± 0.05 mm versus 0.51 ± 0.05 mm in WTs; p=0.021)**,** bout duration **(b)** in clutch 3 (mean ± s.e.m. 0.27 ± 0.009 s versus 0.24 ± 0.007 s in WTs; p=0.042) and clutch 4 (mean ± s.e.m. 0.29 ± 0.011s versus 0.26 ± 0.008 s in WTs; p=0.047), speed **(c)** in clutch 3 (mean ± s.e.m. 1.91 ± 0.13 mms/ versus 1.59 ± 0.009 mm/s in WTs; p=0.049) and clutch 4 (mean ± s.e.m. 2.19 ± 0.13 mms/ versus 1.89 ± 0.13 mm/s in WTs; p=0.021) and median bend amplitude **(d)** in clutch 4 **(**mean ± s.e.m. 2.81 ± 0.20 degrees versus 2.37 ± 0.07 degrees in WTs; p = 0.028), but no significant difference in number of oscillations **(e)** and tail beat frequency **(f)** in any clutches. Black lines, median; Diamond, mean. M_{eff} = 4.003. (see Statistical Analysis Slow Swim)
